# Supplementary material for: Homogeneously catalysed conversion of aqueous formaldehyde to H2 and carbonate
Source: Nat Commun. 2017 Apr 28;8:14990. doi: 10.1038/ncomms14990 (PMC5414358; doi:10.1038/ncomms14990)
Supplement: Supplementary Information — Supplementary Figures, Supplementary Tables, Supplementary Note, Supplementary Methods and Supplementary References. [file ncomms14990-s1.pdf]

1. Supplementary Methods

All reactions and manipulations were performed under argon atmosphere using standard Schlenk and glove-box (argon atmosphere) techniques. THF, DME, hexane, and toluene were degassed and purified using an Innovative Technologies PureSolv system. Deuterated solvents (C_6D_6, THF-d_8), were obtained from Euriso-Top, dried over Na distilled by trap-to-trap transfer at low pressure and degassed by three freeze-pump-thaw cycles. All aldehydes were degassed before use. The precursor [$RuCl_2(PPh_3)_3$],[1] complexes **1K** and **3a** were prepared according to the method previously reported.[2] Formaldehyde was purchased as aqueous solution (37 wt%, Aldrich) and paraformaldehyde (97%, Alfa Aesar) were used as received. Tetrabutylammonium bromide (Bu_4NBr) and dodecyltrimethylammonium bromide ([(dodecyl)Me_3N]Br) were purchased from Aldrich and dried at 80 °C under vacuum previous use. Tetrabutylammonium formate [Bu_4N][HCO_2] was prepared according to the previously reported method.[3] NMR spectra were recorded on Bruker Avance 300, 400 and 500 MHz spectrometers. The chemical shifts (δ) are expressed in ppm relative to TMS for ^{1}H and ^{13}C respectively. Coupling constants (*J*) are given in Hertz (Hz) as absolute values. The multiplicity of the signals is indicated as s, d, t, or m for singlets, doublets, triplets, or multiplets, respectively. The abbreviation *br* is given for broadened signals. Quaternary carbon atoms are indicated as C^{quat}, aromatic units as CH^{ar} and CH^{ar} The olefinic protons and carbon atoms of the C=C_{trop} unit in the central seven-membered ring are indicated as CH^{olefin} and CH^{olefin}. The benzylic protons and carbon atoms in the central seven-membered ring are indicated as CH^{benzyl} and CH^{benzyl}. IR spectra were recorded on a *Perkin-Elmer-Spectrum 2000* FT-IR-Raman spectrometer. For solid compounds, the ATR technique was applied. The absorption bands are described as follows: strong (*s*), very strong (*vs*), middle (*m*), weak (*w*), or broad (*br*). High resolution MALDI-MS analyses were performed using a Varian HiResMALDI by ETH Zürich DCHAB/LOC MS-Service. Values are given as m/z. GC-MS analysis was done with a Trace GC Ultra and Polaris Q device from Thermo Finnigan, equipped with a Zebron ZB-5MS (30 m x 0.25 mm x 25 μm) column, Electronic Impact (ion source) and ion trap (mass analyser). Agilent 7890A gas chromatograph (Agilent Technologies) equipped with a HP Molsieve column (15 m x 0.32 mm x 25 μm) and combined with a thermal conductivity detector was used for the analysis of

the gas phase in the experiment of formaldehyde. Elemental analyses were performed by the microanalytical laboratory of the ETH Zürich. Melting points were determined with a Büchi melting point apparatus and are not corrected. X-Ray diffraction was measured on a Bruker SMART Apex II diffractometer with CCD area detector; Mo-Kα radiation (0.71073 Å) at T = 100 K. The refinement against full matrix (versus F^2) was performed with SHELXTL (ver. 6.12) and SHELXL-97. For all complexes all non-hydrogen atoms were refined anisotropically.

Supplementary Note 1. Data from webbook.nist.gov/chemistry: $\Delta_f H°_{gas}$ (CH_2O) = −115.9 kJ mol^{-1}; $\Delta_f H°_{gas}$ (H_2O) = −241.8 kJ mol^{-1}; $\Delta_f H°_{gas}$ (CO_2) = −393.5 kJ mol^{-1}; $\Delta_f H°_{gas}$ (H_3COH) = −205.0 kJ mol^{-1}; $\Delta_f H°_{gas}$ (CO) = −110.5 kJ mol^{-}.

2. Synthesis and spectroscopic data of complexes 1 - 7.

2.1. Synthesis of complexes 1.

Synthesis of [Bu_4N][Ru(trop$_2$dad)] (1Aa)

Method A. To a solution of complex **1K** (50.0 mg, 0.066 mmol, 1.0 equiv) in THF (6 mL) Bu_4NBr (31.9 mg, 0.1 mmol, 1.5 equiv) was added as solid. The suspension was stirred for 12 hours at room temperature and filtered through a pad of Celite. All volatiles of the filtrate were removed under reduced pressure and the obtained purple solid was washed carefully with Et_2O (3 x 1 mL) and 5 mL of *n*-hexane. Crystals suitable for single crystal X-ray diffraction analysis were grown from an *n*-hexane layered solution of the solid in DME / THF (1:1). Drying under high vacuum gave the product as a maroon powder. Yield: 32.6 mg, 42%. X-Ray analysis of single crystals indicated that the compound co-crystallized with one molecule of Bu_4NBr.

Method B. H_2O (24 µL, 1.32 mmol, 20 equiv) was added to a solution of complex **1K** (50.1 mg, 0.066 mmol, 1.0 equiv) in THF (6 mL) and the mixture was heated for 6 h at 60 °C under an argon stream. The initial brown solution turned gradually to dark red and the formation of complex [Ru(trop$_2$dad)] (**2**) was observed spectroscopically. After filtration, all volatiles were removed. The obtained residue was washed with *n*-hexane (2 mL) and dried under vacuum to give pure complex **2** as a dark red solid. Yield: 28.3 mg, 80%. ^{1}H NMR (500 MHz, THF-d_8, 298K): δ [ppm] = 8.40 (s, 2H, N=C*H*), 7.28-7.23 (m, 8H, CH^{ar}), 7.09-6.78 (m, 8H, CH^{ar}), 5.66 (s, 2H, CH^{benzyl}), 4.59 (s, 4H, CH^{olefin}). ^{13}C{^{1}H} NMR (125 MHz, THF-d_8, 298K): δ [ppm] = 147.5 (s, *C*H=N), 145.7 (s, C^{quat}), 138.5 (s, C^{quat}), 127.9 (s, CH^{ar}), 127.6 (s, CH^{ar}), 127.4 (s, 2 CH^{ar}), 127.0 (s, CH^{ar}), 126.6 (s, CH^{ar}), 126.3 (s, 2CH^{ar}), 123.7 (s, CH^{ar}), 78.5 (s, CH^{benzyl}), 72.1 (s, CH^{olef}).[2]

[Bu_4N][HCO_2] (8.8 mg, 0.031 mmol, 1.1 equiv) was added to a solution of complex **2** (15 mg, 0.028 mmol, 1.0 equiv) in THF (3 mL) and the mixture was stirred at room temperature for 1h, causing a colour change from red to violet. The solution was filtrated through a syringe filter (50 µm porosity). The obtained clear violet filtrate was layered with *n*-hexane and cooled to -32 °C. After two days, air sensitive burgundy red crystals of complex **1Aa** were isolated by filtration and dried in a stream of argon. Yield: 12.2 mg, 51%. ^{1}H NMR (500 MHz, d_8-THF, 298K): δ [ppm] = 7.22 (dd, $^3J_{HH}$ = 7.6, $^4J_{HH}$ = 1.3 Hz, 2H, CH^{ar}), 7.13 (dd, $^3J_{HH}$ = 7.6, $^4J_{HH}$ = 1.3 Hz, 2H, CH^{ar}), 6.92 (dd, $^3J_{HH}$ = 7.4, $^4J_{HH}$ = 1.4 Hz, 2H, CH^{ar}), 6.89 (dd, $^3J_{HH}$ = 7.4, $^4J_{HH}$ = 1.4 Hz,

2H, CH^{ar}), 6.84 (td, $^{3}J_{HH}$ = 7.5, $^{4}J_{HH}$ = 1.4 Hz, 2H, CH^{ar}), 6.77 (td, $^{3}J_{HH}$ = 7.4, $^{4}J_{HH}$ = 1.4 Hz, 2H, CH^{ar}), 6.58 (td, $^{3}J_{HH}$ = 7.4, $^{4}J_{HH}$ = 1.3 Hz, 2H, CH^{ar}), 6.55 (td, $^{3}J_{HH}$ = 7.3, $^{4}J_{HH}$ = 1.3 Hz, 2H, CH^{ar}), 6.52 (s, 2H, N=C*H*), 5.05 (s, 2H, CH^{benzyl}), 3.20 – 3.00 (m, 8H, 4 CH_2), 3.02 – 2.92 (m, 4H, CH^{olefin}), 1.55 – 1.21 (m, 16H, 8 CH_2), 0.99 (t, $^{3}J_{HH}$ = 7.2 Hz, 12H, 3 CH_3), -9.37 (RuH). $^{13}C\{^{1}H\}$ NMR (126 MHz, d_8-THF, 298K) δ[ppm] = 149.3 (s, C^{quat}), 147.5 (s, C^{quat}), 146.4 (s, C^{quat}), 144.1 (s, C^{quat}), 126.6 (s, *C*H^{ar}), 125.9 (s, *C*H^{ar}), 125.6 (s, *C*H^{ar}), 125.4 (s, *C*H^{ar}), 125.2 (s, N=*C*H), 125.0 (s, *C*H^{ar}), 124.5 (s, *C*H^{ar}), 120.9 (s, *C*H^{ar}), 119.2 (s, *C*H^{ar}), 77.5 (s, *C*H^{benzyl}), 64.6 (s, *C*H^{olefin}), 57.9 (s, *C*H_2), 52.9 (s, *C*H^{olefin}), 31.5 (s, *C*H_2), 22.5 (s, *C*H_2), 19.7 (s, *C*H_2), 13.2 (s, *C*H_3).

ATR IR (ν in cm^{-1}): 3054 (w), 2951 (w). 1944 (m, ν Ru-H), 1589 (m), 1480 (m), 1431 (m), 1393 (w), 1300(w), 1279 (w), 1222 (w), 1182 (w), 1137 (w), 1106 (m), 1089 (m), 1044 (m), 881 (w), 863 (w), 841 (w), 743 (s), 693 (s).

mp = >220 °C (dec.)

EA Calcd. For $C_{48}H_{61}N_3Ru.C_4H_8O$: C 73.20, H 8.15, N 4.93; found C 72.97, H 8.31, N 4.65.

Synthesis of [(dodecyl)Me_3N][Ru(trop$_2$dad)] (1Ab)

To a solution of complex **1K** (50 mg, 0.066 mmol, 1.0 equiv) in THF (6 mL) [(dodecyl)Me_3N]Br (22.4 mg, 0.072 mmol, 1.1 equiv) was added as solid. The suspension was stirred for 3 hours at room temperature and filtered through a pad of Celite. All volatiles of the filtrate were removed under reduced pressure and the crude dark purple product was washed carefully with Et_2O (3 x 1 mL) and 5 mL of *n*-hexane. Drying the residue under high vacuum gave the product as air sensitive dark red powder. Yield: 41.5 mg, 82 %. Crystals suitable for single crystal X-ray diffraction analysis were grown from an *n*-hexane layered solution of the product in a DME / THF (1:1) mixture.

^{1}H NMR (400 MHz, d_8-THF, 298K): δ[ppm] = 7.51-6.90 (m, 14H, CH^{ar}), 6.80 – 6.67 (m, 2H, CH^{ar}), 6.67 – 6.59 (m, 2H, N=C*H*), 5.06 (s, 2H, CH^{benzyl}), 3.58 (brs, 2H, CH_2), 3.54 – 3.50 (m, 2H, CH^{olefin}), 3.44 (m, 2H, CH^{olefin}, overlapped by CH_3 signal), 3.43 (s, 9H, CH_3), 1.73 (brs, 2H, CH_2), 1.39-1.26 (m, 18H, CH_2), 0.91 (t, $^3J_{HH}$ = 6.8 Hz, 3H, CH_3), -9.54 (s, 1H, RuH). ^{13}C{^{1}H} NMR (101 MHz, d_8-THF, 298K): δ [ppm] =148.6 (s, C^{quat}), 147.9 (s, C^{quat}), 146.4 (s, C^{quat}), 143.9 (s, C^{quat}), 125.9 (s, CH^{ar}), 125.7 (s, 2 CH^{ar}), 125.0 (s, CH^{ar}), 123.5 (s, 2 NCH), 122.0 (s, CH^{ar}), 120.3 (s, CH^{ar}), 121.3 (s, CH^{ar}), 119.6 (s, CH^{ar}), 77.1 (s, CH^{benzyl}), 71.8 (s, CH^{olefin}), 70.3 (s, CH^{olefin}), 66.6 (s, CH_2), 57.9 (s, 3 CH_3), 51.4 (s, CH_2), 29.6 -26.3 (s, 8 CH_2), 22.6 (s, CH_2), 13.53 (s, CH_3).

ATR IR (ν in cm^{-1}): 2981 (w). 1956 (m), 1580 (m), 1480 (m), 1442 (m), 1399 (w), 1301 (w), 1256 (w), 1223 (w), 1185 (w), 1145 (w), 1110 (w), 1045 (m), 891 (w), 843 (m), 822 (m), 745 (s), 693 (s), 642 (s).

mp = >220 °C (dec.)

EA Calcd. for $C_{47}H_{59}N_3Ru$: C 73.59, H 7.75, N 5.48; found C 73.89, H 7.98, N 5.25.

Synthesis of [K(db-18C6)][Ru(trop$_2$dad)] (1KC)

To a solution of **1K** (20.0 mg, 0.026 mmol, 1.0 equiv) in THF (2 mL) dibenzo-18-crown-6 ether (9.3 mg, 0.026 mmol, 1.0 equiv) was added. The mixture turned from dark brown to deep yellow-brown after stirring for few minutes at room temperature. Subsequently, 1 mL Et_2O was added to the solution. Layering the solution with 3 mL of *n*-hexane afforded a dark orange solid after 12 h at -32 °C. The mother liquor was decanted and the air sensitive solid was washed with cold Et_2O (3 x 1 mL) and dried under vacuum. Yield: 16.8 mg, 69%.

^{1}H NMR (500 MHz, d_8-THF, 298K): δ[ppm] = 7.23 (d, $^3J_{HH}$ = 7.6 Hz, 2H, CH^{ar}), 7.14 (d, $^3J_{HH}$ = 7.6 Hz, 2H, CH^{ar}), 6.94-6.89 (m, 8H, CH^{ar} K(db18c6)), 6.87 (m, 4H, CH^{ar}), 6.77 (m, 2H, CH^{ar}), 6.72 (m, 2H, CH^{ar}), 6.57 (s, 2H, N=C*H*), 6.52 (m, 2H, CH^{ar}), 6.45 (m, 2H, CH^{ar}), 5.05 (s, 2H, CH^{benzyl}), 3.77 (m, 8H, CH_2 K(db18c6)), 3.37 (m, 8H, CH_2 K(db18c6)), 3.06 (d, $^3J_{HH}$ = 8.7 Hz, 2H, CH^{olefin}), 2.96 (d, $^3J_{HH}$ = 8.7 Hz, 2H, CH^{olefin}), -9.00 (s, 1H, Ru*H*).

^{13}C{^{1}H} NMR (126 MHz, d_8-THF, 298K): δ[ppm] = 150.1 (C^{quat}), 148.7 (C^{quat}), 147.8 (C^{quat} K(db18c6)), 147.4 (C^{quat}), 145.2 (C^{quat}), 127.2 (CH^{ar}), 126.5 (CH^{ar}), 126.2 (CH^{ar}), 125.9 (2 CH^{ar}), 125.7 (2 N=CH^{ar}), 125.2 (CH^{ar}) 121.7 (CH^{ar} K(db18c6)), 121.4 (CH^{ar}) 119.9 (CH^{ar}) 112.1 (CH^{ar} K(db18c6)), 78.4 (CH^{benzyl}), 69.2 (CH$_2$ K(db18c6)), 67.9 (CH$_2$ K(db18c6)), 64.7 (CH^{olefin}), 53.8 (CH^{olefin}).

ATR IR (ν in cm^{-1}): 2295 (br), 1998 (m), 1545 (m), 1477 (s), 1450 (s), 1363 (m), 1296 (m), 1270 (m), 1247 (m), 1212 (m), 1199 (m), 1120 (m), 1081 (vs), 1040 (s), 845 (s), 822 (s), 734 (vs), 725 (s), 673 (m), 635 (m).

mp = >220 °C (dec.)

EA Calcd. for $C_{52}H_{49}KN_2O_6Ru$: C 66.58, H 5.26, N 2.99; found C 66.23, H 5.99, N 3.15.

2.2. Synthesis of [Ru(trop$_2$dad)(CO)Ru(trop$_2$dad)].thf (5)

Complex **2** (prepared as described above) (0.5 mmol, 1.0 equiv) was dissolved in 20 mL THF. The solution was placed in a Schlenk flask with a J. Young valve. Argon was purged through the solution by the freeze-pump-thaw method (three times), and the flask was subsequently filled with CO(g) (1.0 bar). The colour changed from dark brown to orange within seconds, accompanied by the precipitation of a microcrystalline orange solid, identified as complex **5**. The reaction mixture was filtered and the filtrate was layered with *n*-hexane (6 mL) and stored at –32°C. After 2 days, a second crop of orange crystals of **5**, suitable for x-ray diffraction analysis were isolated by filtration and dried in a stream of argon. Combined yield: 235 mg, 80%.

^{1}H NMR (400 MHz, d_8-THF, 298K) δ [ppm] = 7.64 (dd, J_{HH} = 8.0, 1.3 Hz, 1H, CH^{ar}), 7.56 – 6.98 (m, 35H, N=CH, CH^{ar} and 2CH^{olefin}), 6.92 (m, 1H, N-CH), 6.47 (d, J_{HH} = 8.0 Hz, 1H, N-CH), 6.25 (d, J_{HH} = 10.2 Hz, 1H, CH^{olef}), 5.72 (s, 1H, CH^{benzyl}), 5.43 (s, 2H, CH^{benzyl}), 5.01 (s, 1H, CH^{benzyl}), 4.97 (d, J_{HH} = 8.9 Hz, 1H, CH^{olefin}), 4.41 (m, 1H, N-CH), 4.28 – 4.11 (m, 2H, CH^{olefin}), 3.95 (d, J_{HH}= 8.9 Hz, 1H, CH^{olefin}), 3.60 (1H, CH^{olefin}, overlapped with THF signal). ^{13}C{^{1}H} NMR (101 MHz, d_8-THF, 298K) δ [ppm] = δ 201.9 (CO), 172.7 (CHN), 143.3 (C^{quat}), 142.9 (C^{quat}), 142.0 (C^{quat}), 141.8 (C^{quat}), 141.1 (C^{quat}), 139.7 (C^{quat}), 139.6 (C^{quat}), 138.8 (N=CH), 138.7 (C^{quat}), 138.0 (C^{quat}), 137.9 (C^{quat}), 134.7 (CH^{ar}), 132.8 (CH^{ar}), 130.3 (CH^{olefin}), 129.8 (CH^{ar}), 128.7 (CH^{ar}), 128.5 (CH^{ar}), 128.1 (CH^{ar}), 127.8 (CH^{ar}), 127.8 (CH^{ar}), 127.6 (CH^{ar}), 127.3 (CH^{ar}), 127.1 (CH^{ar}), 126.9 (CH^{ar}), 126.8 (CH^{ar}), 126.7(CH^{ar}), 126.6 (CH^{ar}), 126.4 (CH^{ar}), 125.5 (CH^{ar}), 125.4 (CH^{ar}), 125.1 (CH^{ar}), 125.0 (CH^{ar}), 124.8 (CH^{ar}), 124.7 (CH^{ar}), 124.5 (CH^{ar}), 92.4 (CHN), 81.1 (CH^{olefin}), 79.6 (CH^{benzyl}), 78.0 (2CH^{benzyl}), 74.5 (CH^{olefin}), 73.19 (CH^{benzyl}), 61.0 (CH^{olefin}), 56.7 (CH^{olefin}), 55.8 (CH^{olefin}).

ATR IR (ν in cm^{-1}): 3250 (w), 3240 (w), 2932 (w), 2320 (w), 1912 (s), 1588 (m), 1495 (w), 1467 (w), 1443 (w), 1390 (w), 1330 (w), 1034 (w), 1012 (w), 990 (w), 932 (w), 915 (w), 876 (w), 849 (w), 747 (s), 723 (m), 698 (m), 637 (s).

mp = 182-184 °C (dec.)

EA Calc. for $C_{69}H_{55}N_4O_2Ru_2$: C 70.57, H 4.72, N 4.77; found: C 70.48, H 4.98, N 4.55

2.3. Synthesis of [Ru(trop$_2$dad)(CO)] (3b)

All volatiles of the previous mother liquor (section 2.2) were removed under reduced pressure and the red residue was washed carefully with Et_2O (1 mL) and *n*-hexane (1 mL). After drying under high vacuum, the air sensitive compound **3b** was isolated as a red powder. Yield: 27.8 mg, 10%.

^{1}H NMR (500 MHz, *d*$_8$-THF, 298K) δ [ppm] = 7.45 (d, J_{HH} = 7.6 Hz, 2H, CH^{ar}), 7.37 – 7.32 (m, 4H, overlapped CH^{ar} and N=CH), 7.27 (d, J_{HH} = 7.5 Hz, 2H, CH^{ar}), 7.15 (tt, J_{HH}= 8.5, 1.1 Hz, 4H, CH^{ar}), 7.03 – 6.94 (m, 4H, CH^{ar}), 6.88 (tt, J_{HH} = 7.5, 1.1 Hz, 2H, CH^{ar}), 5.68 (s, 2H, CH^{benzyl}), 4.20 (dd, J_{HH} = 9.3, 2.6 Hz, 2H, CH^{olefin}), 4.12 (d, J_{HH} = 9.2 Hz, 2H, CH^{olefin}). ^{13}C{^{1}H} NMR (126 MHz, *d*$_8$-THF, 298K) δ [ppm] = δ 208.1 (CO), 142.9 (C^{quat}), 141.8 (C^{quat}), 139.7 (C^{quat}), 139.6 (C^{quat}), 137.9 (N=*C*H), 127.6 (*C*H^{ar}), 127.0 (*C*H^{ar}), 127.0(*C*H^{ar}), 126.7 (*C*H^{ar}), 126.4 (*C*H^{ar}), 125.0 (*C*H^{ar}), 124.9 (*C*H^{ar}), 124.7 (*C*H^{ar}), 78.0 (CH^{benzyl}), 74.5 (CH^{olefin}), 61.0 (*C*H^{olefin}).

ATR IR (ν in cm^{-1}): 3255 (w), 3246 (w), 3225 (w), 2990 (w), 2920 (w), 1904 (s), 1597 (m), 1580 (w), 1497 (w), 1445 (w), 1389 (w), 1342 (w), 1032 (w), 1024 (w), 1005 (w), 993 (w), 965 (w), 935 (w), 886 (w), 880 (w), 854 (w), 790 (m), 755 (s), 746 (m),723 (m), 693 (m), 641 (s), 631(m).

mp = 154-156 °C (dec.)

EA Calc. for $C_{33}H_{24}N_2ORu$: C 70.07, H 4.28, N 4.95; found: C 70.26, H 4.49, N 4.92.

2.4. Reactivity of complexes 1 towards formaldehyde/H_2O mixtures under base-free conditions: Synthesis of complexes 4 and $5H_2$.

Synthesis of [Ru($trop_2$dae)] (4)

An aqueous solution of HCOH (37 wt%, 32 μL, 0.420 mmol, 8.0 equiv) was added to a solution of complex **1K** (39.4 mg, 0.052 mmol, 1.0 equiv) or **1Aa** (40.6 mg, 0.052 mmol, 1.0 equiv) in degassed d_8-THF (0.6 mL) under an argon flow. The solution was analysed using NMR spectroscopy. After 12h at rt, compound **4** was detected spectroscopically as the main product.

^{1}H NMR (400 MHz, d_8-THF, 298K): δ [ppm] = 7.50 (d, $^3J_{HH}$ = 6.7 Hz, 2H, CH^{ar}), 7.40 (d, $^3J_{HH}$ = 7.2 Hz, 2H, CH^{ar}), 7.09-7.04 (m, 4H, CH^{ar}), 6.99-6.95 (m, 4H, CH^{ar}), 6.92-6.87 (m, 4H, CH^{ar}), 5.22 (br, 1H, N*H*), 5.20 (br, 1H, N*H*), 4.51 (s, 2H, CH^{benzyl}), 2.82 (dd, $^3J_{HH}$ = 7.3 Hz, $^3J_{HH}$ = 3.7 Hz, 2H, CH_2), 2.69 (d, $^3J_{HH}$ = 7.8 Hz, 2H, CH^{olef}), 2.22 (dd, $^3J_{HH}$ = 11.9 Hz, $^3J_{HH}$ = 7.5 Hz, 2H, CH_2), 1.74 (d, $^3J_{HH}$ = 8.1 Hz, 2H, CH^{olef}); ^{13}C{^{1}H} NMR (101 MHz, d_8-THF, 298K): δ [ppm] = 149.5 (s, C^{quat}), 147.4 (s, C^{quat}), 136.2 (s, C^{quat}), 136.0 (s, C^{quat}), 129.5 (s, *C*H^{ar}), 128.2 (s, *C*H^{ar}), 128.0 (s, *C*H^{ar}), 127.5 (s, *C*H^{ar}), 127.2 (s, *C*H^{ar}), 126.5 (s, *C*H^{ar}), 122.0 (s, *C*H^{ar}), 120.9 (s, *C*H^{ar}), 69.8 (s, *C*H^{benzyl}), 59.2 (s, *C*H^{olef}), 49.1 (s, *C*H$_2$), 49.2 (s, *C*H$_2$),48.8 (s, *C*H^{olef}).[2]

Synthesis of [Ru($trop_2$dad)Ru(trop-dad-trop^H)(CO)] ($5H_2$)

The previous solution was extracted under argon with 0.6 mL of degassed D_2O and the aqueous phase was analysed by ^{1}H and ^{13}C NMR, revealing the presence of HCO_2M and M_2CO_3 (M = K, Bu_4N). The organic phase was concentrated to dryness under vacuum. The obtained dark orange was dissolved in 2 mL of THF / DME (1:1) and layered with *n*-hexane (1 mL). After 5 days at -32 °C the compound **5H$_2$** was obtained as orange crystals. The mother liquor was decanted and the obtained air sensitive crystals were washed with *n*-hexane prior drying in a stream of argon. Yield: 3.1 mg, 10%.

^{1}H NMR (400 MHz, d_8-THF) δ[ppm] = δ 7.59 (d, $^3J_{HH}$ = 7.6 Hz, 1H, CH^{ar}), 7.48 (d, $^3J_{HH}$ = 7.1 Hz, 1H, CH^{ar}), 7.41 (d, $^3J_{HH}$ = 7.5 Hz, 1H, CH^{ar}), 7.36 – 7.22 (m, 6H, CH^{ar}), 7.18 – 7.01 (m, 12H, CH^{ar}), 7.00 – 6.88 (m, 4H, CH^{ar}, 1H, NC*H*=), 6.87 – 6.77 (m, 3H, CH^{ar}, 1H, -NC*H*=), 6.73 (td, $^3J_{HH}$ = 7.5, $^4J_{HH}$ = 1.4 Hz, 1H, CH^{ar}), 6.64 (td, $^3J_{HH}$ = 7.3, $^4J_{HH}$ = 1.6 Hz, 2H, CH^{ar}), 6.27 (td, $^3J_{HH}$ = 7.4, $^4J_{HH}$ = 1.4 Hz, 1H), 5.70 (s, 1H, CH^{benzyl}), 5.6 (s, 1H, CH^{benzyl}),

4.99 (s, 1H, CH^{benzyl}), 4.97 (s, 1H, CH^{benzyl}), 4.76 (d, $^3J_{HH}$ = 9.3 Hz, 1H, CH^{olefin}), 4.59 (d, $^3J_{HH}$ = 9.1 Hz, 1H, CH^{olefin}), 4.56-4.54 (m, 1H, N-C*H*), 4.32 (dd, $^3J_{HH}$ = 20.3, $^4J_{HH}$ = 1.3 Hz, 2H, CH^{olefin}), 4.11 (d, $^3J_{HH}$ = 9.1 Hz, 1H, CH^{olefin}), 4.04 (d, $^3J_{HH}$ = 9.3 Hz, 1H, CH^{olefin}), 3.39 (d, $^3J_{HH}$ = 9.1 Hz, 1H, N-C*H*), 3.31 (dt, $^2J_{HH}$ = 17.4, $^3J_{HH}$ = 3.3 Hz, 1H, CH_2), 2.64 – 2.44 (m, 1H), 2.07 – 1.88 (m, 1H, CH_2), 1.80 (m, 1H, CH_2, overlapped by THF signal).

^{13}C{^{1}H} NMR (101 MHz, d_8-THF, 298K) δ[ppm] = 201.8 (CO), 175.2 (N=*C*H), 173.4 (N=*C*H), 146.0 (C^{quat}), 145.0 (C^{quat}), 144.1 (C^{quat}), 143.7 (C^{quat}), 143.4 (C^{quat}), 142.4 (C^{quat}), 141.9 (C^{quat}), 141.2 (C^{quat}), 140.7 (C^{quat}), 140.2 (C^{quat}), 140.1 (C^{quat}), 139.5 (C^{quat}), 139.2 (C^{quat}), 139.1 (C^{quat}), 138.3 (C^{quat}), 136.5 (C^{quat}), 134.6 (CH^{ar}), 134.3 (CH^{ar}), 131.9 (CH^{ar}), 130.9 (CH^{ar}), 130.4 (CH^{ar}), 130.3 (CH^{ar}), 129.9 (CH^{ar}), 129.2 (CH^{ar}), 128.3 (CH^{ar}), 127.5 (CH^{ar}), 127.3 (CH^{ar}), 126.6 (CH^{ar}), 126.0 (CH^{ar}), 125.6 (CH^{ar}), 124.8 (CH^{ar}), 124.5 (CH^{ar}), 124.2 (CH^{ar}), 81.9 (*C*HN), 81.8 (*C*HN), 80.7 (CH^{benzy}), 80.4 (CH^{benzy}), 79.9 (CH^{benzy}), 79.8 (CH^{benzy}), 74.4 (CH^{olef}), 73.7 (CH^{olef}), 73.3 (CH^{olef}), 72.6 (N-*C*H), 72.4 (N-*C*H), 71.2 (CH^{olef}), 60.5 (CH^{olef}), 56.9 (CH^{olef}), 36.4 (CH_2), 31.9 (CH_2).

ATR IR (ν in cm^{-1}): 3140 (w), 2995 (w), 2978 (w), 2154 (w), 1918 (s), 1604 (w), 1490 (w), 1475 (w), 1440 (w), 1395 (w), 1330 (w), 1295 (m), 1241 (w), 1225 (m), 1218 (m), 1152 (m), 1110 (w), 1093 (w), 1053 (w), 1022 (s), 1002 (w), 995 (w), 975 (w), 935 (w), 910 (w), 874 (w), 836 (w), 829 (w), 756 (m), 744 (s), 722 (s), 693 (m), 645 (s).

mp = >163-165 °C (dec.)

EA Calc. for $C_{65}H_{50}N_4ORu_2$: C 70.63, H 4.56, N 5.07; found: C 70.23, H 4.89, N 5.12

2.5. Synthesis of [Ru(trop-dad-trop^H)(CO)(PPh_3)] (6).

A solution of complex **3a** (196 mg, 0.22 mmol, 1.0 equiv) in degassed THF (50 mL) was placed in a Schlenk flask equipped with a J. Young valve. Subsequently a 37 wt.% solution of HCOH in water (33 μL, 0.44 mmol, 2 equiv) was added and the mixture was stirred at 60 °C during 3h. The resulting deep blue solution was filtrated and the filtrate was evaporated to approx. 10 mL. Afterwards, a few drops of DME and 10 mL *n*-hexane were added. Dark blue crystals of complex **6** were obtained after slow evaporation of the solvent. The air sensitive crystals were washed with *n*-hexane and dried under argon. Yield: 155 mg, 85%.

^{1}H NMR (500 MHz, d_8-THF, 298K) δ [ppm] = 7.32-7.29 (m, 7H, 2N=C*H* and CH^{ar}), 7.19 – 6.66 (m, 23H, CH^{ar}), 6.61 (q, $^3J_{HH}$ = 7.1 Hz, 1H, CH^{ar}), 6.45 (d, $^3J_{HH}$ = 7.5 Hz, 1H, CH^{ar}), 6.09 (d, $^3J_{HH}$ = 7.6 Hz, 1H, CH^{ar}), 5.23 (d, $^3J_{PH}$

= 9.1 Hz, 1H, CH^{benzyl}), 5.04 (brs, 1H, CH^{benzyl}), 4.10 (dd, $^3J_{HH}$ = 9.0, $^3J_{PH}$ = 3.2 Hz, 1H, CH^{olefin}), 3.56 (1H, CH^{olefin} overlapped by the THF signal), 2.96 – 2.83 (m, 2H, CH_2), 2.72-2.65 (m, 1H, CH_2), 2.26 – 2.10 (m, 1H, CH_2). ^{13}C{^{1}H} NMR (126 MHz, d_8-THF, 298K) δ [ppm] = 208.5 (CO), 144.8 (d, J_{PC} = 2.8 Hz, C^{quat}), 142.7 (d, J_{PC} = 3.6 Hz, C^{quat}) 142.2 (d, J_{PC} = 2.6 Hz, C^{quat}), 141.6 (d, J_{PC} = 12.1 Hz, N-*C*H), 140.5 (s, C^{quat}), 139.1 (s, C^{quat}), 138.9 (s, 2 C^{quat}), 138.5 (s, C^{quat}), 138.2 (s, C^{quat}), 138.1 (s, 2 C^{quat}), 133.8 (CH^{ar}), 133.5 (CH^{ar}), 133.1 (d, J_{PC} = 9.9 Hz, C^{quat}), 132.4 (s, 2 CH^{ar}), 132.3 (s, 2 CH^{ar}), 131.6 (s, CH^{ar}), 130.9 (s, CH^{ar}), 129.8 (s, CH^{ar}), 129.1 (s, CH^{ar}), 128.6 (s, 2 CH^{ar}), 127.7 (s, 2CH^{ar}), 127.6 (s, 2 CH^{ar}), 127.4 (d, J_{PC} = 5.7 Hz, CH^{ar}), 127.2 (s, CH^{ar}), 127.0 (d, J_{PC} = 9.2 Hz, C^{quat}), 126.8 (s, CH^{ar}), 126.4 (s, CH^{ar}), 125.7 (s, 2 CH^{ar}), 125.4 (s, CH^{ar}), 123.9 (d, J_{PC} = 9.2 Hz, CH^{ar}), 123.9 (d, J_{PC} = 8.9 Hz, C^{quart}), 122.4 (s, CH^{ar}), 81.1 (CH^{benzy}), 77.8 (CH^{benzy}), 61.8 (CH^{olef}), 54.6 (CH^{olef}), 33.0 (CH_2), 28.7 (CH_2). ^{31}P{^{1}H} NMR (203 MHz, d_8-THF, 298K) δ[ppm] = 48.79 (s, 1P).

ATR IR (ν in cm^{-1}): 3021 (w), 2848 (w), 2794 (w), 2453 (w), 2348 (w), 2168 (w), 1936 (s),1597 (w), 1478 (s), 1456 (w), 1413 (w), 1363 (w), 1304 (w), 1270 (w), 1259 (w), 1184 (w), 1161 (w), 1136 (w), 1102 (w), 1044 (m), 957 (w), 946 (w), 912 (w), 878 (w), 819 (w), 776 (w), 765 (w), 738 (m), 725 (m), 654 (w), 625 (w).

mp = 180-182 °C (dec.)

EA Calc. for $C_{51}H_{41}N_2OPRu$: C 73.81, H 4.98, N 3.38; found: C 73.56, H 4.75, N 3.91.

2.6. Synthesis of [Ru(trop$_2$dae)(PPh$_3$)] (7)

Method A. A solution of H_2CO in water (37 wt%, 99 μL, 1.20 mmol, 10 equiv) was added to the blue solution of complex **6** (100 mg, 0.120 mmol, 1.0 equiv) in THF (10 mL), followed by the addition of KOH (67.3 mg, 1.20 mmol, 10.0 equiv). The resulting mixture was heated under an argon flow at 60 °C for 12h. Analysis by ^{31}P NMR revealed almost complete conversion of the starting material (90% conv.). Complex **3a** is obtained as main product along with O=PPh$_3$ (ca. 20%) and two minor additional P containing species. ^{1}H NMR analysis of a dried aliquot which was dissolved in d_8-THF indicated the formation of complex **1K** and other hydride containing complexes. The deep orange solution was extracted under an argon atmosphere with D_2O (2 x 0.5 mL) and analysed. The orange organic phase was evaporated to dryness and the residue was washed with diethyl ether. The obtained orange solid was dissolved in DME (0.5 mL), filtered and layered with *n*-hexane (1 mL). After 1 day at room temperature air

sensitive orange reddish single crystals of **7** were isolated by filtration, washed with *n*-hexane and dried in a stream of argon. Yield: 11.6 mg, 12% yield.

^{1}H NMR (400 MHz, d_8-THF, 298K) δ [ppm] = 7.33-7.20 (m, 5H, CH^{ar}), 7.10-6.98 (m, 11H, CH^{ar}), 6.89-6.83 (m, 8H, CH^{ar}), 6.80-6.76 (m, 3H, CH^{ar}), 6.72-6.64 (m, 4H, CH^{ar}), 6.45 (t, $^{3}J_{PH}$ = $^{3}J_{HH}$ = 7.0 Hz, 2H, CH^{olefin}), 6.16 (d, $^{3}J_{HH}$ = 6.8 Hz, 2H, CH^{olefin}), 4.12 (s, 2H, CH^{benzyl}), 3.17 (br, 2H, CH_2), 2.50 (br, 2H, N*H*), 2.30 (br, 2H, CH_2). ^{1}H NMR (400 MHz, d_8-THF, 233K) δ [ppm] =7.70 – 6.55 (m, 32H, 31CH^{ar}, 1CH^{olefin}), 6.37 (d, $^{3}J_{HH}$ = 7.6 Hz, 1H, CH^{olefin}), 6.27 (brs, 1H, CH^{olefin}), 5.79 (d, $^{3}J_{HH}$ = 7.7 Hz, 1H, CH^{olefin}), 4.24 (s, 1H, CH^{benzyl}), 4.10 (s, 1H, CH^{benzyl}), 4.04 (brs, 1H, N*H*), 2.77 (br, 1H, N*H*), 2.51 (d, J=9.3 Hz, 1H, C*H*2), 2.33 – 2.31 (d, 2H, C*H*2), 2.08 (d, 1H, J=10.0 Hz, C*H*2) .^{13}C{^{1}H} NMR (100.6 MHz, d_8-THF, 298K): δ [ppm] = 146.4 (s, C^{quat}), 141.6 (s, C^{quat}), 137.6 (br, C^{quat}), 137.4 (br, C^{quat}), 133.3 (br, CH^{ar}), 127.5 (br, CH^{ar}), 127.3 (br, CH^{ar}), 127. 2 (br, CH^{ar}), 127.1 (br, CH^{ar}), 126.8 (br, CH^{ar}), 121.2 (br, CH^{ar}), 120.9 (br, CH^{ar}), 69.5 (s, CH^{benzyl}), 50.8 (s, 2CH$_2$). ^{31}P{^{1}H} NMR (162.0 MHz, d_8-THF, 298K) = 50.9 (s).

ATR IR (ν in cm^{-1}): 3261 (w), 3037 (w), 2984 (w), 2872 (w), 1940 (s), 1594 (m), 1571 (w), 1483 (m), 1456 (m), 1429 (m), 1413 (w), 1397 (w), 1366 (w), 1303 (w), 1263 (w), 1242 (w), 1212 (w), 1185 (w), 1154 (w), 1103 (s), 1083 (s), 1060 (m), 1028 (m), 1014 (m), 969 (m), 934 (m), 917 (m), 881 (m), 867 (m), 851 (m), 748 (vs), 739 (vs), 722 (m), 695 (vs), 661 (w), 637 (w), 631 (w), 612 (w).

HRMS (m/z): [M-H]$^{+}$ Calcd. for $C_{50}H_{42}N_2PRu$: 803.2129; found:803.2137.

Method B. A solution of [Ru(trop$_2$dad)(PPh$_3$)] (5.5 mg, 7 µmol) in 0.7 mL of d_8-THF was placed in a NMR tube equipped with a J. Young valve. The tube was purged with argon by freeze-pump-thaw cycles (three times). Afterwards, the tube was filled with H_2 (2.0 - 4.0 bar). The reaction mixture was heated at 65 °C during 12h. The colour changed from purple to orange affording complex **7** as only product observed spectroscopically.

Supplementary Table 1. Selected ^{1}H and ^{13}C chemical shifts of complexes **1** - **7**, measured in d_8-THF. Chemical shifts are given on the δ scale are expressed in ppm.

	δ ^{1}H RuH	δ $^1H_{HC=N}$ or (N-CH2-)	δ $^{13}C_{HC=N}$ or (N-CH2-)	δ $^1H_{ol}$	δ $^{13}C_{ol}$
**1K**	-10.25	6.64	120.3,122.0	3.22, 3.13	66.5, 52.8
**1Aa**	-9.37	6.52	125.2	3.02, 2.92	64.6, 52.9
**1Ab**	-9.54	6.59	123.5	3.52, 3.44	71.8, 70.3
**1KC**	-9.00	6.57	125.7	3.06, 2.96	64.7, 53.8
**2**	-	8.40	147.5	4.59	72.1
**3a**	-	7.75	139.0	3.48, 3.82, 3.28, 2.37	72.2, 71.8, 51.9, 47.9
**3b**	-	7.37	137.9	4.20, 4.12	74.5, 61.0
**5**		7.10, 6.47, 6.90, 4.40	172.7, 138.8, 92.4 (2 CH)	3.60, 3.95, 4.28-4.11, 4.41, 4.97 (*coord.*), 6.25, 6.98 (*non coord.*)	81.1, 74.5, 73.2, 56.7, 55.8 (*coord.*) 130.3 (*non coord.*)
**$5H_2$**	-	6.92, 6.83, 4.55, 4.56	175.2, 173.4, 72.6, 72.4	4.76, 4.59, 4.54, 4.11, 4.04, 3.39	74.4, 73.7, 73.3, 71.2, 60.5, 56.9
**6**	-	7.30	141.6	4.10, 3.56	61.8, 54.6
**7**	-	3.17, 2.30	50.8, 46.1	6.45, 6.16	126.8

3. Catalytic dehydrogenation of formaldehyde or paraformaldehyde / water mixtures.

3.1. General method

In a typical experiment, a 25 mL two-neck round-bottom flask was connected to a reflux condenser with argon inlet/outlet which is coupled to a water filled gas burette (see Supplementary Figure1). The second neck of the flask was capped with a septum. The air and moisture were purged with vacuum - argon cycles for 20 min (three times). A degassed solution of formaldehyde (75 μL of a 37 wt% aqueous solution, 1.0 mmol, 1.0 equiv) in H_2O (2 mL) was added followed by the addition of the required amount of base [KOH or $Ba(OH)_2$] and the mixture was heated to 60 °C. After equilibration, the required amount of ruthenium complex in THF (200 μL) was added with a syringe. The mixture was stirred vigorously in an open system and the volume of liberated gas was recorded periodically until gas evolution ceased. Released hydrogen was quantified by recording its volume displacement in the eudiometer and correcting its volume for water content volume (Supplementary Figure1). Under optimized conditions, feeding of substrate was carried out by sequential additions of formaldehyde (1.0 mmol, 1.0 equiv) and KOH (2.0 mmol, 2.0 equiv) in 1 mL of H_2O to the reaction mixture via a syringe and the volume of released H_2 was recorded periodically.

In the experiments with paraformaldehyde, a solution of substrate (30.0 mg, 1.0 mmol, 1.0 equiv) and KOH (337 mg, 6.0 mmol, 6.0 equiv) in 2 mL H_2O was heated to 60 °C. The catalyst dissolved in THF (200 μL) was added and the continuous production of H_2 was monitored. After the gas evolution ceased, the mixture was extracted with Et_2O (3 x 2 mL) and the aqueous phase was analysed by ^{1}H and ^{13}C NMR. The conversion was determined independently by quantification of the amount of K_2CO_3 by elemental analysis of the residue obtained after drying the aqueous phase.

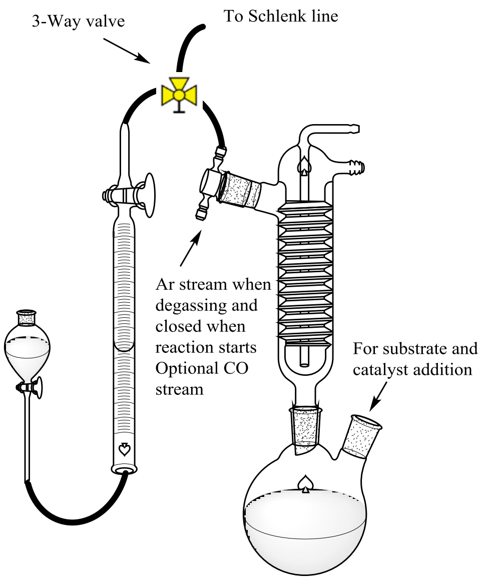

Supplementary Figure 1. Technical set-up for the dehydrogenative coupling (DHC) reaction of HCOH/H_2O in open system. Open system refers to the reaction flask connected to a water burette or the reaction flask open to atmosphere (air).

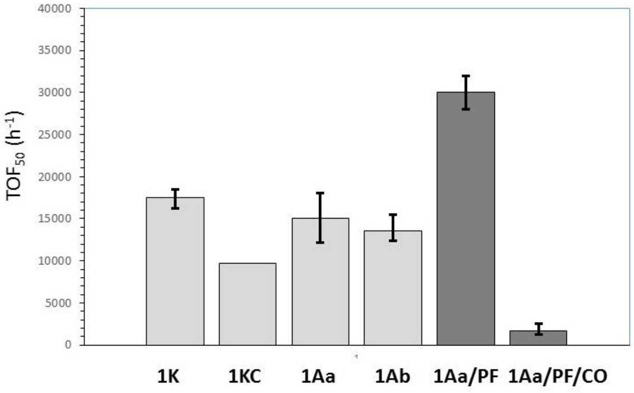

Supplementary Figure 2. Comparison of the catalytic efficiency of complexes **1** in formalin and paraformaldehyde reforming reactions, under argon or carbon monoxide atmosphere. Conditions: c_0(FA) = 0.47 M, 6 equiv KOH in water at 60 °C using catalyst loading of 0.4 mol%.

3.2. Determination of the lifetime of the catalyst

The lifetime of the catalyst **1Aa** in the dehydrogenation of aqueous formaldehyde under catalytic conditions was determined by measuring the total turnover number (TTO). The experiment was started with a 2.2 mL solution (H_2O/THF 10:1) containing 1.8 mM **1Aa**, 2.72 M KOH and 0.47 M formaldehyde at

60 °C. When complete conversion was achieved, 1 mL of a stock solution containing formaldehyde (1M) and KOH (2M) were added sequentially and the reaction was continued until no hydrogen gas evolution was observed.

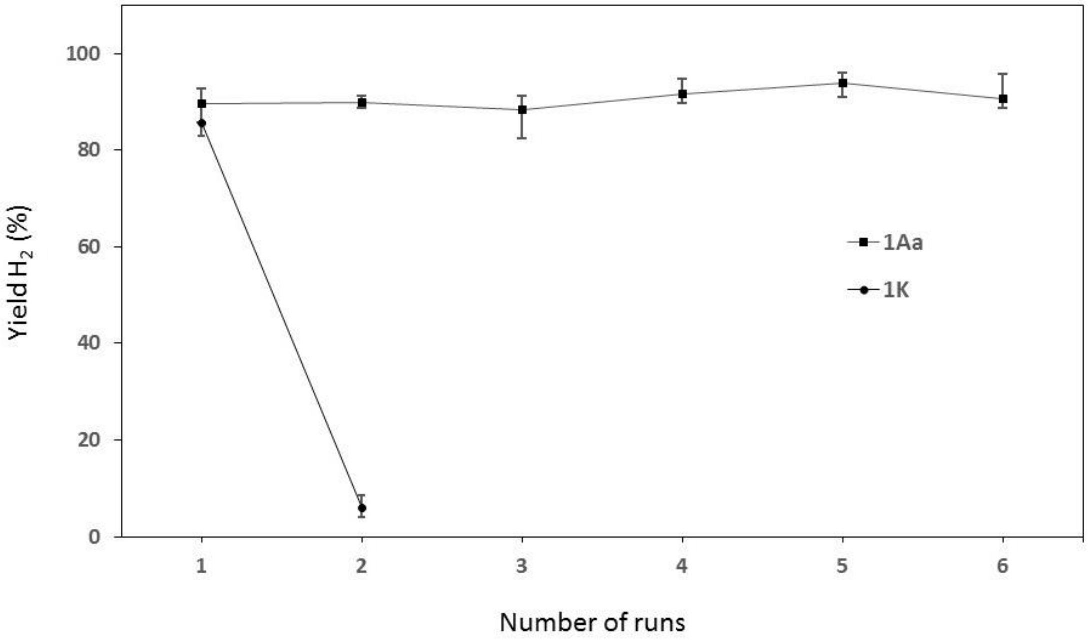

Supplementary Figure 3. Recycling experiments over 6 runs after 20 min (**1Aa**) and over 2 runs after 2h (**1K**). Conditions:

c_0(FA) = 0.47 M in water at 60 °C using a catalyst loading (0.4 mol%)

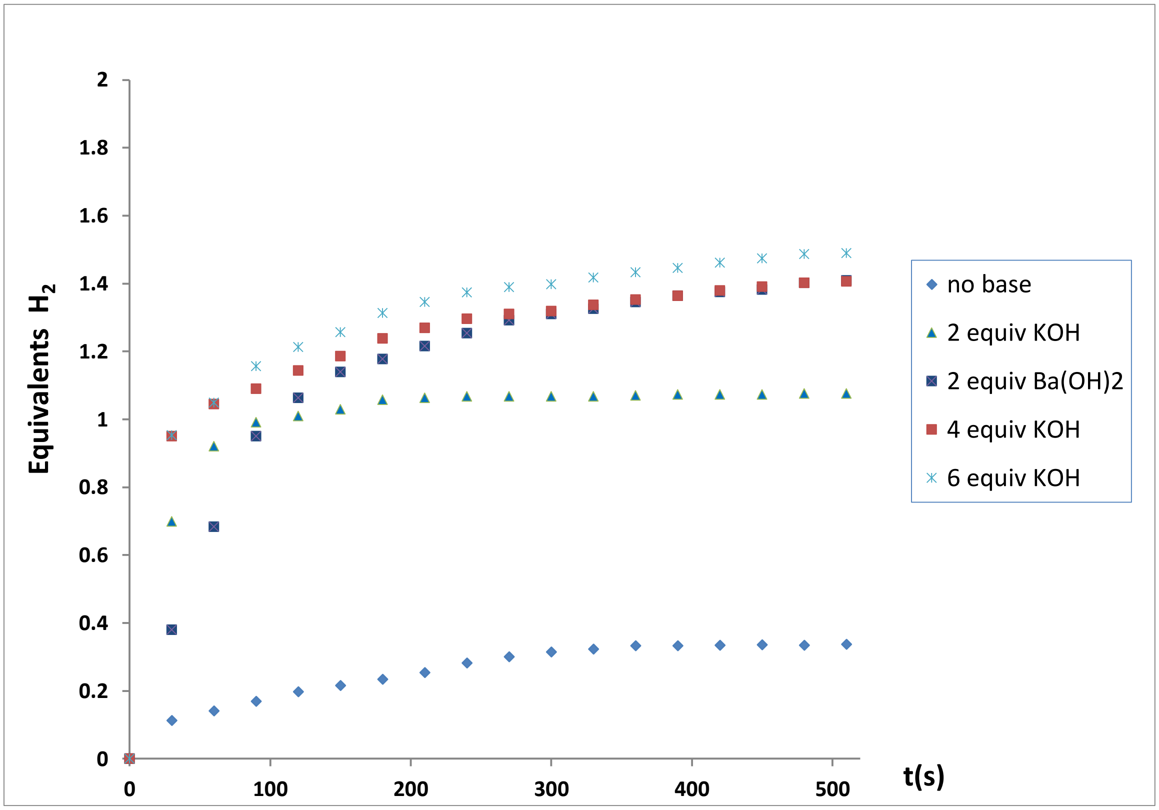

Supplementary Figure 4. Plots of catalytic H_2CO decomposition in H_2O c_0(FA) = 470 mM at 60 °C using complex **1K** (1.81 mM) without base and at different base concentrations [0.91M (KOH), 0.91M ($Ba(OH)_2$), 1.81M (KOH) and 2.73M (KOH)] in the early stage of the reaction.

Supplementary Table 2. Catalytic decomposition of HCOH at different concentrations in water at 60 °C using complex **1Aa** (0.4 mol%) and KOH (6 equiv).

Entry	[HCOH] (M)	TOF_{50}(h^{-1})[a]	Total Yield H_2(%)[b]	TON_{max}/duration[c]
1	0.5	15101	90	450/12min
2	0.75	7500	89.5	447/2h
3	1.25	1875	73	350/12h
4	2.5	-	32	160/12h
5	5	-	20	100/12h

[a]TOF values after 50% conversion (1 equiv H_2 released per formaldehyde molecule). [b]Yield considering 2 equiv H_2/equiv HCOH. [c] TON = mmol H_2 released / mmol [Ru].

4. NMR Spectra

Complex 1Aa

a)

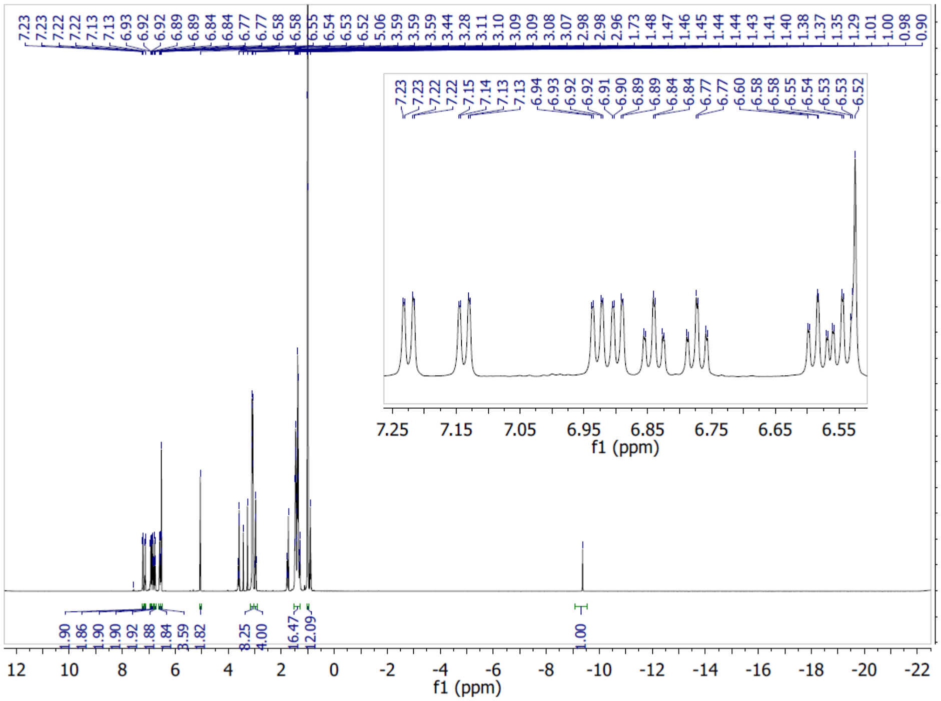

b)

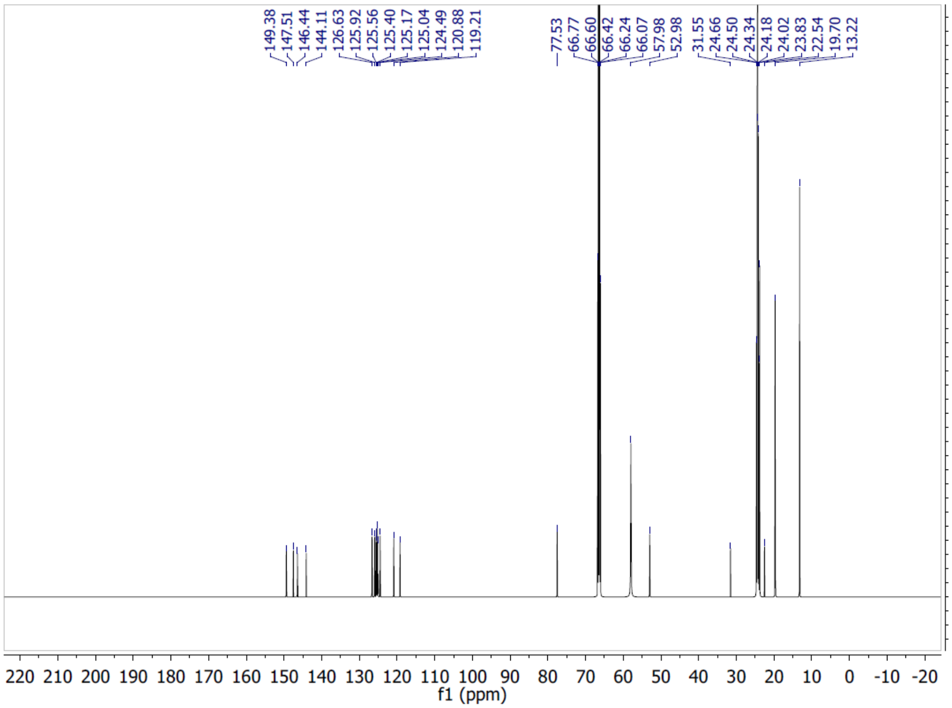

Supplementary Figure 5. a) ^{1}H NMR (500 MHz, d_8-THF, 298K) and b) $^{13}C\{1H\}$ NMR (126 MHz, d_8-THF, 298K) spectra of complex **1Aa**.

Complex 1Ab

a)

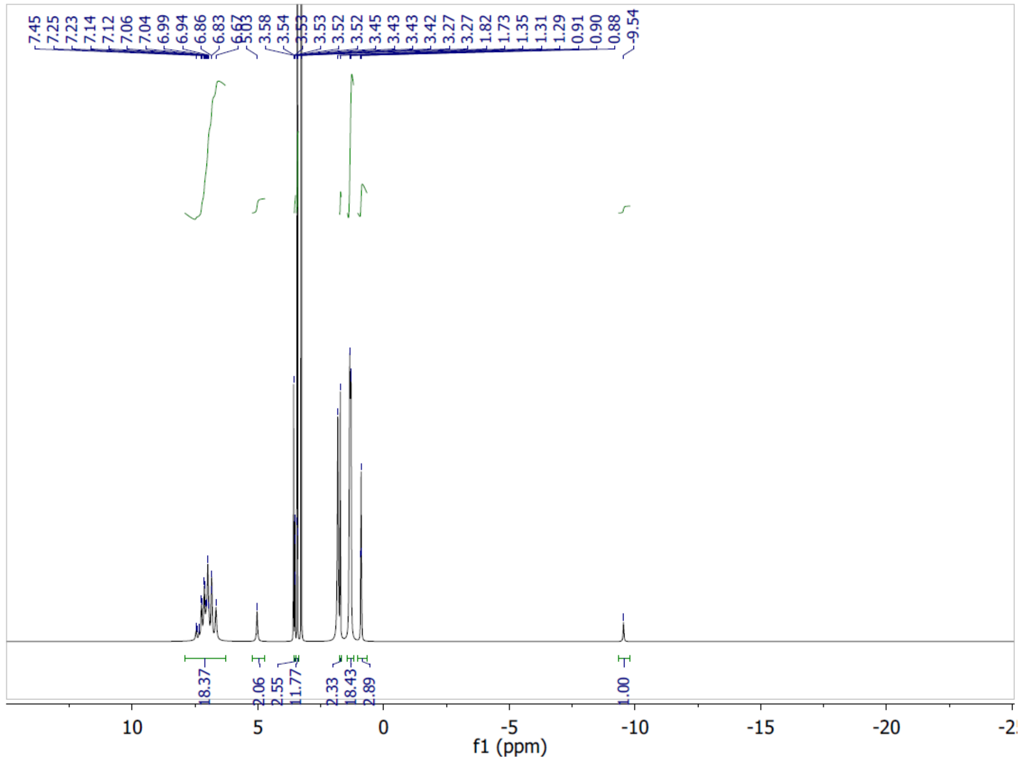

b)

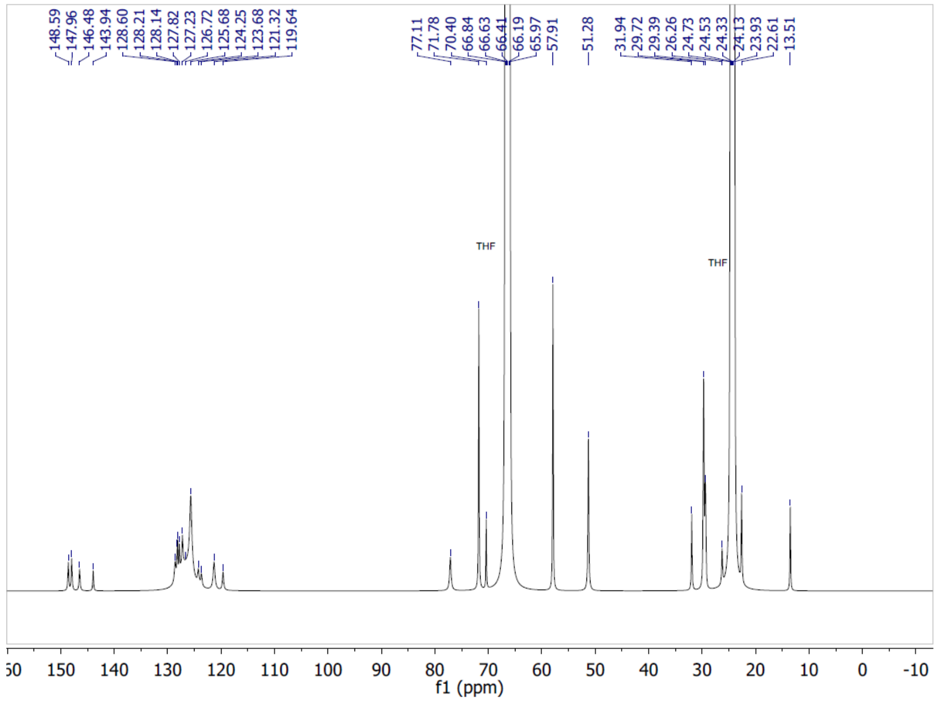

Supplementary Figure 6. a) ^{1}H NMR (400 MHz, d_8-THF, 298K) and b) ^{13}C{1H} NMR (100 MHz, d_8-THF, 298K) spectra of complex **1Ab**.

Complex 1KC

a)

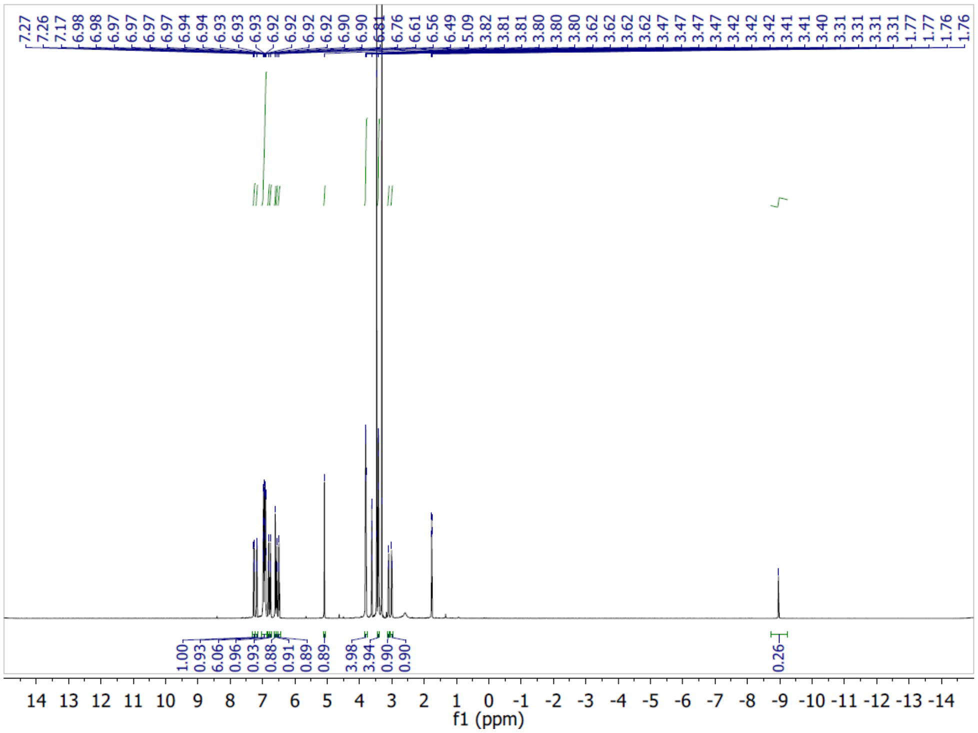

b)

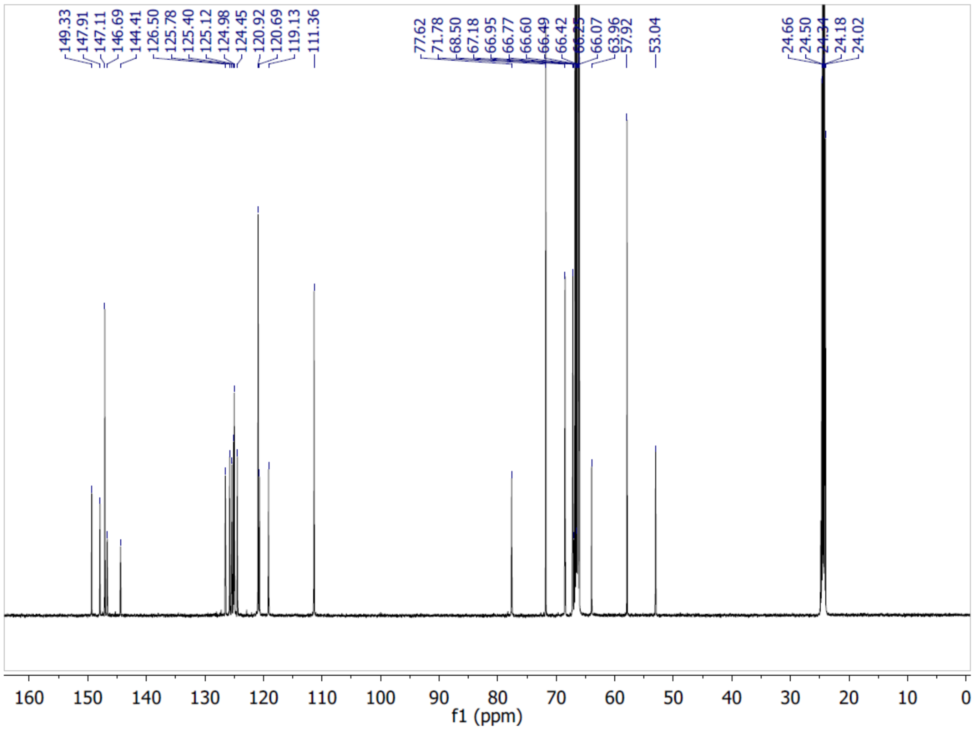

Supplementary Figure 7. a) ^{1}H NMR (500 MHz, d_8-THF, 298K) and b) ^{13}C{1H} NMR (126 MHz, d_8-THF, 298K) spectra of complex **1KC**.

Complex 3b

a)

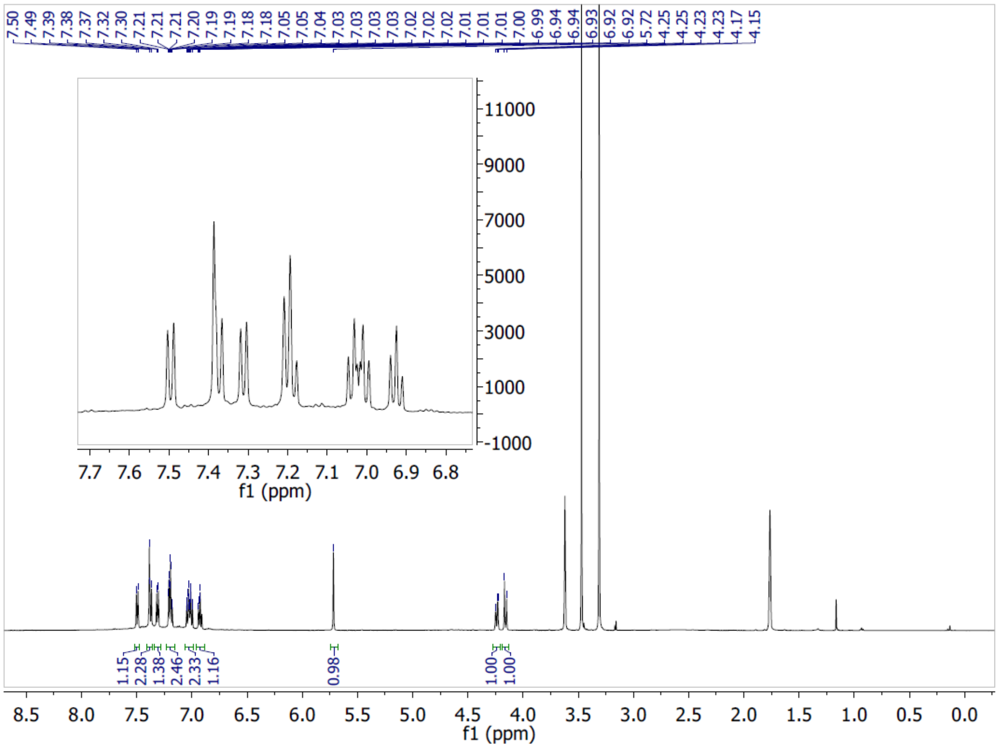

b)

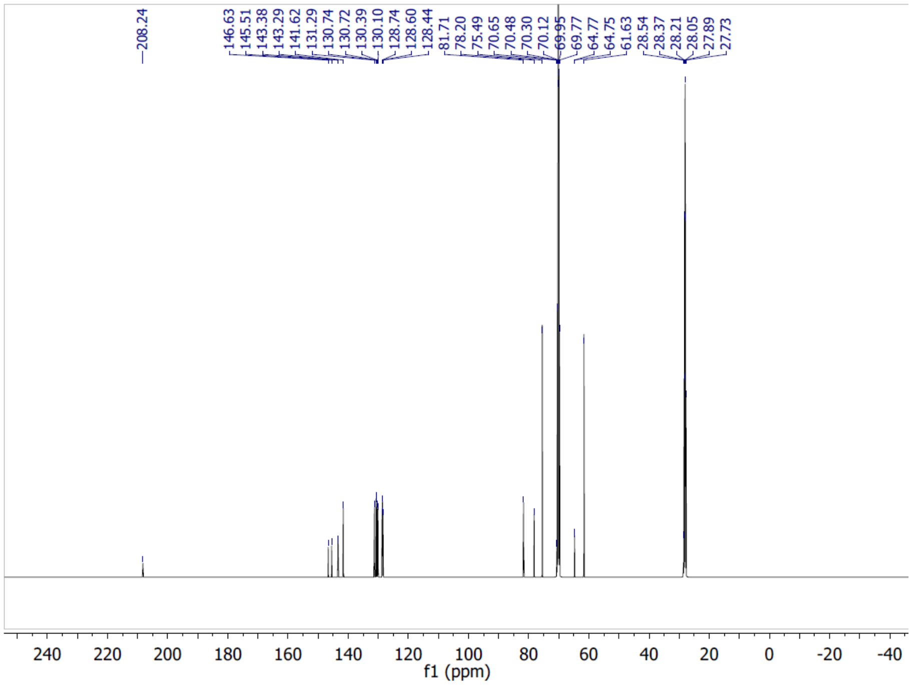

Supplementary Figure 8. a) ^{1}H NMR (500 MHz, d_8-THF, 298K) and b) $^{13}C\{^{1}H\}$ NMR (126 MHz, d_8-THF, 298K) spectra of complex **3b**.

Complex 5

a)

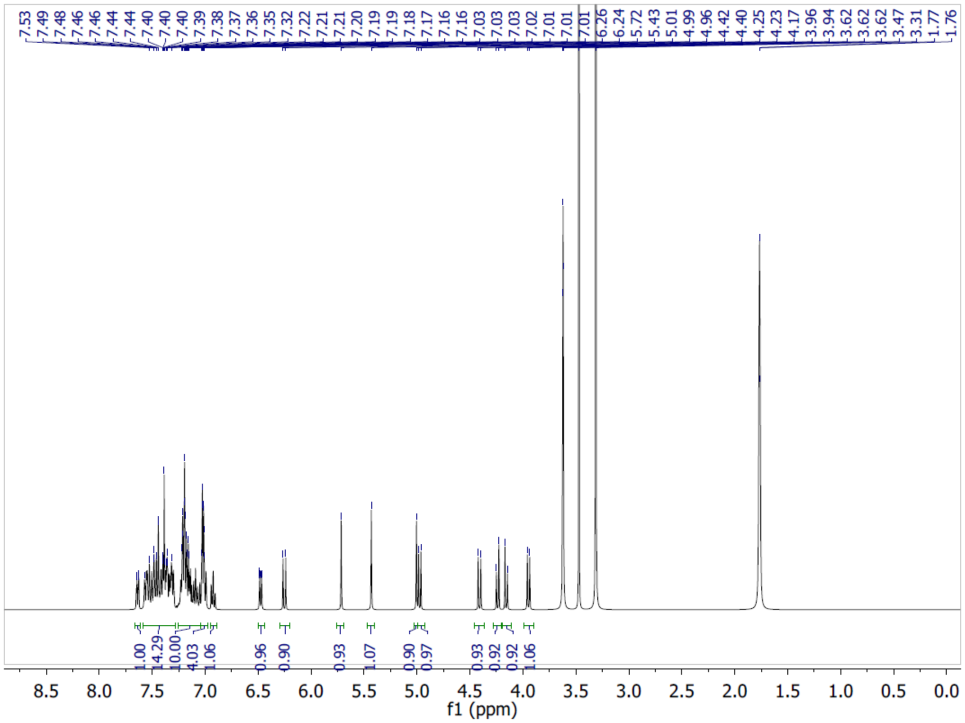

b)

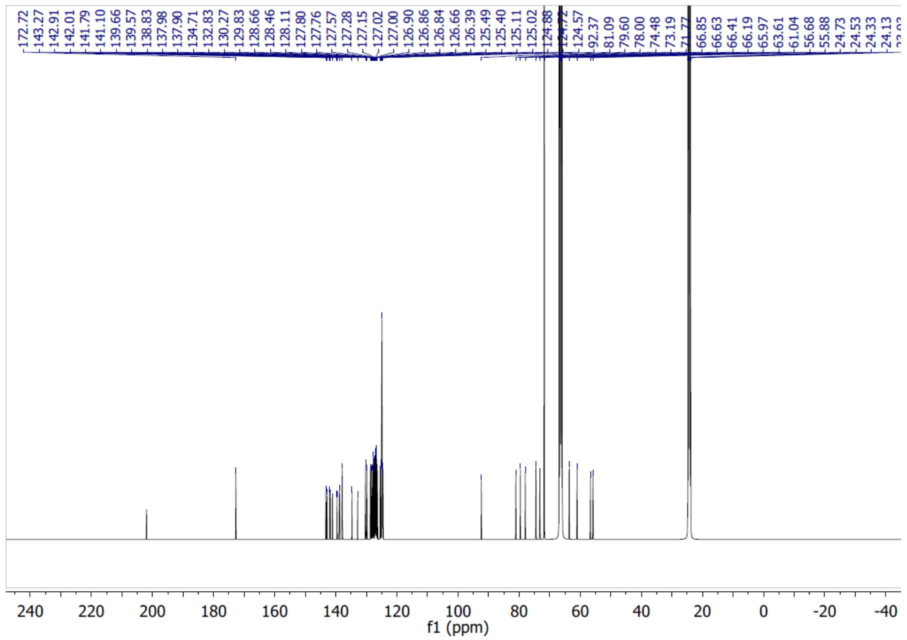

Supplementary Figure 9. a) ^{1}H NMR (400 MHz, d_8-THF, 298K) and b) ^{13}C{1H} NMR (100 MHz, d_8-THF, 298K) spectra of complex **5**.

Complex 5H2

a)

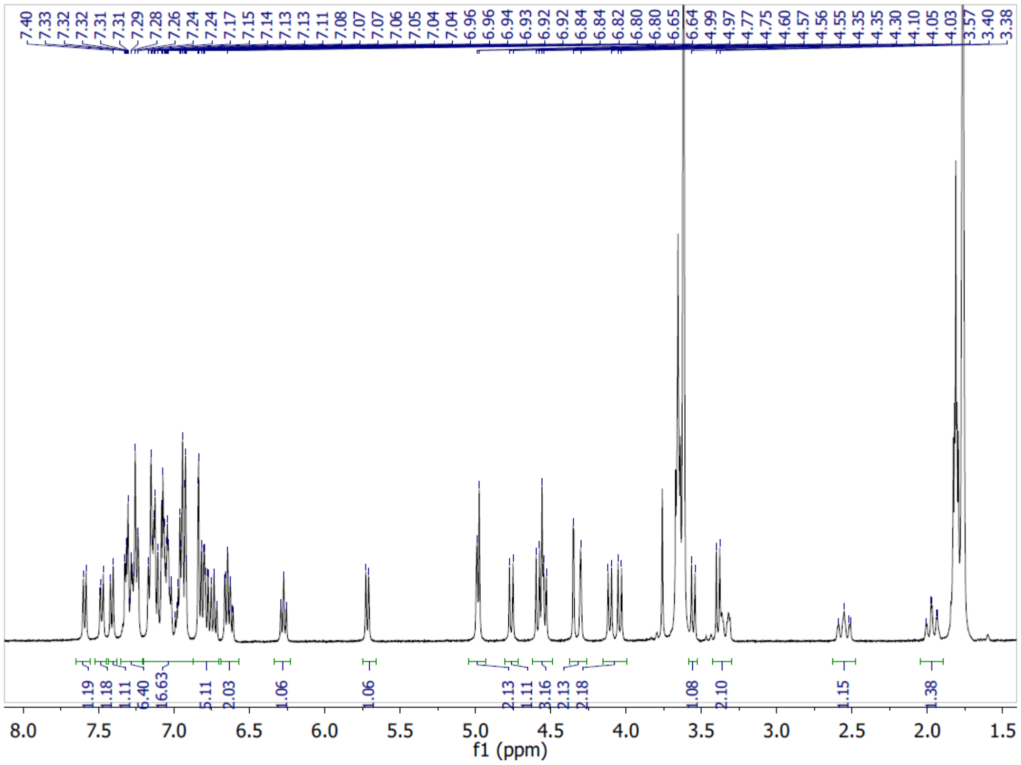

b)

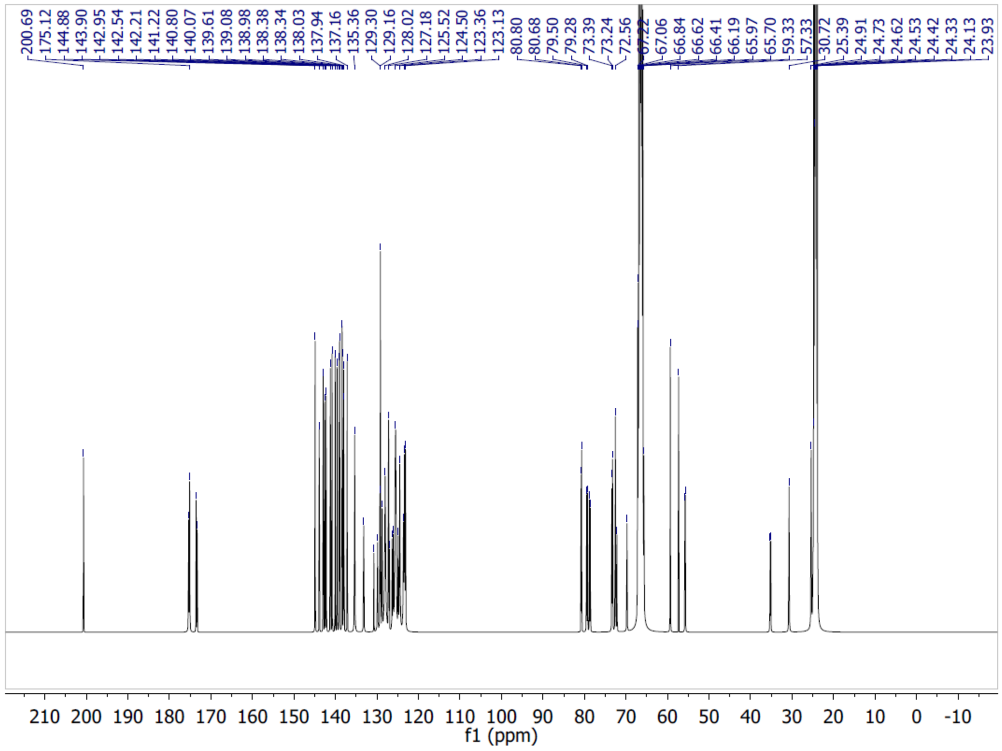

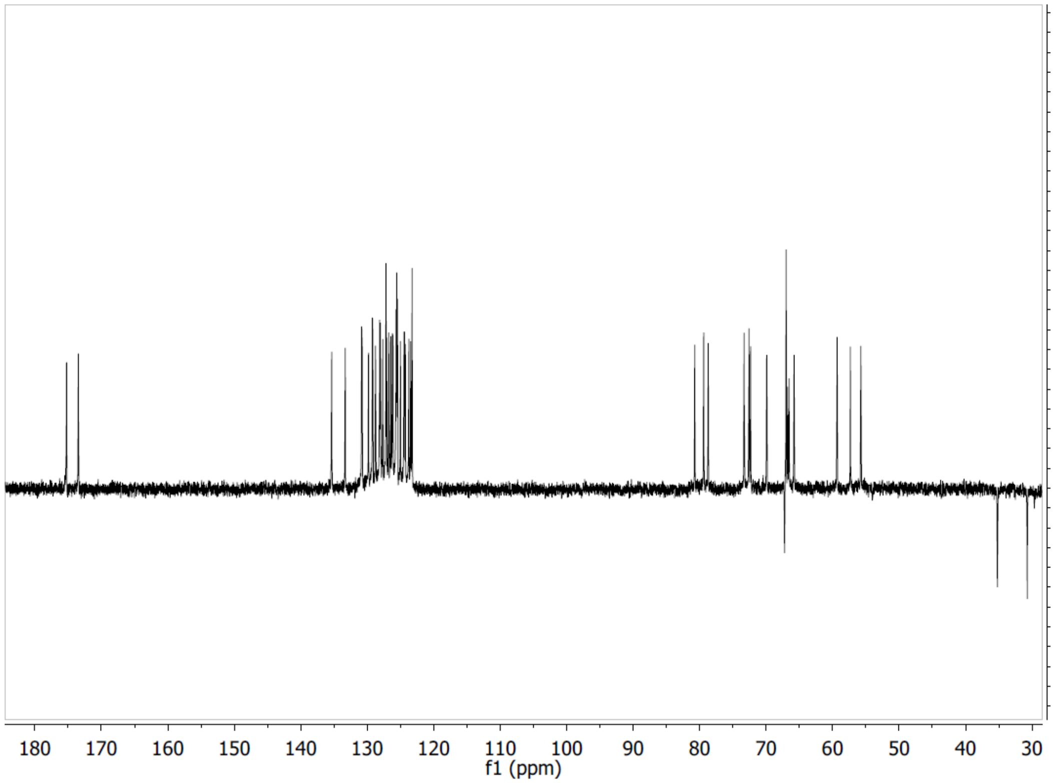

Supplementary Figure 10. a) ^{1}H NMR (400 MHz, d_8-THF, 298K) b) $^{13}C\{1H\}$ and 135 DEPT NMR (100 MHz, d_8-THF, 298K) spectra of complex **5H2**.

Complex 6

a)

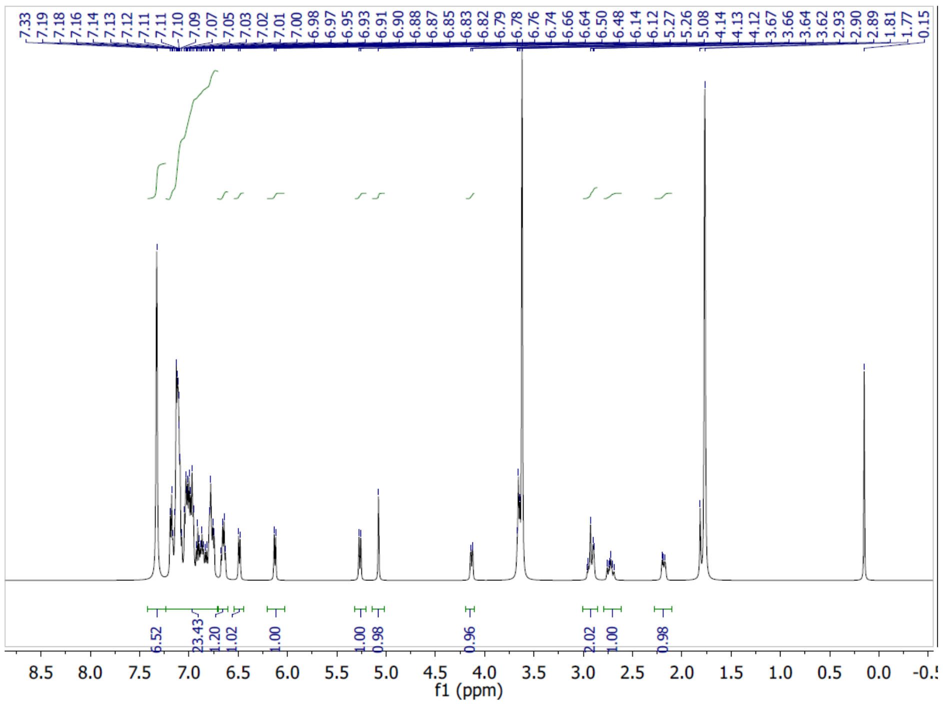

b)

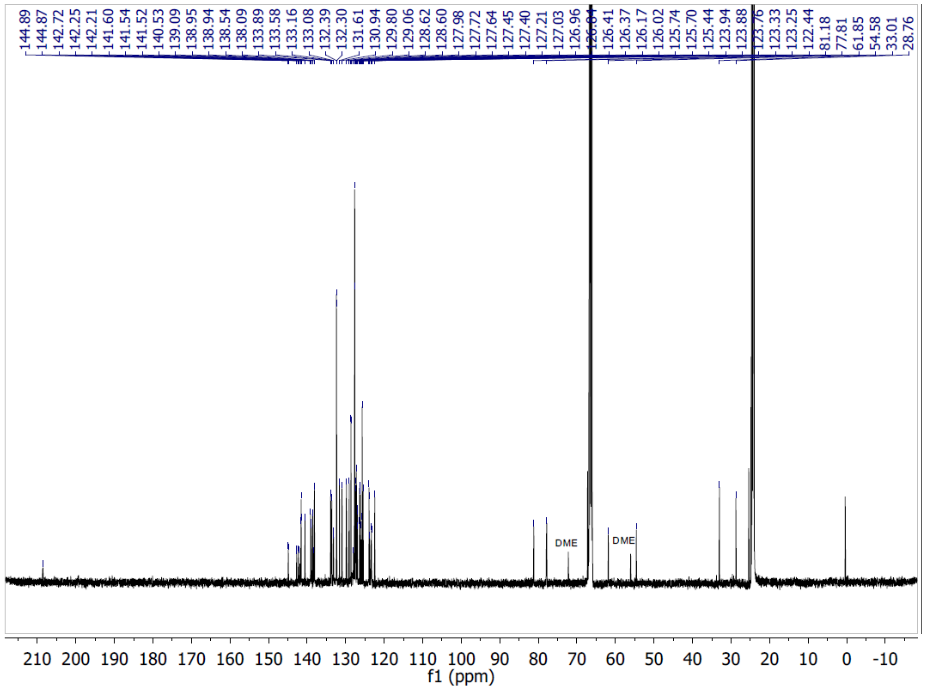

Supplementary Figure 11. a) ^{1}H NMR (500 MHz, d_8-THF, 298K) and b) ^{13}C{1H} NMR (126 MHz, d_8-THF, 298K) spectra of complex **6**.

Complex 7.

a)

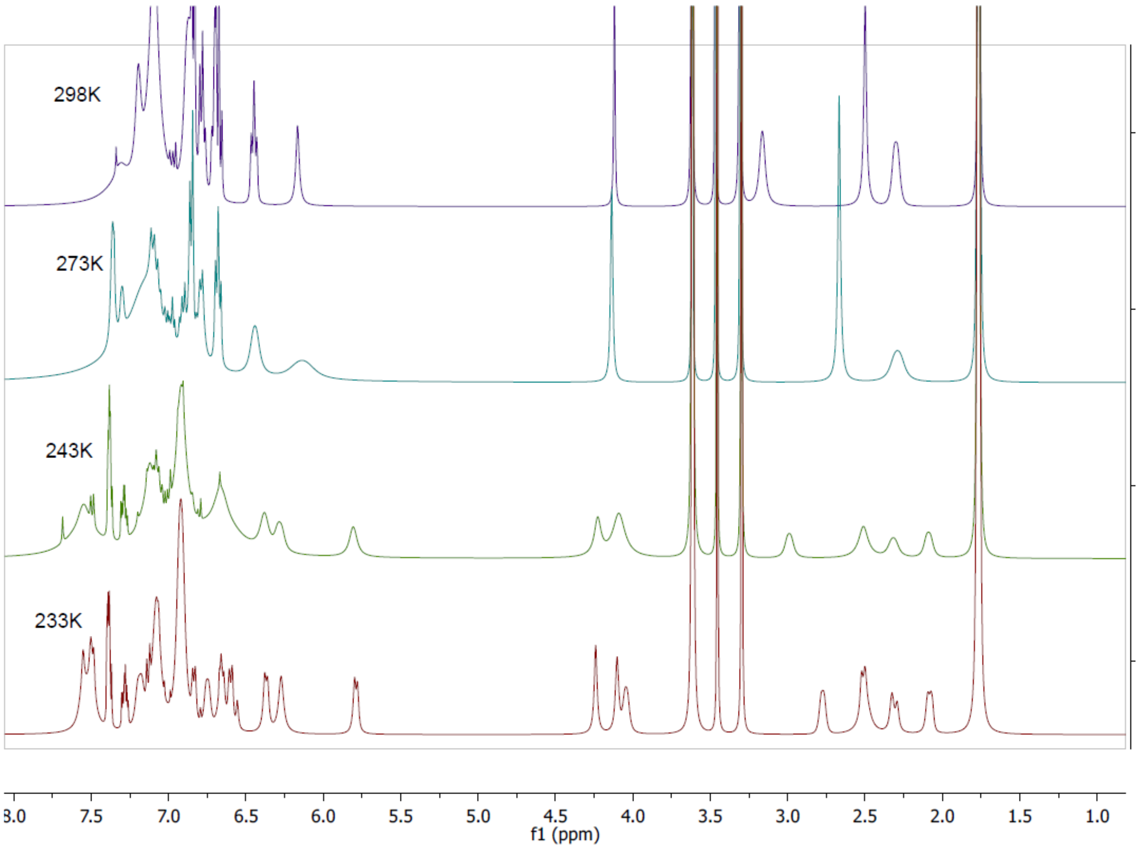

298K
273K
243K
233K
8.0
7.5
7.0
6.5
6.0
5.5
5.0
4.5
4.0
3.5
3.0
2.5
2.0
1.5
1.0
f1 (ppm)

b)

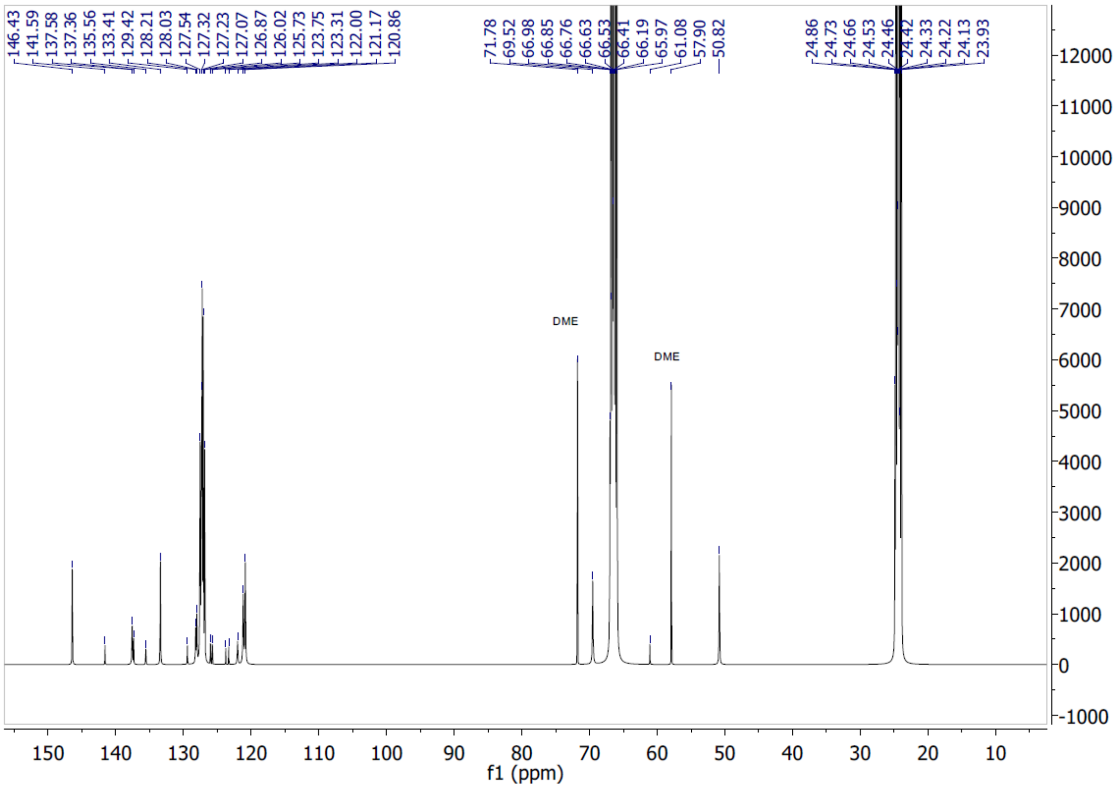

Supplementary Figure 12. a) ^{1}H NMR (400 MHz, d_8-THF, 298-233K) and b) ^{13}C{1H} NMR (100 MHz, d_8-THF, 298K) spectra of complex **7**.

5. Selected GC chromatograms

a)

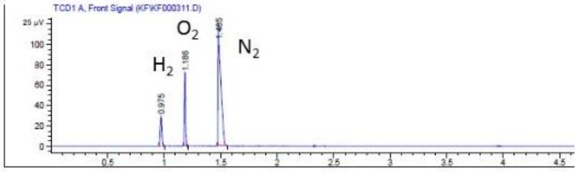


```
=====================================================================
                      Area Percent Report
=====================================================================

Sorted By             :      Signal
Multiplier:                  :      1.0000
Dilution:                    :      1.0000
Sample Amount:               :    200.00000  [ng/ul]   (not used in calc.)
Use Multiplier & Dilution Factor with ISTDs


Signal 1: FID2 B, Back Signal


Signal 2: TCD1 A, Front Signal

Peak RetTime Type  Width     Area      Height     Area
  #   [min]        [min]  [25 µV*s]   [25 µV]      %
----|-------|----|-------|----------|----------|--------|
   1   0.975 BB    0.0169   29.84718   28.79872 10.56950
   2   1.186 BB    0.0123   55.62575   71.43279 19.69822
   3   1.485 BB    0.0258  196.91675  105.48404 69.73227

         Totals :                  282.38968  205.71554
```

b)

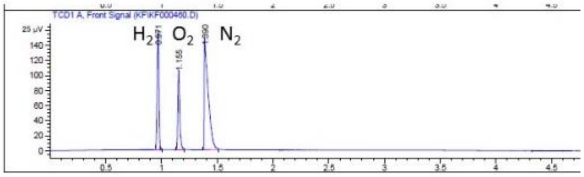


```
=====================================================================
                      Area Percent Report
=====================================================================

Sorted By             :      Signal
Multiplier:                  :      1.0000
Dilution:                    :      1.0000
Sample Amount:               :    500.00000  [ng/ul]   (not used in calc.)
Use Multiplier & Dilution Factor with ISTDs


Signal 1: FID2 B, Back Signal


Signal 2: TCD1 A, Front Signal

Peak RetTime Type  Width     Area      Height     Area
  #   [min]        [min]  [25 µV*s]   [25 µV]      %
----|-------|----|-------|----------|----------|--------|
   1   0.971 BB    0.0176  168.64563  153.68796 25.47600
   2   1.155 BB    0.0189  123.62164  102.23316 18.67457
   3   1.390 BB    0.0345  369.71109  141.31990 55.84942

Totals :                   661.97836  397.24102
```

c)

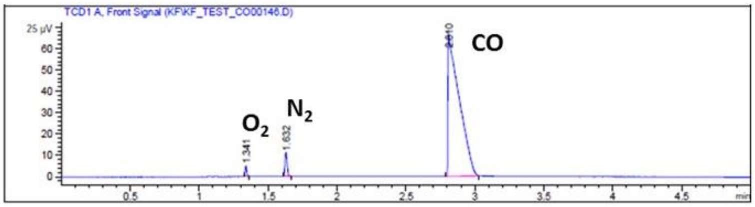


```
=====================================================================
                      Area Percent Report
=====================================================================

Sorted By             :      Signal
Multiplier:                  :      1.0000
Dilution:                    :      1.0000
Sample Amount:               :    200.00000  [ng/ul]   (not used in calc.)
Use Multiplier & Dilution Factor with ISTDs

Signal 2: TCD1 A, Front Signal

Peak RetTime Type  Width     Area      Height     Area
  #   [min]        [min]  [25 µV*s]   [25 µV]      %
----|-------|----|-------|----------|----------|--------|
   1   1.341 BB    0.0133    4.25224    4.90659  1.07877
   2   1.632 BB    0.0179   12.17211   10.84489  3.08800
   3   2.810 BB    0.0712  377.74966   64.08175 95.83322
```

Supplementary Figure 13. a) Gas chromatogram (GC-TCD) of injection of gas phase of dehydrogenation of formaldehyde aqueous solution catalysed by **1Aa**. b) Chromatogram of injection of the reaction of paraformaldehyde decomposition catalysed by complex **1Aa**. c) Chromatogram of injection of 20 µL of CO.

6. Crystallographic data

X-ray diffraction data were collected on a Bruker SMART Apex II diffractometer equipped with CCD area detector. Mo-Kα radiation (0.71073 Å) was used at T = 100 K. The refinement against full matrix (versus F2) was performed with SHELXTL (ver. 6.12) and SHELXL-97. Empirical absorption correction was performed with SADABS (ver. 2.03). All non-hydrogen atoms were refined anisotropically where not noted otherwise. The contribution of the hydrogen atoms, in their calculated positions, was included in the refinement using a riding model. The X-ray crystallographic coordinates for structures reported in this article have been deposited at the Cambridge Crystallographic Data Centre (CCDC), under deposition number CCDC-1502942 (**1Aa**), 1502945 (**1Ab**), 1502948 (**5**), 1502951 (**5H_2**), 1502952 (**6**) and 1502953 (**7**). These data can be obtained free of charge from the Cambridge Crystallographic Data Centre via www.ccdc.cam.ac.uk/data_request/cif.

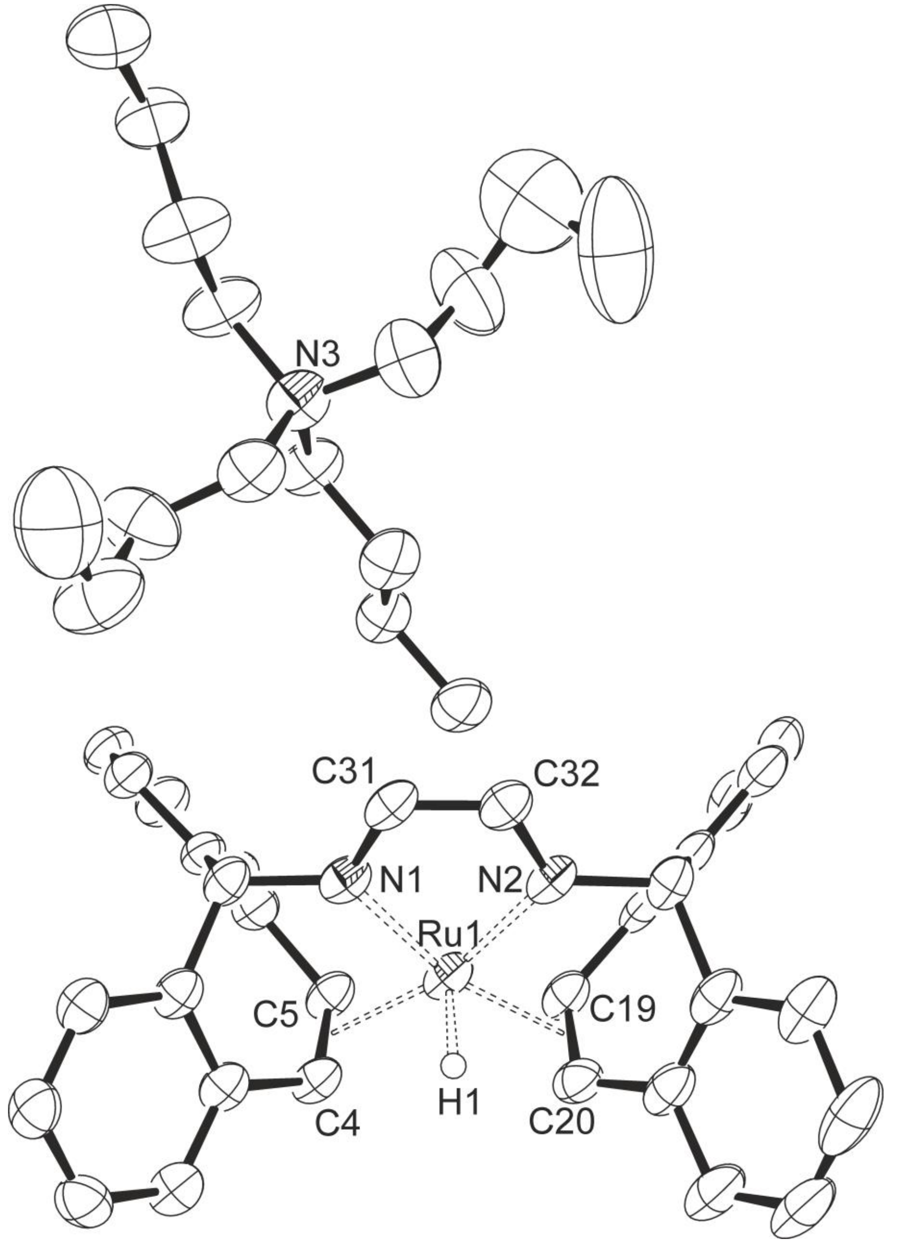

Supplementary Figure 14. ORTEP plot of [Ru(H)(trop$_2$DAD)][NBu$_4$]]x[NBu$_4$]Br (**1Aa×NBu$_4$Br**). Ellipsoids are shown at 50% probability. Co-crystallised [N(Bu$_4$)Br] and hydrogen atoms except hydride H1 are omitted for clarity. Selected bond lengths [Å] and angles [°]: Ru1-ct1 2.027(4), Ru1-ct2 2.025(3), C4-C5 1.435(6), C19-C20 1.443(6), Ru1-N1 1.965(3), Ru1-N2 1.959(3), N1-C31 1.352(5), N2-C32 1.361(5), C31-C32 1.371(4), ct1-Ru1-ct2 100.59(2), ct1-Ru1-N1 89.15(2), ct2-Ru1-N2 89.20(2), N1-Ru1-N2 79.4(1).

SupplementaryTable 3. Crystal data and structure refinement for **1Aa** x[NBu_4]Br.

Empirical formula	$C_{64}H_{97}Br_{0.75}N_4Ru$
Formula weight	1083.45
Temperature/K	99.35
Crystal system	triclinic
Space group	P-1
a/Å	9.8805(14)
b/Å	16.485(2)
c/Å	20.220(3)
α/°	73.577(2)
β/°	77.564(2)
γ/°	85.951(2)
Volume/Å^3	3084.7(8)
Z	2
ρ_{calc}mg/mm^3	1.166
m/mm^{-1}	0.779
F(000)	1158.0
Crystal size/mm^3	0.18 × 0.1 × 0.08
Radiation	Mo Kα (λ = 0.71073)
2Θ range for data collection	2.58 to 52.68°
Index ranges	-12 ≤ h ≤ 12, -20 ≤ k ≤ 20, -25 ≤ l ≤ 25
Reflections collected	38016
Independent reflections	12463 [R_{int} = 0.0730, R_{sigma} = 0.0943]
Data/restraints/parameters	12463/0/675
Goodness-of-fit on F^2	1.026
Final R indexes [I>=2σ (I)]	R_1 = 0.0605, wR_2 = 0.1349
Final R indexes [all data]	R_1 = 0.1022, wR_2 = 0.1537
Largest diff. peak/hole / e Å^{-3}	0.91/-0.48

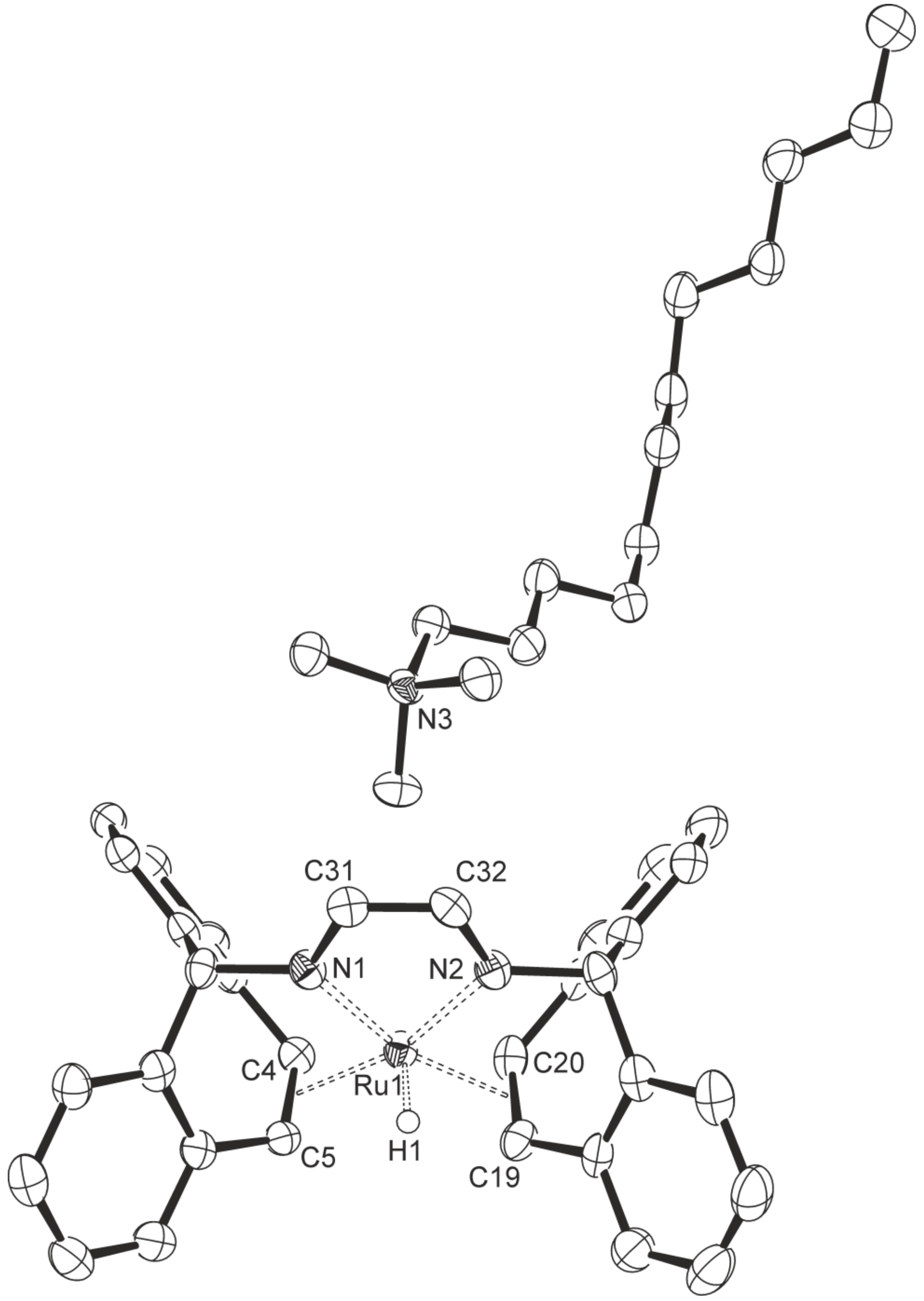

Supplementary Figure 15. ORTEP plot of [Ru(H)(trop$_2$dad)][NMe$_3$(dodecyl)]] (**1Ab**). Ellipsoids are shown at 50% probability. Hydrogen atoms except hydride H1 are omitted for clarity. Selected bond lengths [Å] and angles [°]: Ru1-ct1 2.031(4), Ru1-ct2 2.047(4), C4-C5 1.448(4), C19-C20 1.428(6), Ru1-N1 1.977(3), Ru1-N2 1.976(3), N1-C31 1.360(5), N2-C32 1.364(5), C31-C32 1.371(6), Ru1-H1 1.19(3), ct1-Ru1-ct2 101.0(2), ct1-Ru1-N1 89.42(1), ct2-Ru1-N2 88.71(1), N1-Ru1-N2 79.1(1), N1-Ru1-H1 102(2), N2-Ru1-H1 95(2).

Supplementary Table 4. Crystal data and structure refinement for **1Ab**.

Empirical formula	$C_{47}H_{59}N_3Ru$
Formula weight	767.04
Temperature/K	103.0
Crystal system	monoclinic
Space group	$P2_1/c$
a/Å	9.3661(3)
b/Å	14.9881(5)
c/Å	30.4421(10)
α/°	90
β/°	96.552(2)
γ/°	90
Volume/Å^3	4245.6(2)
Z	4
ρ_{calc}mg/mm^3	1.200
m/mm^{-1}	3.230
F(000)	1624.0
Crystal size/mm^3	0.16 × 0.12 × 0.1
Radiation	CuKα (λ = 1.54178)
2Θ range for data collection	5.844 to 130.17°
Index ranges	-10 ≤ h ≤ 11, -17 ≤ k ≤ 8, -35 ≤ l ≤ 35
Reflections collected	17205
Independent reflections	7031 [R_{int} = 0.0598, R_{sigma} = 0.0697]
Data/restraints/parameters	7031/0/468
Goodness-of-fit on F^2	1.023
Final R indexes [I>=2σ(I)]	R_1 = 0.0489, wR_2 = 0.1190
Final R indexes [all data]	R_1 = 0.0673, wR_2 = 0.1260
Largest diff. peak/hole / e Å^{-3}	0.82/-0.48

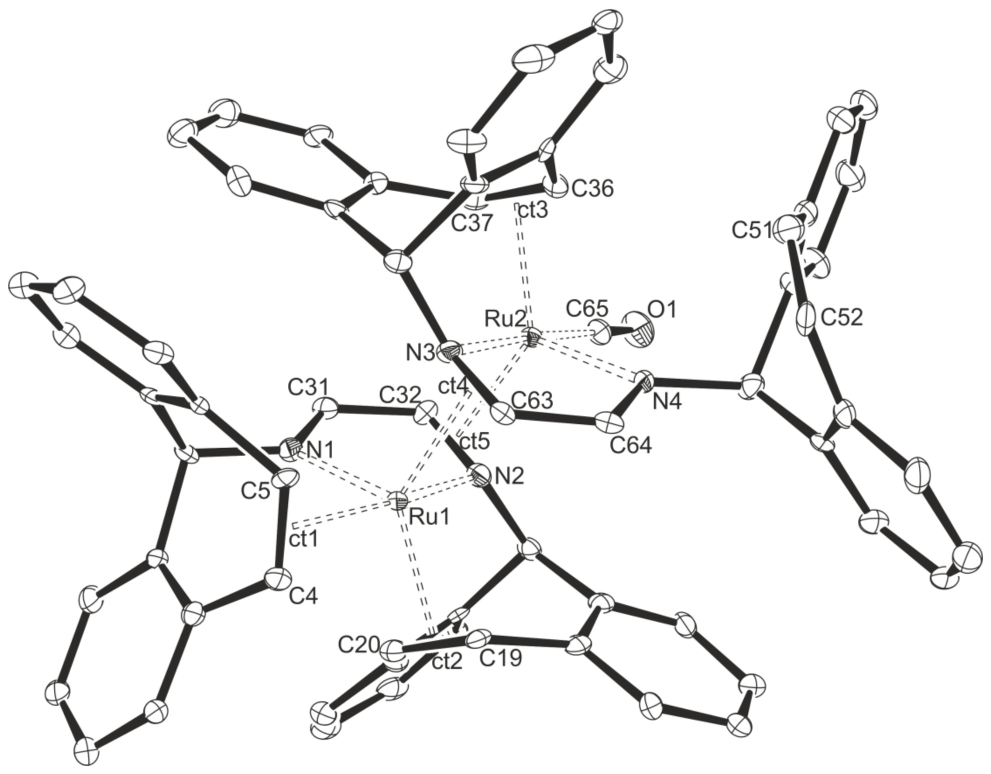

Supplementary Figure 16. ORTEP plot [Ru(trop$_2$dad)Ru(trop$_2$dad)(CO)].(thf) (**5**). Ellipsoids are shown at 50% probability. Solvent molecule and hydrogen atoms are omitted for clarity. Selected bond lengths (Å) and angles (°): Ru1-N1 2.051(6), Ru1-N2 2.107(7), Ru1-ct1 2.108(6), Ru1-ct2 2.056(6), Ru1-ct4 2.038(5), Ru2-N3 2.095(7), Ru2-N4 2.144(6), Ru2-ct3 2.040(6), Ru2-ct5 1.985(5), Ru2-C65 1.834(8), N1-C31 1.293(12), N2-C32 1.446(10), C31-C32 1.459(10), N3-C63 1.425(10), N4-C64 1.280(11), C63-C64 1.436(9), C4-C5 1.418(12), C19-C20 1.439(10), C36-C37 1.430(11), C51-C52 1.358(13), ct1-Ru1-ct2 96.77(3), ct1-Ru1-N1 86.93(3), ct2-Ru1-N2 84.26(3), N1-Ru1-N2 81.2(2), ct3-Ru2-C65 92.68(3), ct3-Ru2-N3 90.31(3), C65-Ru2-N4 99.8(3), C3-Ru2-N4 78.9(2).

Supplementary Table 5. Crystal data and structure refinement for complex **5**.thf

Empirical formula	$C_{69}H_{56}N_4O_2Ru_2$
Formula weight	1175.31
Temperature/K	100.0
Crystal system	triclinic
Space group	P-1
a/Å	9.9364(15)
b/Å	13.405(2)
c/Å	20.051(3)
α/°	95.130(4)
β/°	91.230(4)
γ/°	109.918(4)
Volume/Å^3	2497.0(7)
Z	2
ρ_{calc}mg/mm^3	1.563
m/mm^{-1}	0.661
F(000)	1204.0
Crystal size/mm^3	0.25 × 0.11 × 0.07
Radiation	Mo-Kα (λ = 0.71073)
2Θ range for data collection	2.042 to 56.63°
Index ranges	-13 ≤ h ≤ 13, -17 ≤ k ≤ 17, -26 ≤ l ≤ 26
Reflections collected	27637
Independent reflections	12239 [R_{int} = 0.1171, R_{sigma} = 0.1825]
Data/restraints/parameters	12239/825/694
Goodness-of-fit on F^2	0.920
Final R indexes [I>=2σ(I)]	R_1 = 0.0780, wR_2 = 0.1674
Final R indexes [all data]	R_1 = 0.1469, wR_2 = 0.2066
Largest diff. peak/hole / e Å^{-3}	1.17/-1.46

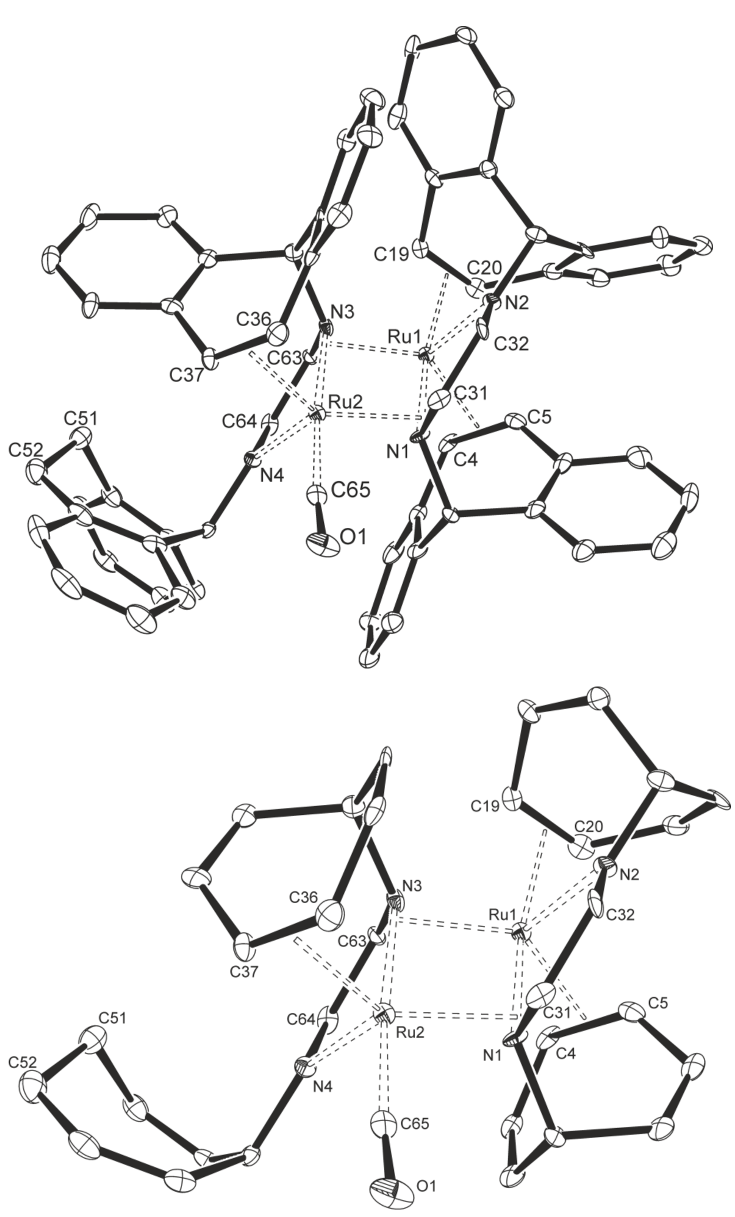

Supplementary Figure 17. ORTEP plot of [Ru(trop$_2$dad)Ru(trop$_2$dad^H)(CO)].(thf) (**5H$_2$**). Ellipsoids are shown at 50% probability. Solvent molecule and hydrogen atoms are omitted for clarity. Selected bond lengths [Å] and angles [°]: Ru1-ct1 2.067(3), Ru1-ct2 2.109(4), Ru2-ct3 2.046(3), C4-C5 1.438(9), C19-C20 1.399(5), C36-C37 1.452(9), C51-C52 1.528(8), Ru1-ct3 2.022(3), Ru1-N1 2.099(7), Ru1-N2 2.058(6), N1-C31 1.430(9), N2-C32 1.321(10), C31-C32 1.441(10), Ru2-ct4 1.988(4), Ru2-N3 2.110(7), Ru2-N4 2.175(6), N3-C63 1.429(9), N4-C64 1.268(10), C63-C64 1.449(10), Ru2-C65 1.862(6), C65-O1 1.146(7), ct1-Ru1-ct2 97.82(2), ct1-Ru1-ct3 132.28(2), ct2-Ru1-ct3 102.99(1), ct1-Ru1-N1 85.10(14), ct2-Ru1-N2 86.65(13), ct3-Ru1-N1 83.35(14), ct3-Ru1-N2 111.39(14), N1-Ru1-N2 81.26(19), ct4-Ru2-ct5 134.63(15), ct4-Ru2-N3 90.79(14), ct5-Ru2-N3 81.94(14), ct5-Ru2-N4 114.14(13), ct4-Ru2-C65 92.03(14), ct5-Ru2-C65 93.86(14), N4-Ru2-C65 102.86(18), N3-Ru2-N4 79.19(18).

Supplementary Table 6. Crystal data and structure refinement for **$5H_2$**.thf

Empirical formula	$C_{69}H_{58}N_4O_2Ru_2$
Formula weight	1177.33
Temperature/K	103.8
Crystal system	triclinic
Space group	P1
a/Å	9.8130(7)
b/Å	11.4771(8)
c/Å	14.3617(10)
α/°	107.245(2)
β/°	98.058(2)
γ/°	111.458(2)
Volume/Å^3	1380.31(17)
Z	1
ρ_{calc}g/cm^3	1.416
μ/mm^{-1}	0.598
F(000)	604.0
Crystal size/mm^3	0.2 × 0.18 × 0.18
Radiation	MoKα (λ = 0.71073)
2Θ range for data collection/°	4.646 to 58.27
Index ranges	-13 ≤ h ≤ 13, -15 ≤ k ≤ 15, -19 ≤ l ≤ 19
Reflections collected	58780
Independent reflections	14330 [R_{int} = 0.0635, R_{sigma} = 0.0646]
Data/restraints/parameters	14330/3/688
Goodness-of-fit on F^2	1.054
Final R indexes [I>=2σ(I)]	R_1 = 0.0379, wR_2 = 0.0664
Final R indexes [all data]	R_1 = 0.0504, wR_2 = 0.0693
Largest diff. peak/hole / e Å^{-3}	0.89/-0.84

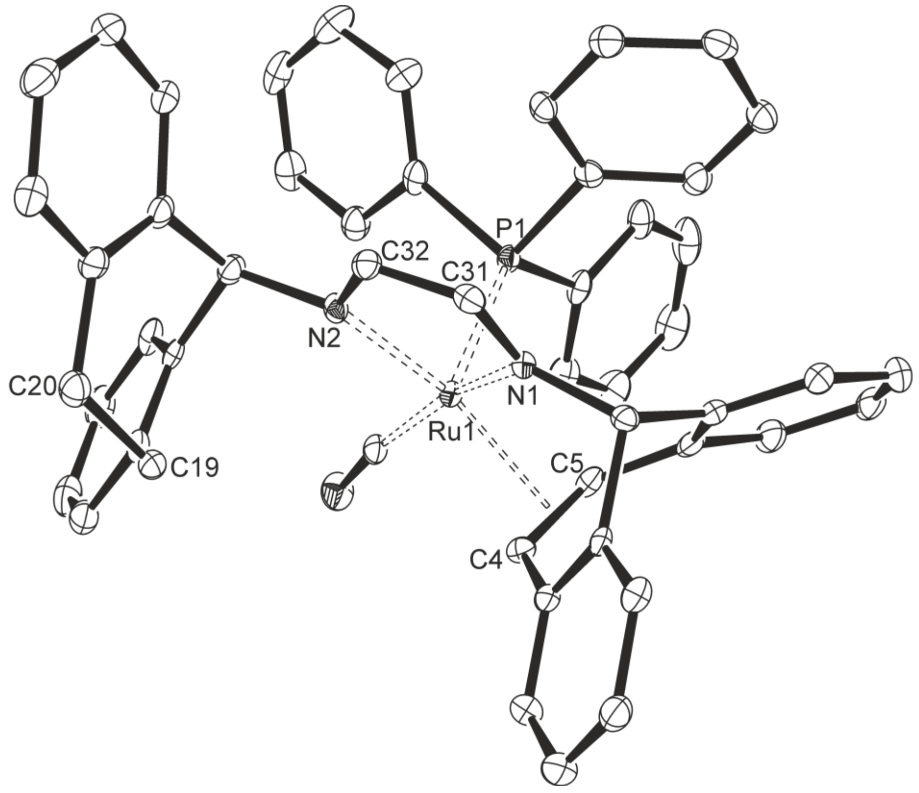

Supplementary Figure 18. ORTEP plot of [Ru(trop$_2$dad)(PPh$_3$)(CO)] (**6**). Ellipsoids are shown at 50% probability. Hydrogen atoms are omitted for clarity. Selected bond lengths (Å) and angles (°): Ru1-N1 2.038(3), Ru1-N2 2.059(3), Ru1-ct1 2.068(2), Ru1-P1 2.329(1), Ru1-C33 1.857(3), N1-C31 1.320(5), N2-C32 1.339(5), C31-C32 1.388(5), C4-C5 1.445(5), C19-C20 1.522(5), C33-O1 1.166(4), N1-Ru1-N2 77.7(1), N1-Ru1-C33 163.8(1), N1-Ru1-P1 102.00(8), P1-Ru1-C33 94.2(1), ct1-Ru1-N2 152.2(1).

SupplementaryTable 7. Crystal data and structure refinement for **6**

Empirical formula	$C_{51}H_{41}N_2OPRu$
Formula weight	829.90
Temperature/K	100.9
Crystal system	triclinic
Space group	P-1
a/Å	9.0052(7)
b/Å	10.3535(8)
c/Å	20.6113(15)
α/°	93.427(3)
β/°	92.616(3)
γ/°	99.282(3)
Volume/Å3	1890.2(2)
Z	2
ρcalcmg/mm3	1.458
m/mm 1	0.501
F(000)	856.0
Crystal size/mm3	0.12 × 0.08 × 0.06
Radiation	MoKα (λ = 0.71073)
2Θ range for data collection	4.58 to 56.68°
Index ranges	-12 ≤ h ≤ 11, -13 ≤ k ≤ 13, -27 ≤ l ≤ 27
Reflections collected	40501
Independent reflections	9357 [Rint = 0.0657, Rsigma = 0.0724]
Data/restraints/parameters	9357/516/505
Goodness-of-fit on F2	0.965
Final R indexes [I>=2σ (I)]	R1 = 0.0461, wR2 = 0.1276
Final R indexes [all data]	R1 = 0.0774, wR2 = 0.1444
Largest diff. peak/hole / e Å-3	0.90/-0.53

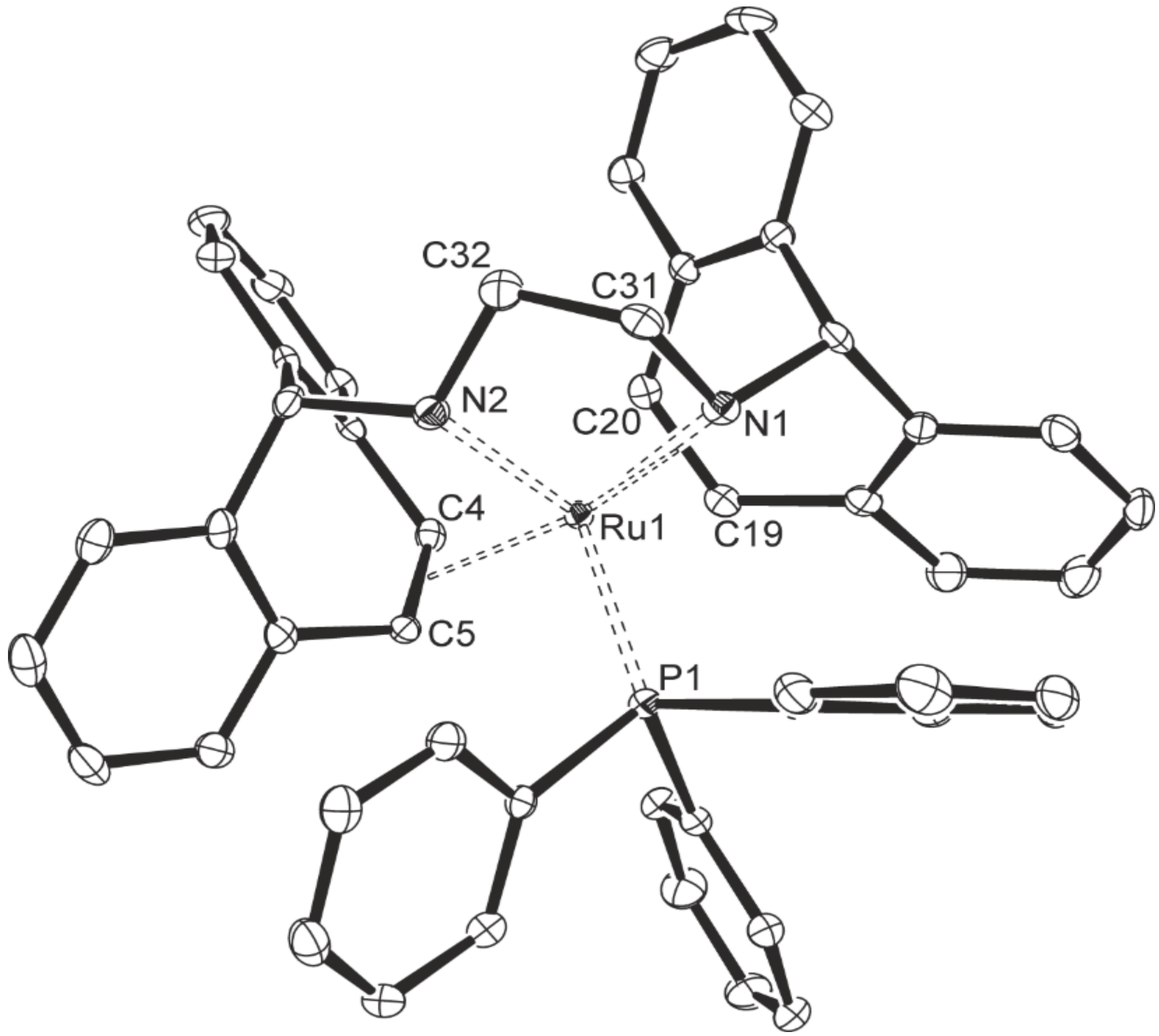

Supplementary Figure 19. ORTEP plot of [Ru(trop$_2$dae)(PPh$_3$)].(dme) (**7**). Ellipsoids are shown at 50% probability. Solvent molecule and hydrogen atoms are omitted for clarity. Selected bond lengths (Å) and angles (°): Ru-N1 2.175(2) , Ru-N2 2.202(2), Ru-P1 2.288(1), Ru-ct1 2.052(2), Ru-ct2 2.060(2), C4-C5 1.459(3), C19-C20 1.422(3), N1-C31 1.494(3), N2-C32 1.474(3), C31-C32 1.502(3); N1-Ru-P1 92.2(1), N2-Ru-P1 111.8(1), ct1-Ru-P1 118.9(1), ct2-Ru-P1 96.3(1), N1-Ru-ct1 84.9(1), N1-Ru-ct2 166.0(1), N1-Ru-N2 79.9(1), N2-Ruct1 127.3(1), N2-Ru-ct2 86.6(1).

Supplementary Table 8. Crystal data and structure refinement for **7**.(dme)

Empirical formula	$C_{54}H_{53}N_2O_2PRu$
Formula weight	894.02
Temperature/K	100(2)
Crystal system	monoclinic
Space group	P21/c
a/Å	12.5513(2)
b/Å	13.0423(2)
c/Å	25.6431(4)
α/°	90.00
β/°	91.3140(10)
γ/°	90.00
Volume/Å^3	4196.62(11)
Z	4
ρcalcg/cm^3	1.415
μ/mm^{-1}	0.458
F(000)	1864.0
Crystal size/mm^3	0.6 × 0.23 × 0.1
Radiation	MoKα (λ = 0.71073)
2Θ range for data collection/°	3.18 to 54.2
Index ranges	-15 ≤ h ≤ 16, -16 ≤ k ≤ 16, -26 ≤ l ≤ 32
Reflections collected	43644
Independent reflections	9182 [Rint = 0.0680, Rsigma = 0.0580]
Data/restraints/parameters	9182/1/770
Goodness-of-fit on F2	0.958
Final R indexes [I>=2σ (I)]	R1 = 0.0325, wR2 = 0.0676
Final R indexes [all data]	R1 = 0.0442, wR2 = 0.0711
Largest diff. peak/hole / e Å^{-3}	0.73/-0.58

7. Calculations

All DFT geometry optimizations were carried out with the Turbomole program [4] coupled to the PQS Baker optimizer [5] via the BOpt package.[6] Geometries were fully optimized as minima or transition states using the BP86 functional[7,8] and the resolution-of-identity (ri) method[9] using the Turbomole def2-TZVP basis [10] for all atoms. Grimme's dispersion corrections (D3 version, implemented with the keyword disp3 in Turbomole) were applied in all geometry optimizations.[11] All minima (no imaginary frequencies) and transition states (one imaginary frequency) were characterized by calculating the Hessian matrix. ZPE and gas-phase thermal corrections (entropy and enthalpy, 298 K, 1 bar) from these analyses were calculated. The relative (free) energies obtained from these calculations are reported in the main text of this paper. The nature of the transition states was confirmed by following the intrinsic reaction coordinate (IRC).

By calculation of the partition function of the molecules in the gas phase, the entropy of dissociation or coordination for reactions in solution is overestimated (overestimated translational entropy terms in the gas phase compared to solutions). For reactions in solution we therefore corrected the Gibbs free energies for all steps involving a change in the number of solute species (we did not apply any corrections for loss of gaseous H_2 or CO_2). The applied correction term is a correction for the condensed phase (CP) reference volume (1 L mol^{-1}) compared to the gas phase (GP) reference volume (24.5 L mol^{-1}). This leads to an entropy correction term ($S_{CP} = S_{GP} + R\mathit{ln}\{1/24.5\}$ for all species, which combined with neglecting the RT term, corrects the relative free energies (298 K) of all associative (–2.5 kcal mol^{-1}) and dissociative steps (+2.5 kcal mol^{-1}),[12]

except those involving H_2 and CO_2 gas molecules.

In order to make computations more time efficient and less expensive, a reduced model for catalyst was employed. Li and Hall,[13] used this approach on same system and it has been established that MERP is not affected by this simplification in any significant way.

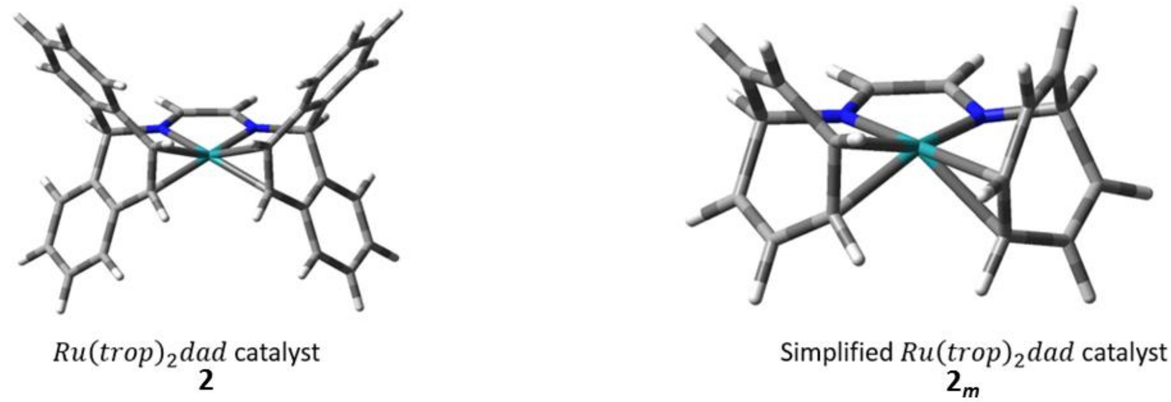

Supplementary Figure 20. Full-atom (left) geometry of catalyst **2** and its simplified (right) models (**2**$_m$). The same simplification was also applied to all other computed species reported in this paper.

Unless stated otherwise, all energies are gas-phase relative energies in kcal mol^{-1}. Numbers and letters shown in bold denote catalytic complexes and intermediates respectively.

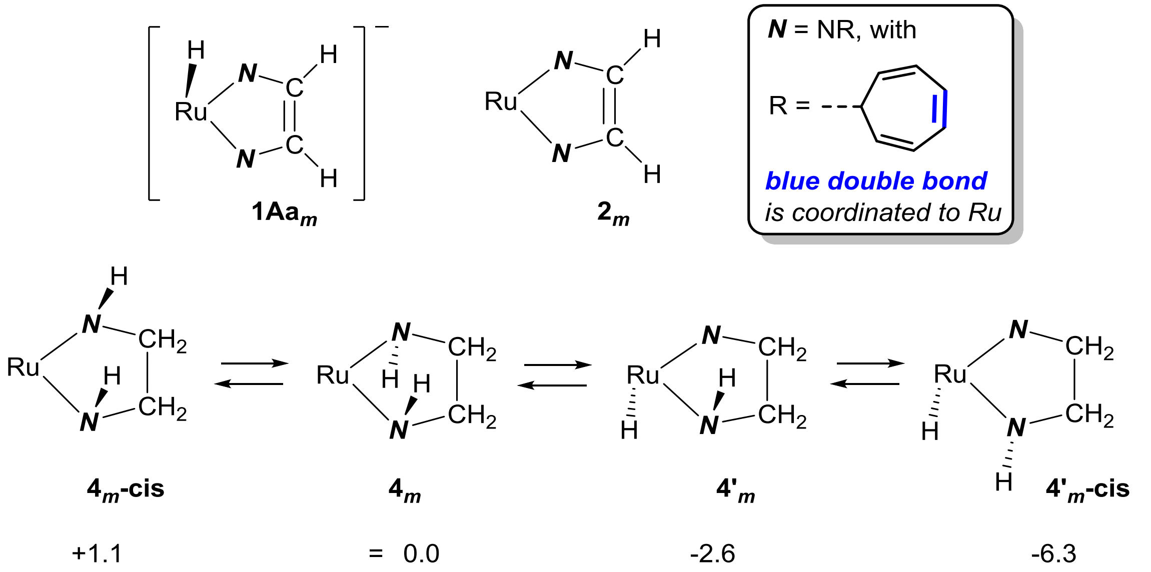

Supplementary Figure 21. Complex **1Aa**$_m$ and **2**$_m$. Gas phase relative free energies ($\Delta G°_{298K}$ in kcal mol^{-1}) of **4**$_m$, **4**$_m$**-cis, 4'**$_m$ and **4'**$_m$**-cis**.

Experimental observations revealed that ruthenium complex **2**$_m$ easily converts to the complexes **4**$_m$ and **4'**$_m$ (Supplementary Figure **21**) during the catalytic reactions. Complex **1Aa** is known to undergo

protonation by exogenous proton donors to produce neutral complex **2**, which under the catalytic conditions of methanol dehydrogenation gets reversibly hydrogenated to produce complex **4**. The latter contains a saturated RNH-CH_2-CH_2-NHR ligand backbone. Experimentally, complex **4** is known to convert back to complex **2** upon heating. Li and Hall[13] recently explored the mechanism of hydrogen production from anionic complex **1Aa***m*. The mechanistic considerations in the paper of Li and Hall are based on the hypothesis is that H_2 is formed via a purely ligand-based mechanism, in which H_2 is always released from the hydrogenated dad-ligand backbone, and in which metal-hydrides play no role. The report elegantly describes a variety of accessible pathways to convert **1Aa***m* into analogous (anionic and neutral) complexes with a saturated N-CH_2-CH_2-N backbone. However, in the proposed purely ligand-based mechanism the reported overall barrier for H_2-release from the saturated ligand backbone is rather high (>30 kcal mol^{-1}). Such a high barrier does not seem to be consistent with the high turnover frequencies observed experimentally. Hence, however elegant, this study does not seem to have caught the catalytically relevant H_2-production steps. Jing & coworkers[14] also recently reported mechanistic DFT studies on the methanol–water dehydrogenation reaction mediated by the (trop$_2$dad)Ru system. However, with exception to some additional dehydrogenation pathways which involve Ru, their mechanistic study is essentially identical to that reported by Li & Hall. Nonetheless, these studies have revealed several accessible pathways to convert the ruthenium complexes with an unsaturated N=CH-CH=N (dad) ligand backbone into those with a saturated N-CH_2-CH_2-N backbone, as was also experimentally observed. Hence, besides species **2***m* (see main text), also complexes **4'***m* (**4'***m***-cis**) containing a saturated ligand backbone (Supplementary Figure 21) should be considered as potentially active species in catalytic formaldehyde dehydrogenation, and species **4***m*, **4***m***-cis, 4'***m* and **4'***m***-cis** are likely to co-exist under the experimental reaction conditions. Hence we decided to explore this computationally.

We first explored the conversion of species **4***m* to **4'***m*, which is exergonic and proceeds over a rather low-barrier transition state (Supplementary Figure 22). This is essentially a proton transfer reaction from the ligand to the metal.[15,16]

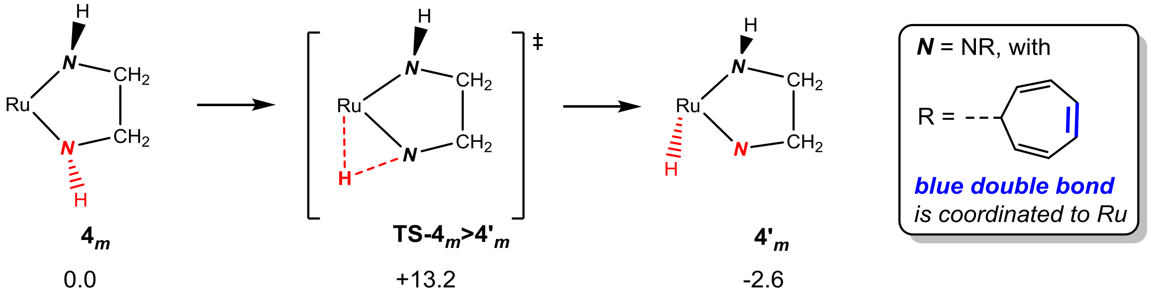

Supplementary Figure 22. Conversion of **4**$_m$ to **4'**$_m$ via **TS-4**$_m$**>4'**$_m$ (gas phase relative free energies, $\Delta G°_{298K}$, in kcal mol^{-1}).

Catalytic dehydrogenation of methanediol mediated by complex 4'$_m$

Complex **4'**$_m$ seems particularly relevant in the catalytic reaction, especially in the initial steps of the methanediol activation process, as substrate binding and activation by species **4'**$_m$ will be facilitated by double hydrogen-bonding interactions between the substrate and the ligand (Supplementary Figure 23). Involvement of **4'**$_m$**-cis** in the catalytic process can of course not be disregarded, but in contrast to **4'**$_m$ the corresponding intermediates are stabilized by only a single hydrogen-bond for **4'**$_m$**-cis** due to its anti-periplanar orientation of the N–H bond with respect to the coordinated substrate. This leads to a weaker substrate binding affinity and higher transition state barriers. Therefore, we focused our studies primarily on the catalytic involvement of species **4'**$_m$. However, in the final H_2-releasing steps where hydrogen-bonding between the substrate and the ligand is no longer relevant the involvement of **4'**$_m$**-cis** could well be important (vide infra).

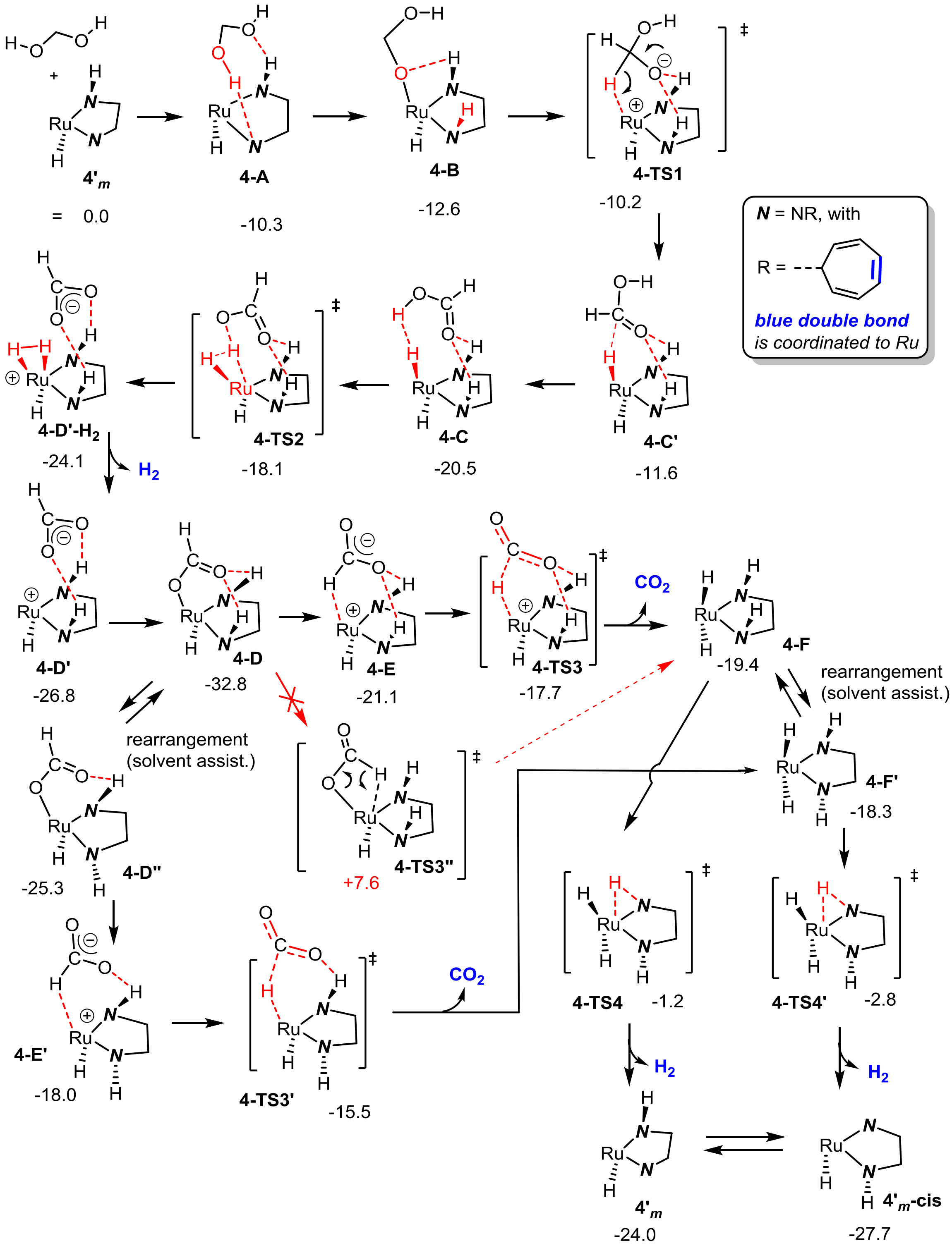

Supplementary Figure 23. Computed pathway (Turbomole, DFT-D3 (disp3), BP86, def2-TZVP) for methanediol dehydrogenation catalysed by complex **4'**$_m$, along with their relative free energies ($\Delta G°_{298K}$ in

kcal mol^{-1}). All energies (also the transition states) relative to the starting materials (complex **4'$_m$** + methanediol).

The computed pathway from **4'$_m$** involves initial association of methanediol via a double hydrogen-bond interaction between the ligand and the substrate producing **4-A**, which is exergonic by 10 kcal mol^{-1}. Proton-transfer to the amido moiety and coordination of the alcoholate moiety to Ru is downhill by another 2-3 kcal mol^{-1}, producing complex **4-B**. Subsequent hydride transfer from methanediol to Ru over **4-TS1** has a remarkably low barrier (2.4 kcal mol^{-1}). This is not a common beta-hydride elimination step, as was computed for complex **2$_m$** (see main text), but is better described as an ion-pair polarized, double hydrogen-bond stabilized transition state without a significant Ru–O interaction (Ru–O distance: 3.52 Å). This step directly produces intermediate **4-C'**, which is best described as a formic acid adduct stabilized by two hydrogen-bonds involving the amine moieties of the ligand, as well as by a weak interaction between the hydride and the carbonyl moiety ($C_{carbonyl}$-$H_{hydride}$ distance: 2.08 Å). Complex **4-C'** then rearranges to form complex **4-C**, which has a distinct dihydrogen bond between the Ru–H moiety and the acidic proton of the formic acid moiety ($H_{hydride}$–H_{acid} distance: 1.20 Å) and still contains a double hydrogen bond interaction between the two amines and the carbonyl group of the formic acid moiety.

From **4-C**, protonation of the hydride effectively produces sigma-hydrogen complex **4-D'-H$_2$** over a low barrier (2.5 kcal mol^{-1}) transition state (**4-TS2**). Subsequent loss of dihydrogen drives the reaction forward to **4-D'**, which rearranges to formate complex **4-D**, again stabilized by a double hydrogen bonding interaction between the formate carbonyl group and the two amine moieties of the ligand. Subsequent **r**earrangement to **4-E** has an energy penalty of +12 kcal mol^{-1}, but is necessary to place the formate hydrogen atom in position for hydride transfer to Ru. Once formed, the additional barrier for hydride transfer and concomitant CO_2 loss from **4-E** is low (3.4 kcal mol^{-1}). The overall barrier for formation of **4-F** from **4-D** over **4-TS3** is 15 kcal mol^{-1}. This reaction step is endergonic (+13 kcal mol^{-1}), but experimentally driven to completion by capture of CO_2 under the applied basic conditions. Again this hydride transfer step is not a common beta-hydride elimination step (as was computed for complex **2'$_m$**, see main text), but just like **4-TS1** the process is best described as an ion-pair polarized, double hydrogen-bond stabilized transition state without a significant Ru–O interaction (Ru–O distance: 3.71 Å). Direct beta-hydride

elimination from the metal-bound formate in **4-D** over **4-TS3''** has a prohibitive high barrier (+40 kcal mol^{-1}), and hence is not a viable pathway.

Subsequent H_2-elimination from **4-F** involves proton transfer from an amine moiety of the ligand to the metal and H_2-reductive elimination over **4TS4** to produce **4'**$_m$, which is exergonic. Directly from **4-F** this process has a barrier of +18 kcal mol^{-1}, which is certainly accessible under the experimentally applied reaction conditions, but the barrier lowers to +16.6 kcal mol^{-1} upon prior rearrangement of **4-F** to **4-F'** and subsequent formation of H_2 and **4'**$_m$**-cis** over **4TS4'**.

We further investigated the effect of flipping one of the amine protons in **4-D** to an anti-periplanar configuration with respect to the coordinated formate in **4-D''**, which may happen in course of the catalytic cycle. This leads to a very similar pathway as computed from **4-D**, but with a slightly higher overall barrier (+17.3 versus +15.1 kcal mol^{-1}). This is as expected, as in this pathway from **4-D''** the intermediates and transition states are stabilized by only one hydrogen bond instead of two.

As mentioned in the main text, the computed pathways for methanediol dehydrogenation by both catalysts **1Aa**$_m$ (and consequently **2**$_m$) and **4'**$_m$ provide viable pathways for formaldehyde dehydrogenation, and it is quite likely that both pathways contribute to the observed catalytic activity. In both pathways hydride migration from the substrate to the metal are key steps in the catalytic cycle to produce H_2, and in both mechanisms metal-ligand cooperativity plays an important role.

Free energy profiles of calculated catalytic cycles

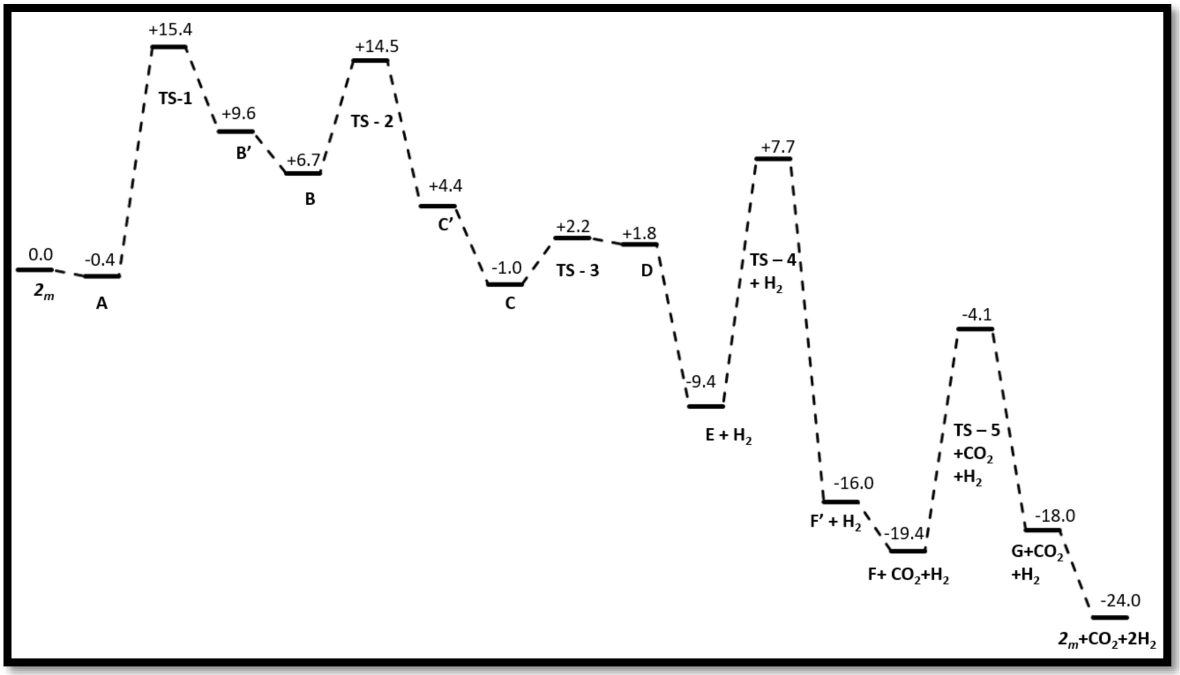

Supplementary Figure 24. Methanediol dehydrogenation by catalyst complex $\mathbf{2}_m$.

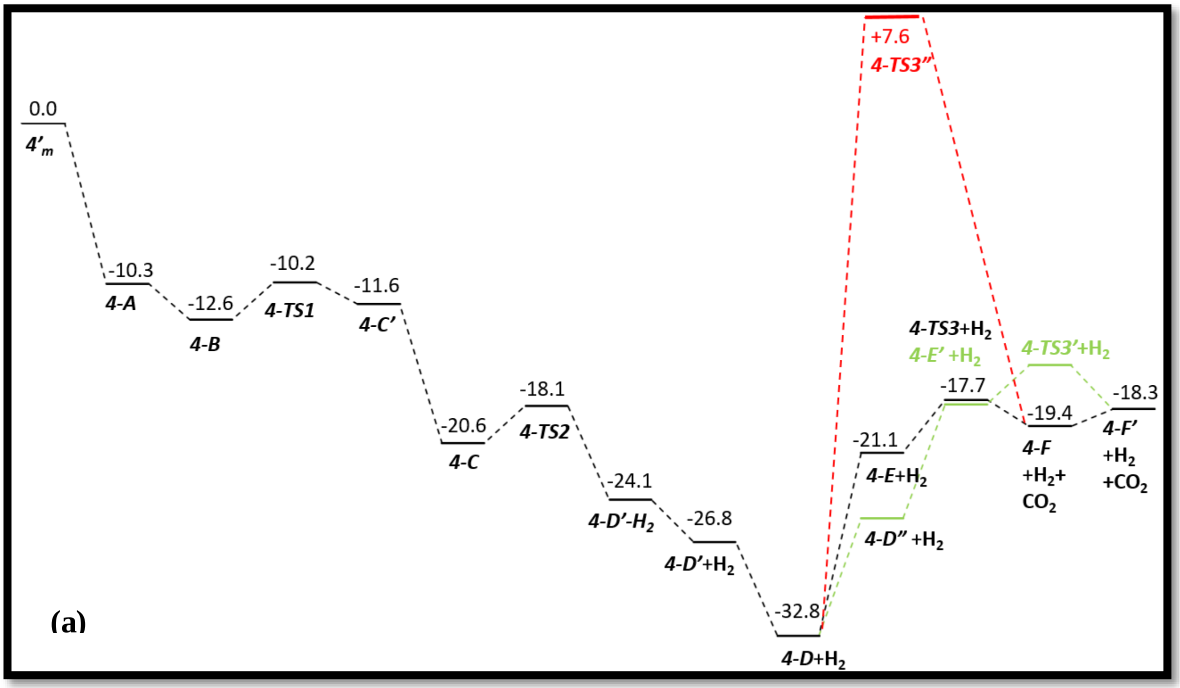

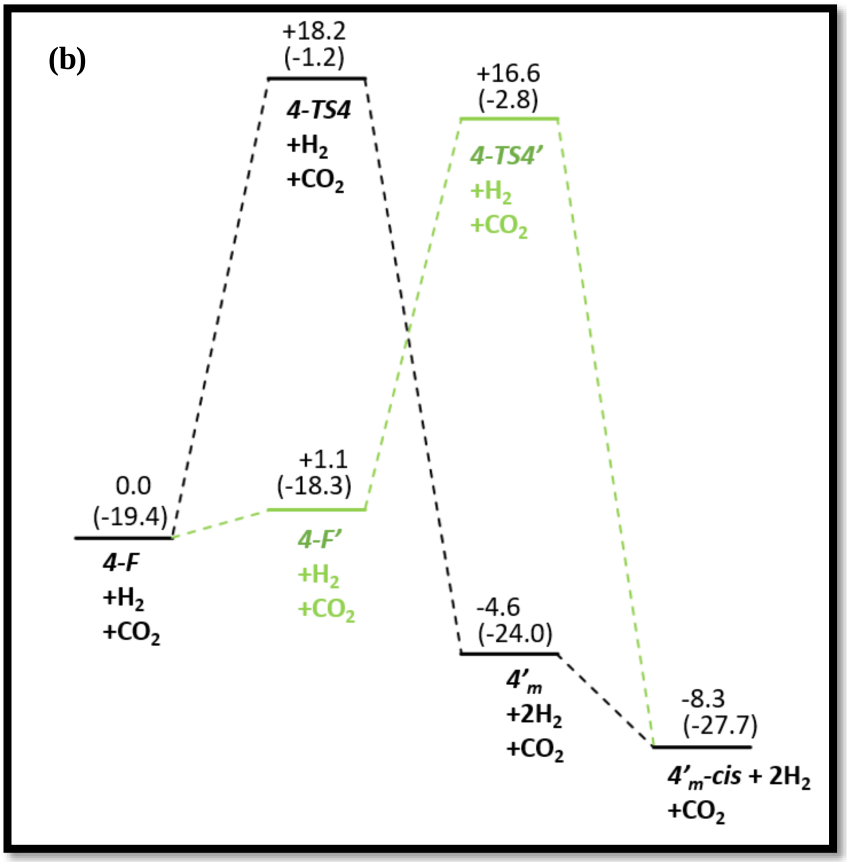

Supplementary Figure 25. Methanediol dehydrogenation by catalyst complex ***4**$_m$*. (a) Reaction energy profile showing generation of complex **4-F** and **4-F'** from methanediol. The pathway marked with red lines shows the high barrier pathway for formate oxidation via a classical beta-H elimination. The pathway marked with green lines indicates an alternative pathway after flipping a proton on complex ***4-D***. (b) Since the reactions are driven forward by escape of gaseous H_2 and CO_2 from the reaction mixture, the barriers for the final dehydrogenation steps leading to formation of **4'm** and **4'm-cis** via **4-TS4** and **4-TS4'** must be computed from species **4-F** rather than **4-D**. Hence these steps are visualized separately. The values in parenthesis are energy values with respect to the starting materials.

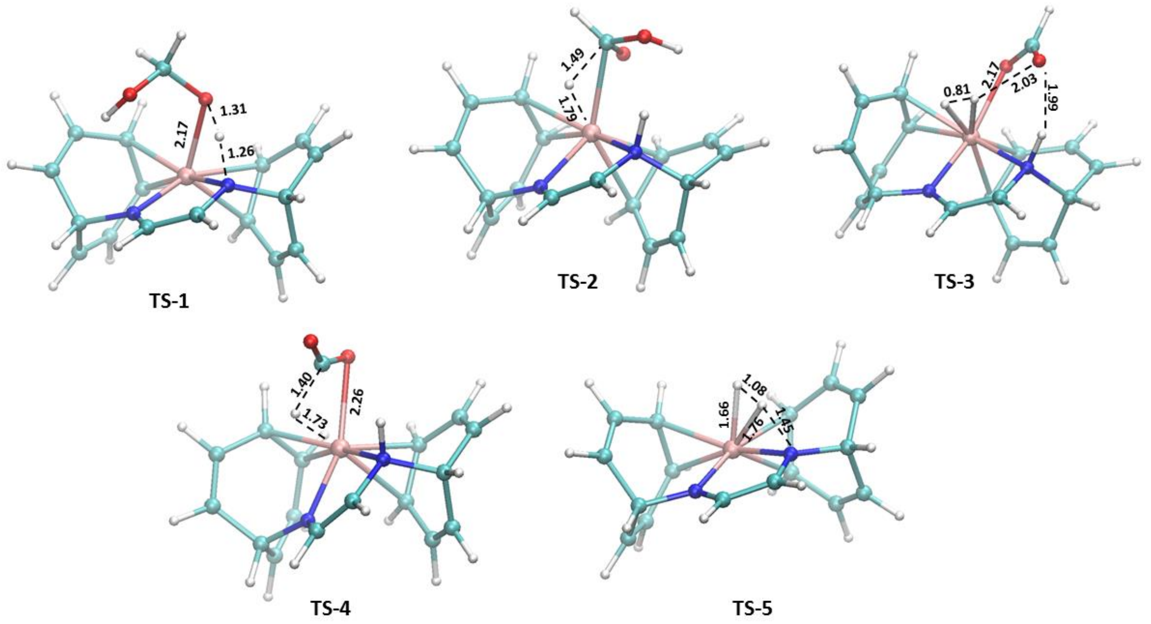

Supplementary Figure 26. Graphical representation of transition states **TS-1** to **TS-5** (dashed lines indicate relevant bond lengths (Å) in the transition state)

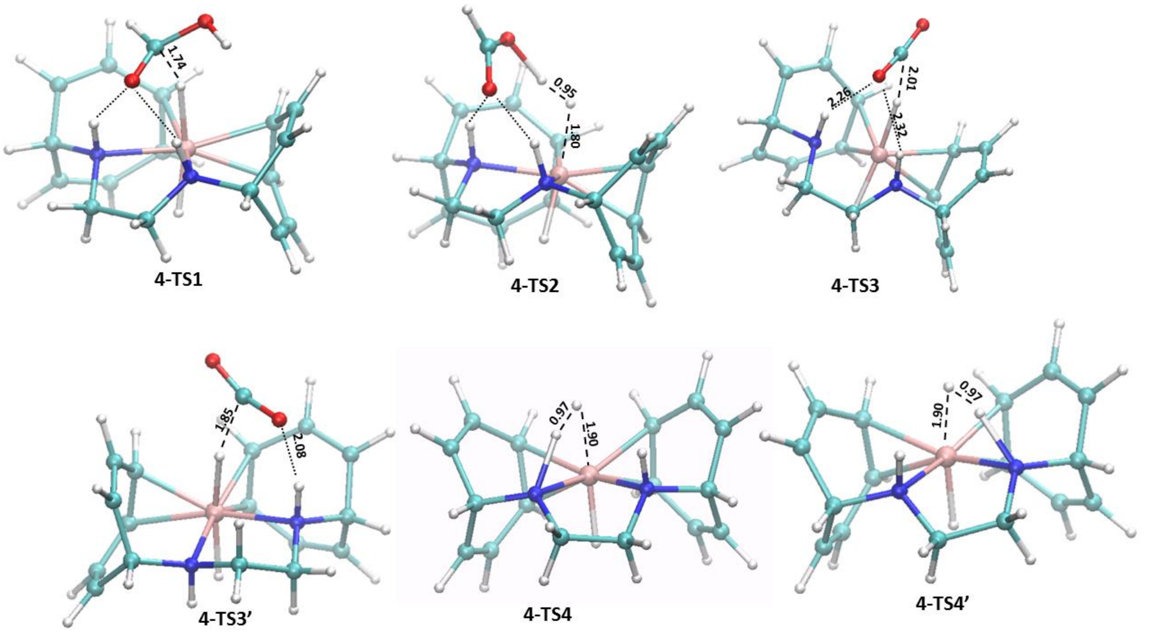

Supplementary Figure 27. Graphical representation of transition states **4-TS1** to **4-TS4'** (dotted lines indicate H-bonding, thicker dashed lines indicate relevant bond lengths (Å) in the transition state)

Supplementary Table 9

Unsaturated ligand system

species	SCF	ZPE	H_{corr}	G_{corr}	SCF+ZPE	H	G^{o}_{298K}
CO_2	-188.69	0.0113	0.01489	-0.0094	-188.6764	-188.67282	-188.69712
H_2	-1.1775	0.00986	0.01307	-0.0018	-1.16767	-1.16446	-1.17936
2_m	-824.18	0.27362	0.28981	0.23304	-823.9017	-823.8855	-823.94227
methanediol	-191.05	0.05479	0.05981	0.02958	-190.9924	-190.98737	-191.0176
A	-1015.2	0.33049	0.35159	0.28357	-1014.91	-1014.88845	-1014.95647
TS1	-1015.2	0.32576	0.34579	0.28123	-1014.887	-1014.86684	-1014.9314
B'	-1015.2	0.3311	0.35173	0.2854	-1014.895	-1014.87422	-1014.94055
B	-1015.2	0.33042	0.35089	0.28509	-1014.9	-1014.87946	-1014.94526
TS2	-1015.2	0.32769	0.34766	0.28367	-1014.889	-1014.86873	-1014.93272
C'	-1015.2	0.32962	0.35009	0.28498	-1014.904	-1014.88383	-1014.94894
C	-1015.2	0.32802	0.34848	0.28279	-1014.912	-1014.89173	-1014.95742
TS3	-1015.2	0.32576	0.34581	0.28193	-1014.909	-1014.88856	-1014.95244
D	-1015.2	0.32625	0.34689	0.2816	-1014.908	-1014.88765	-1014.95294
E	-1014.1	0.30832	0.32798	0.26399	-1013.751	-1013.73156	-1013.79555
TS4	-1014	0.30636	0.3259	0.26215	-1013.724	-1013.70445	-1013.7682
F'	-1014.1	0.30589	0.32697	0.25704	-1013.757	-1013.73607	-1013.806
F	-825.37	0.29366	0.31041	0.25249	-825.0732	-825.05647	-825.11439
TS5	-825.34	0.28689	0.3032	0.24649	-825.0496	-825.03326	-825.08997
G	-825.36	0.2887	0.30587	0.2466	-825.0699	-825.05274	-825.11201
2_m	-188.69	0.0113	0.01489	-0.0094	-188.6764	-188.67282	-188.69712

Saturated Ligand system

Species	SCF	ZPE	H_{corr}	G_{corr}	SCF+ZPE	H	G^{o}_{298K}
$4'_m$	-826.58	0.31733	0.33428	0.27586	-826.2649	-826.24796	-826.30638
4-A	-1017.7	0.37486	0.39666	0.32686	-1017.288	-1017.26659	-1017.33639
4-B'	-1017.7	0.37676	0.39775	0.33127	-1017.294	-1017.27273	-1017.33921
4-B	-1017.7	0.37707	0.39818	0.33098	-1017.294	-1017.27292	-1017.34012
4-TS1	-1017.7	0.37276	0.39323	0.32776	-1017.291	-1017.27085	-1017.33632
4-C'	-1017.7	0.37313	0.39457	0.3258	-1017.291	-1017.26972	-1017.33849
4-C	-1017.7	0.3717	0.3931	0.32386	-1017.305	-1017.28345	-1017.35269
4-TS2	-1017.7	0.37054	0.39127	0.32467	-1017.303	-1017.28232	-1017.34892
4-D'-H2	-1017.7	0.3699	0.39173	0.32253	-1017.311	-1017.28922	-1017.35842
4-D'	-1016.5	0.35267	0.37379	0.30481	-1016.14	-1016.11839	-1016.18737
4-D	-1016.5	0.35592	0.37631	0.31105	-1016.152	-1016.13156	-1016.19682
4-E	-1016.5	0.35361	0.37417	0.30757	-1016.132	-1016.11161	-1016.17821
4-TS3	-1016.5	0.35066	0.37118	0.30344	-1016.126	-1016.10515	-1016.17289
4-TS3"	-1016.4	0.34946	0.3704	0.30247	-1016.086	-1016.0646	-1016.13253
4-F	-827.78	0.33879	0.3558	0.29781	-827.4375	-827.42044	-827.47843
4-F'	-827.77	0.3378	0.35486	0.29746	-827.4363	-827.4192	-827.4766
4-TS4'	-827.74	0.33162	0.34889	0.28936	-827.4071	-827.38985	-827.44938
4-D1	-1016.5	0.35453	0.37502	0.30951	-1016.14	-1016.11951	-1016.18502
4-TS3'	-1016.5	0.34994	0.37041	0.30363	-1016.123	-1016.10248	-1016.16926
4-E’	-1016.5	0.35249	0.37296	0.30707	-1016.128	-1016.10738	-1016.17327
4-G	-827.77	0.3378	0.35486	0.29746	-827.4363	-827.4192	-827.4766
$4'_m$ - cis	-826.59	0.3174	0.33429	0.27621	-826.2711	-826.25421	-826.31229
4-TS4	-827.74	0.33179	0.34897	0.29027	-827.407	-827.38983	-827.44853
4_m-cis	-826.578	0.32035	0.33773	0.27777	-826.25782	-826.24044	-826.3004
TS-4_m >$4'_m$	-826.56	0.31503	0.33185	0.27386	-826.24	-826.22319	-826.28118

**4**$_m$	-826.58	0.32017	0.33751	0.27854	-826.2607	-826.24332	-826.30229

$$\Delta G = free\ energy$$

$$\Delta H = enthalpy$$

$$\Delta G_{coor} = correction\ term\ to\ free\ enegry$$

$$\Delta H_{corr} = correction\ term\ to\ enthalpy$$

All values in the Supplementary Table are in atomic units.

OPTIMIZED GEOMETRIES

All energy values reported below (combined with the xyz-coordinates) are SCF energies in atomic units.

Optimized-coordinates (XYZ format)

H_2

2

Energy = -1.1775301414

H 0.0000000 0.0000000 -0.0254195

H 0.0000000 0.0000000 0.7254195

2*$_m$*

35

Energy = -824.1753112354

C 0.1312397 0.8294883 0.8506095

H -0.1523866 1.5224509 1.6462123

C 0.5870400 -0.4775814 1.0722215

H 0.6042523 -0.9717265 2.0468925

N 0.0643967 1.1817538 -0.4459898

N 0.9912514 -1.0936173 -0.0382227

C -0.4829081 2.5130701 -0.8073060

H -0.7703238 3.0489665 0.1107775

C 1.1836225 -2.5460945 -0.2116248

H 0.9158220 -3.1074816 0.6980064

C 0.5538494 3.3071637 -1.5720792

H 0.7676023 4.3241796 -1.2422453

C 1.1834055 2.7699613 -2.6380978

H 1.9540715 3.3610919 -3.1404964

C -1.6876324 2.3513484 -1.7127014

H -2.5987516 2.8886802 -1.4478069

C -1.6309299 1.5890254 -2.8273502

H -2.5426031 1.4793205 -3.4216477

C 0.1656679 -2.8018608 -1.3059371

H -0.7698954 -3.2791562 -1.0088794

C 0.2663148 -2.3411752 -2.6190598

H -0.5965860 -2.5515735 -3.2585833

C 2.6161730 -2.7934941 -0.5978116

H 3.2037050 -3.5026706 -0.0139783

C 3.1828797 -2.0574783 -1.5744245

H 4.2619978 -2.1487907 -1.7353971

Ru 0.6229367 -0.1599431 -1.7909552
C 0.9065733 1.4478547 -3.1959165
H 1.5993040 1.1544269 -3.9919516
C -0.4427639 0.8967807 -3.3248797
H -0.6164029 0.2881327 -4.2183866
C 2.4824847 -1.1568336 -2.5030391
H 3.1514606 -0.4983989 -3.0649261
C 1.3204490 -1.5832856 -3.2305894
H 1.2087453 -1.3193763 -4.2855431

A
42
Energy = -1015.2400414730
C -1.0437830 -1.0293938 -1.6644570
C -1.3830826 0.3820350 -1.5610266
C 1.7743059 -1.0095084 -0.9163462
C -1.9929925 -2.1035102 -1.3588821
C -2.6351298 0.8634901 -0.9847489
C 1.8570165 0.4511371 -1.0273597
C 2.7242552 -1.7762220 -0.0990623
C -2.8493838 -2.1123030 -0.3174432
C 2.6568230 1.2612439 -0.1219239
C -3.3316992 0.2689306 0.0143810
C 3.1069081 -1.4642674 1.1553090
C -2.8330775 -0.9900513 0.6971927
C 2.9304020 0.9813415 1.1861399
C 2.4571296 -0.2944549 1.8636856
C -1.1411398 -0.9254944 2.4678234
C 0.2183902 -0.7319316 2.7737325
H -0.3737480 -1.2691917 -2.4954215
H -0.9385528 1.0354751 -2.3170493
H 1.5419946 -1.5103098 -1.8597741
H 1.7162965 0.8674897 -2.0294832
H -3.4806912 -1.2395776 1.5529800
H 2.7375513 -0.2824253 2.9296605
H -1.8989600 -1.1878198 3.2102169
H 0.6313777 -0.8312913 3.7817200
N -1.4398753 -0.7865168 1.1668819
N 0.9932796 -0.4446761 1.7236404
O -0.0534713 2.1529832 0.5589014
Ru 0.0269385 -0.1352456 -0.0075738
H -3.0331119 1.7948235 -1.3975505
H -4.2866150 0.6801199 0.3439736
H -3.5714867 -2.9183860 -0.1832800
H -2.0050244 -2.9509978 -2.0501443
H 3.1683788 -2.6543098 -0.5765535
H 3.8709853 -2.0373558 1.6814128
H 3.5855208 1.6418873 1.7563824
H 3.0515838 2.2004093 -0.5235809
H 0.9004358 2.1924594 0.8159679
C -0.8636040 2.4739632 1.7056871
H -0.5706895 1.8329924 2.5555302
H -1.8905307 2.2517732 1.3961621
O -0.7968457 3.8400936 2.0294314

H 0.0038059 3.9976827 2.5590001

TS1
42
Energy = -1015.2126331250
C -1.1038076 -0.6311880 -1.6353151
C -1.5916527 0.6682696 -1.2248155
C 1.6179141 -1.1011685 -0.7510485
C -1.8423284 -1.8720508 -1.4006408
C -2.8750739 0.8340170 -0.5347453
C 1.8784444 0.2930507 -1.1064747
C 2.4365531 -1.8563426 0.2039048
C -2.5550892 -2.1627461 -0.2914688
C 2.8781093 1.1018038 -0.4278268
C -3.3799792 -0.0075197 0.3978890
C 2.9397195 -1.3837868 1.3625841
C -2.5887859 -1.2144131 0.8928993
C 3.2463462 1.0029842 0.8724844
C 2.6032337 0.0204254 1.8265709
C -0.8231411 -0.9034273 2.6027747
C 0.4210979 -0.4349115 2.8892076
H -0.4885539 -0.6310953 -2.5399000
H -1.2816840 1.5113758 -1.8469145
H 1.2452764 -1.7192943 -1.5712531
H 1.6890342 0.5595202 -2.1500017
H -3.1039085 -1.6822160 1.7458284
H 2.9884225 0.1836347 2.8471327
H -1.4956644 -1.3269947 3.3519629
H 0.8783286 -0.5049545 3.8780516
N -1.2264835 -0.7970419 1.2752549
N 1.1211191 0.2010047 1.8453318
O 0.3672364 2.1458428 0.5798367
Ru 0.0151022 0.0647591 0.0825124
H -3.4819139 1.6936813 -0.8327043
H -4.3930118 0.1254822 0.7797249
H -3.1533456 -3.0720908 -0.2291678
H -1.8169463 -2.6106541 -2.2061565
H 2.6695618 -2.8855575 -0.0831140
H 3.6288595 -1.9818652 1.9605848
H 4.0330523 1.6465424 1.2668978
H 3.3515479 1.8858843 -1.0248730
H 0.8068620 1.3808528 1.5416132
C -0.7455722 2.9205723 0.9773292
H -1.3757069 3.1280785 0.0916456
H -0.3468572 3.8687643 1.3758908
O -1.5157047 2.3557733 2.0217989
H -2.0907258 1.6720898 1.6208611

B'
42
Energy = -1015.2259532830
C -1.3084783 -0.3061417 -1.5518126
C -2.0425224 0.6856051 -0.8246545
C 1.2813551 -0.8915530 -0.6695206

C	-1.7400624	-1.6940744	-1.6932027
C	-3.2938622	0.3916073	-0.1269033
C	1.5272949	0.5627741	-0.7544502
C	2.1182660	-1.8136679	0.1017343
C	-2.3425470	-2.4256545	-0.7316068
C	2.5801340	1.2263785	0.0068044
C	-3.5901027	-0.7699154	0.4950495
C	2.6691836	-1.5647603	1.3060266
C	-2.5473086	-1.8664419	0.6625052
C	3.0013801	0.8977318	1.2489380
C	2.4019994	-0.2574304	2.0145090
C	-0.8068611	-1.7469887	2.4057666
C	0.3157816	-1.1871579	2.9114291
H	-0.7089655	0.0748566	-2.3839041
H	-1.9402691	1.7115077	-1.1805596
H	0.9310240	-1.3453930	-1.5968495
H	1.3297395	1.0181395	-1.7292237
H	-2.9300817	-2.6531609	1.3322358
H	2.8227413	-0.2923263	3.0333236
H	-1.3330938	-2.5461880	2.9386178
H	0.8149040	-1.5156948	3.8217480
N	-1.3019977	-1.3000287	1.2009916
N	0.9050139	-0.1013174	2.1347152
O	-0.2785408	2.0649435	0.9635198
Ru	-0.2780832	0.1363626	0.3489583
H	-4.0542899	1.1759108	-0.1566233
H	-4.5936469	-0.9570423	0.8796586
H	-2.7077185	-3.4318837	-0.9409155
H	-1.5607438	-2.1607380	-2.6654133
H	2.3002077	-2.7888487	-0.3588953
H	3.3288166	-2.2907952	1.7816111
H	3.8345533	1.4303415	1.7085711
H	3.0521914	2.0797903	-0.4849693
C	-0.3243720	3.2638493	0.2912636
H	-0.8431118	3.9878378	0.9581917
H	-0.8670911	3.2251377	-0.6729973
O	0.9705189	3.7699099	-0.0588546
H	1.5336590	3.5793637	0.7148357
H	0.7040721	0.8270157	2.5435669

B

42

Energy = -1015.2303519750

C	-1.1657005	-0.3957580	-1.7060693
C	-1.9008976	0.6747419	-1.1005545
C	1.3648595	-1.0519818	-0.5726082
C	-1.6617992	-1.7675302	-1.7718440
C	-3.2214395	0.4999038	-0.5007425
C	1.7094033	0.3544226	-0.7489177
C	2.0673529	-1.9447954	0.3508606
C	-2.3717741	-2.3823118	-0.8007935
C	2.6815189	1.0599293	0.0698642
C	-3.6316464	-0.5877305	0.1873154
C	2.5211725	-1.6160998	1.5767502

C	-2.6557722	-1.7034105	0.5257453
C	2.9658731	0.8173738	1.3707711
C	2.2456773	-0.2479895	2.1597278
C	-1.0719686	-1.5203840	2.4017470
C	0.0604248	-1.0070785	2.9319523
H	-0.4919058	-0.1044969	-2.5171381
H	-1.6738692	1.6794284	-1.4595789
H	1.0394139	-1.5634049	-1.4795300
H	1.5827513	0.7546266	-1.7574109
H	-3.1235283	-2.4179709	1.2213062
H	2.5734924	-0.2194820	3.2124358
H	-1.6977502	-2.2319759	2.9495300
H	0.4695096	-1.2838613	3.9027603
N	-1.4293157	-1.1423156	1.1222485
N	0.7562303	-0.0228276	2.1284972
O	-0.0286936	2.2180871	0.1667107
Ru	-0.2474806	0.1173811	0.2301994
H	-3.9312235	1.3153438	-0.6611149
H	-4.6692801	-0.6974735	0.5045497
H	-2.7867646	-3.3785756	-0.9596488
H	-1.4496786	-2.3231072	-2.6893238
H	2.2382900	-2.9623040	-0.0109221
H	3.1122520	-2.3216386	2.1613356
H	3.7514758	1.3804176	1.8759804
H	3.2137088	1.8796888	-0.4194347
C	-0.5771229	2.7368308	1.2878076
H	-1.4825217	2.2034837	1.6638441
H	-0.8392508	3.8020962	1.1191994
O	0.3340119	2.6952537	2.4609536
H	1.2033709	2.9391664	2.0822338
H	0.5731547	0.9646293	2.4679868

TS2

42

Energy = -1015.2163882670

C	-1.2260871	-0.0450086	-1.6813971
C	-2.0711738	0.8058501	-0.8913921
C	1.2888968	-1.1227205	-0.5216875
C	-1.5242789	-1.4567411	-1.9128380
C	-3.3098668	0.3761526	-0.2473635
C	1.6913680	0.2367964	-0.7664814
C	1.9028511	-2.0000905	0.4588445
C	-2.0580788	-2.2909996	-0.9947025
C	2.7612234	0.9231226	-0.0451257
C	-3.5043896	-0.8330349	0.3199533
C	2.4229182	-1.6217861	1.6451058
C	-2.3620856	-1.8285464	0.4161306
C	3.0929907	0.7280034	1.2507377
C	2.3070702	-0.2055463	2.1336717
C	-0.9321733	-1.4075529	2.3611519
C	0.0414451	-0.6947708	2.9928384
H	-0.6704212	0.4560972	-2.4793609
H	-2.0302839	1.8643632	-1.1568742
H	0.8417790	-1.6466444	-1.3644458

H	1.5275788	0.6075545	-1.7802035
H	-2.6810636	-2.6926886	1.0222121
H	2.6738192	-0.1413713	3.1708143
H	-1.5200392	-2.1487994	2.9154989
H	0.4022183	-0.9012574	3.9986872
N	-1.1834466	-1.2312054	1.0480180
N	0.8358542	0.2014814	2.1452602
O	0.1044949	2.4944018	-0.2924488
Ru	-0.2237083	0.3844539	0.2506119
H	-4.1190780	1.1110709	-0.2289254
H	-4.4743701	-1.0962016	0.7467018
H	-2.2910350	-3.3230225	-1.2619582
H	-1.2838768	-1.8499204	-2.9045577
H	1.9300125	-3.0608290	0.1989060
H	2.9026747	-2.3531293	2.2950481
H	3.9578310	1.2389796	1.6779267
H	3.3372429	1.6490148	-0.6245074
C	-0.4494286	2.6844428	0.8520203
H	-1.1285136	1.4244737	1.2664694
H	-1.4136677	3.2201239	0.9304840
O	0.3658940	3.0615179	1.9245484
H	1.2790923	2.9572879	1.5747524
H	0.8024328	1.1597213	2.5233339

C'

42

Energy = -1015.2339192450

C	-1.3397187	0.2191701	-1.5599585
C	-2.2497323	0.9222050	-0.6838695
C	1.4541772	-1.1452226	-0.4978194
C	-1.5318876	-1.1804353	-1.9376359
C	-3.4552398	0.3144458	-0.1160469
C	1.9018515	0.1445331	-0.7505188
C	1.8804338	-1.9900055	0.5870139
C	-2.0003836	-2.1480822	-1.1193899
C	2.8493951	0.8766405	0.0525847
C	-3.5338113	-0.9541860	0.3390028
C	2.2813486	-1.5724607	1.8103825
C	-2.3191150	-1.8658754	0.3356753
C	3.0376626	0.7326515	1.3904207
C	2.1769529	-0.1380936	2.2512815
C	-0.9264695	-1.5169688	2.3196477
C	-0.0517078	-0.7479410	3.0291812
H	-0.8219096	0.8433450	-2.2923766
H	-2.3172558	1.9990015	-0.8632442
H	0.8906126	-1.6359276	-1.2918341
H	1.6702849	0.5696647	-1.7273515
H	-2.5691680	-2.8105330	0.8463038
H	2.5183655	-0.0637243	3.2958393
H	-1.4490854	-2.3507499	2.8028929
H	0.2653981	-0.9602777	4.0493009
N	-1.1550797	-1.2702543	1.0118495
N	0.6834876	0.2638171	2.2631137
O	0.2800492	2.4046359	-0.5270160

Ru	-0.4113859	0.5121535	0.3817677
H	-4.3380155	0.9564000	-0.0522850
H	-4.4782125	-1.3463306	0.7216446
H	-2.1719468	-3.1559655	-1.5009004
H	-1.2722897	-1.4439462	-2.9670037
H	1.8470712	-3.0652317	0.3967990
H	2.6637768	-2.2948809	2.5313069
H	3.8296171	1.3027975	1.8812773
H	3.4496547	1.6207911	-0.4767855
C	-0.1151603	2.6981426	0.6479749
H	-1.4992143	1.0130286	1.3850013
H	-1.0832874	3.1855198	0.8439695
O	0.7961583	3.1018994	1.6240651
H	1.6733530	2.8081756	1.2928502
H	0.6343241	1.1837342	2.7161352

C

42

Energy = -1015.2402123560

C	-1.2062720	0.0623128	-1.5527680
C	-2.0841801	0.8054137	-0.6815090
C	1.3730144	-1.1052053	-0.4664034
C	-1.4465292	-1.3404836	-1.8946233
C	-3.3010949	0.2518825	-0.0814248
C	1.7703454	0.2360855	-0.6967651
C	1.9565839	-1.9877671	0.5204697
C	-1.9202445	-2.2741116	-1.0410760
C	2.8146936	0.9243731	0.0450165
C	-3.4155642	-1.0029760	0.4009726
C	2.4512993	-1.6169906	1.7209751
C	-2.2239272	-1.9453259	0.4074095
C	3.1279175	0.7227439	1.3452149
C	2.3374456	-0.2057284	2.2220769
C	-0.8186994	-1.5367297	2.3797231
C	0.0780106	-0.7615838	3.0625989
H	-0.7028748	0.6506638	-2.3258849
H	-2.1305169	1.8808612	-0.8774201
H	0.8702273	-1.6118718	-1.2877308
H	1.5707847	0.6370206	-1.6918341
H	-2.4921801	-2.8701515	0.9451595
H	2.7118128	-0.1557631	3.2572888
H	-1.3556933	-2.3370473	2.9033779
H	0.4314492	-0.9901583	4.0675415
N	-1.0526214	-1.3541237	1.0657216
N	0.8559124	0.1955203	2.2623235
O	0.3286344	2.4697939	-0.1448264
Ru	-0.2346266	0.3829429	0.3884357
H	-4.1560679	0.9299990	-0.0130102
H	-4.3644859	-1.3515918	0.8137395
H	-2.1115638	-3.2895842	-1.3917188
H	-1.2114680	-1.6394492	-2.9201716
H	1.9716714	-3.0489731	0.2621767
H	2.9146937	-2.3555871	2.3748578
H	3.9730032	1.2516328	1.7890361

H	3.3843913	1.6711607	-0.5130678
C	-0.1693555	3.4204938	0.4750753
H	-1.3037903	0.9775366	1.4700371
H	0.0961062	4.4601487	0.2260643
O	-1.0299201	3.3129332	1.4604515
H	-1.1971164	2.2754735	1.5399009
H	0.8344693	1.1279183	2.6891617

TS3

42

Energy = -1015.2343663620

C	-1.2280367	-0.0415007	-1.6396463
C	-2.1197988	0.7551750	-0.8686484
C	1.2667949	-1.0167623	-0.4363016
C	-1.4469693	-1.4634865	-1.8931443
C	-3.3367024	0.2331002	-0.2432897
C	1.6532416	0.3417878	-0.6963817
C	1.9277413	-1.8927645	0.5224869
C	-1.9346123	-2.3464294	-0.9938840
C	2.7190982	1.0426634	0.0147679
C	-3.4565274	-0.9904857	0.3171357
C	2.4538511	-1.5107270	1.7022556
C	-2.2667876	-1.9352945	0.4262529
C	3.0606398	0.8502869	1.3049747
C	2.2975440	-0.0969301	2.1955268
C	-0.8634428	-1.4628290	2.4011954
C	0.0597213	-0.6882077	3.0426928
H	-0.6497151	0.4945312	-2.3963701
H	-2.1072024	1.8275360	-1.0733991
H	0.8180432	-1.5532635	-1.2694426
H	1.4630894	0.7165230	-1.7031360
H	-2.5621541	-2.8248955	1.0067380
H	2.6661840	-0.0197312	3.2308970
H	-1.4082835	-2.2350371	2.9571568
H	0.4158102	-0.8829369	4.0529606
N	-1.1147726	-1.3107152	1.0810354
N	0.8235081	0.2553300	2.2135910
O	0.1431744	2.4579811	-0.1325185
Ru	-0.2629446	0.3889631	0.3617356
H	-4.1938495	0.9113693	-0.2297420
H	-4.4185667	-1.3149686	0.7192846
H	-2.1065430	-3.3836677	-1.2855801
H	-1.1750187	-1.8251484	-2.8884167
H	1.9887690	-2.9458191	0.2391292
H	2.9790810	-2.2268595	2.3340551
H	3.9054205	1.3893251	1.7351913
H	3.2703628	1.7862911	-0.5647420
C	0.2226891	3.2880784	0.8491255
H	-1.7019078	1.0237386	1.3191340
H	0.4401507	4.3300943	0.5217678
O	0.0731788	3.0520590	2.0649809
H	-1.1083632	1.4510944	1.6732527
H	0.7377122	1.2338317	2.5419201

D
42
Energy = -1015.2345386520
C -1.2386421 -0.0559413 -1.6415173
C -2.1443532 0.7448499 -0.8955277
C 1.2629729 -0.9998931 -0.4397210
C -1.4458716 -1.4812558 -1.8838699
C -3.3588516 0.2179019 -0.2695969
C 1.6426198 0.3644076 -0.6881559
C 1.9254914 -1.8811231 0.5132704
C -1.9338191 -2.3594853 -0.9804373
C 2.7167214 1.0577049 0.0189358
C -3.4656383 -0.9986601 0.3091210
C 2.4519945 -1.5050355 1.6949408
C -2.2707679 -1.9363725 0.4347051
C 3.0627917 0.8558093 1.3056052
C 2.2957472 -0.0927055 2.1919927
C -0.8701997 -1.4458890 2.4075889
C 0.0535125 -0.6640450 3.0433061
H -0.6501468 0.4756372 -2.3933047
H -2.1349484 1.8147043 -1.1144919
H 0.8206088 -1.5326583 -1.2783828
H 1.4485170 0.7449060 -1.6922222
H -2.5670521 -2.8208838 1.0224401
H 2.6608603 -0.0171647 3.2287473
H -1.4170524 -2.2110316 2.9714089
H 0.4092636 -0.8550731 4.0546363
N -1.1204955 -1.3055052 1.0856020
N 0.8251245 0.2655883 2.2076580
O 0.1004311 2.4678917 -0.0776758
Ru -0.2688216 0.3928026 0.3697061
H -4.2235147 0.8866792 -0.2681160
H -4.4267924 -1.3266499 0.7106957
H -2.0976610 -3.4003599 -1.2635414
H -1.1632970 -1.8488863 -2.8739447
H 1.9873408 -2.9323526 0.2234104
H 2.9783165 -2.2238774 2.3228600
H 3.9092264 1.3900469 1.7380486
H 3.2684586 1.8014671 -0.5599498
C 0.3049246 3.2839819 0.9022174
H -1.7397230 1.1517303 1.1466510
H 0.4320817 4.3368805 0.5574670
O 0.3599597 3.0339031 2.1197458
H -1.2008555 1.2068205 1.7513382
H 0.7451457 1.2624356 2.5029582

E
40
Energy = -1014.0595423240
C -1.0839094 -0.3673108 -1.7246155
C -1.8652745 0.6647969 -1.1039852
C 1.4699515 -0.9994688 -0.5725043
C -1.5125970 -1.7609636 -1.7857514
C -3.1747253 0.4214106 -0.5026423

C 1.7749305 0.4046957 -0.8047868
C 2.2170258 -1.8307302 0.3725936
C -2.1753056 -2.4099428 -0.8037558
C 2.7406550 1.1717465 -0.0287660
C -3.5215761 -0.6858905 0.1868233
C 2.6796883 -1.4348901 1.5757220
C -2.4815241 -1.7427409 0.5229936
C 3.0338539 0.9931793 1.2773458
C 2.3476023 -0.0583459 2.1125104
C -0.9066783 -1.4611470 2.3925023
C 0.2120556 -0.8950434 2.8979816
H -0.4375583 -0.0423371 -2.5451319
H -1.6989631 1.6786437 -1.4717171
H 1.1338058 -1.5543726 -1.4492036
H 1.6179584 0.7649482 -1.8240581
H -2.9002087 -2.4787113 1.2267600
H 2.6751192 0.0254098 3.1620719
H -1.5015014 -2.1892209 2.9507736
H 0.6501064 -1.1510681 3.8621291
N -1.2865731 -1.1043159 1.1110574
N 0.8520412 0.0968440 2.0762676
O 0.0178925 2.3209185 0.0310840
Ru -0.1680748 0.1819416 0.2013804
H -3.9274391 1.1972411 -0.6623485
H -4.5496637 -0.8571222 0.5071676
H -2.5435108 -3.4254140 -0.9552752
H -1.2873272 -2.3058018 -2.7064528
H 2.4240329 -2.8529216 0.0451151
H 3.3132972 -2.0899897 2.1744927
H 3.7880321 1.6130038 1.7616925
H 3.2449691 1.9838145 -0.5583912
C 0.1344744 3.0483359 1.0859530
H 0.0562861 4.1360823 0.8613271
O 0.3078457 2.6982734 2.2725703
H 0.5887066 1.1172899 2.3361484

TS4

40

Energy = -1014.0303549010

C -1.0670721 -0.0203534 -1.7450093
C -1.8928873 0.8810857 -1.0031273
C 1.3963551 -1.1181057 -0.5123544
C -1.3976245 -1.4315751 -1.9321336
C -3.1348416 0.5041675 -0.3318438
C 1.8359567 0.2138777 -0.8166288
C 1.9776423 -1.9697362 0.5088585
C -1.9549841 -2.2237681 -0.9918834
C 2.9199555 0.9010765 -0.1244344
C -3.3506536 -0.6794925 0.2812194
C 2.5017235 -1.5556701 1.6809941
C -2.2425198 -1.7106654 0.4036501
C 3.2486547 0.7464931 1.1766545
C 2.4376893 -0.1170924 2.1039964
C -0.8210596 -1.2356699 2.3505679

C	0.1396107	-0.4965409	2.9713339
H	-0.4784645	0.4325900	-2.5477925
H	-1.8236997	1.9312958	-1.2963286
H	0.9251931	-1.6644182	-1.3270120
H	1.6742663	0.5464431	-1.8441678
H	-2.5900430	-2.5449923	1.0347098
H	2.8105222	-0.0179982	3.1356700
H	-1.4373420	-1.9301655	2.9336174
H	0.4426236	-0.6299611	4.0080402
N	-1.0335697	-1.1507161	1.0178115
N	0.9755662	0.3459673	2.1089825
O	0.3085322	2.6461734	-0.1410293
Ru	-0.0818452	0.4475502	0.2094722
H	-3.9201825	1.2641891	-0.3179853
H	-4.3208572	-0.8948473	0.7334354
H	-2.2139973	-3.2569318	-1.2284436
H	-1.1613049	-1.8597953	-2.9101565
H	1.9690540	-3.0412663	0.2967154
H	2.9497747	-2.2731375	2.3679199
H	4.1166775	1.2650971	1.5858502
H	3.5036274	1.6007140	-0.7268915
C	-0.3218794	2.6845968	0.9327150
H	-1.0902673	1.5323429	1.1011948
O	-0.5376833	3.3927004	1.8847235
H	0.9972738	1.3073659	2.4741669

F'

40

Energy = -1014.0630406170

C	-1.0623813	-0.8033360	-1.7294761
C	-1.6587651	0.4232780	-1.2601829
C	1.7271084	-0.7099551	-0.8311653
C	-1.7204146	-2.1032048	-1.6237349
C	-2.9893974	0.4746193	-0.6519836
C	1.7225465	0.7433610	-0.7703451
C	2.5017609	-1.5894855	0.0425076
C	-2.5114946	-2.5043986	-0.6023908
C	2.5608606	1.4845110	0.1644059
C	-3.5172342	-0.4618714	0.1668585
C	2.8174271	-1.3739739	1.3403047
C	-2.6961527	-1.6489830	0.6423716
C	2.8971664	1.1101543	1.4235716
C	2.3520023	-0.1385577	2.0734244
C	-0.9983184	-1.5488631	2.4413284
C	0.1797193	-1.0751602	2.9175012
H	-0.3953178	-0.6972208	-2.5903874
H	-1.3759285	1.3214091	-1.8137087
H	1.5613573	-1.1297921	-1.8266879
H	1.5455976	1.2496618	-1.7228229
H	-3.2541840	-2.2109202	1.4086602
H	2.6845013	-0.1838490	3.1237514
H	-1.6353272	-2.2036792	3.0463556
H	0.5665092	-1.2474240	3.9203382
N	-1.3979953	-1.2155342	1.1684247

N 0.8444291 -0.0972030 2.0565790
Ru -0.0988403 -0.1792403 0.1286994
H -3.6078365 1.3355444 -0.9203306
H -4.5592486 -0.3989842 0.4841612
H -3.0526670 -3.4500835 -0.6575836
H -1.5718638 -2.7888651 -2.4619997
H 2.8362074 -2.5265636 -0.4116121
H 3.4575209 -2.0768149 1.8737405
H 3.5744206 1.7299844 2.0122225
H 2.9591748 2.4362809 -0.2000437
C 0.4033908 3.5583408 1.4552738
O 0.2704735 3.0721839 2.5150369
H 0.5741064 0.8485013 2.3668372
H -0.2271483 1.3761906 0.2491070
O 0.5295777 4.0731291 0.4129996

F

37

Energy = -825.3668835170

C -1.0602189 -0.8155785 -1.7294996
C -1.6514293 0.4154522 -1.2637753
C 1.7339868 -0.7014237 -0.8402138
C -1.7228603 -2.1126886 -1.6183914
C -2.9828146 0.4739435 -0.6575789
C 1.7213036 0.7508523 -0.7657882
C 2.5076555 -1.5867973 0.0280322
C -2.5159416 -2.5071552 -0.5958441
C 2.5486522 1.4925580 0.1782625
C -3.5153479 -0.4573263 0.1641306
C 2.8208675 -1.3806823 1.3279842
C -2.6994604 -1.6458501 0.6450445
C 2.8971990 1.1064509 1.4305468
C 2.3587870 -0.1495165 2.0697301
C -1.0040222 -1.5394744 2.4456778
C 0.1751848 -1.0664378 2.9223144
H -0.3938554 -0.7156077 -2.5917216
H -1.3655568 1.3103213 -1.8211845
H 1.5716768 -1.1129066 -1.8399131
H 1.5428975 1.2626734 -1.7153234
H -3.2606240 -2.2028169 1.4127927
H 2.6920703 -0.2038573 3.1194943
H -1.6449827 -2.1863112 3.0554697
H 0.5594052 -1.2350109 3.9268197
N -1.4003966 -1.2159708 1.1707282
N 0.8498045 -0.1028106 2.0541280
Ru -0.0947574 -0.1901623 0.1247880
H -3.5974743 1.3365063 -0.9293179
H -4.5571219 -0.3880748 0.4808546
H -3.0589446 -3.4519960 -0.6472868
H -1.5757825 -2.8026827 -2.4533606
H 2.8424951 -2.5210349 -0.4318592
H 3.4582177 -2.0889247 1.8576436
H 3.5662102 1.7292886 2.0254327
H 2.9303644 2.4563969 -0.1729200

H 0.5861832 0.8525908 2.3408217
H -0.2294694 1.3635967 0.2699771

CO_2
3
Energy = -188.6877070571
C 1.8675480 -0.8928571 0.0000000
O 3.0390341 -0.8928571 0.0000000
O 0.6960968 -0.8928571 0.0000000

methanediol
7
Energy = -191.0471789649
C -0.2275480 0.5926530 -0.0023092
H 0.3328163 -0.3361629 0.1603639
H 0.1229586 1.0838924 -0.9315960
O 0.0443451 1.4053459 1.1272293
H -0.2535603 2.3120339 0.9389724
O -1.5862643 0.2049085 -0.1233351
H -2.0915624 0.9476639 -0.4962822

4’*m*
39
Energy = -826.5822371755
C -1.2585634 -0.2219873 -1.3959244
C -1.5579403 1.2034402 -1.4907688
C 1.6226686 0.7113321 -0.7994444
C -2.2554650 -1.2165417 -0.9993349
C -2.8195633 1.7930331 -1.0586080
C 1.3142796 2.1012819 -0.5480005
C 2.6078840 -0.0264129 -0.0147121
C -3.2153142 -1.0672937 -0.0540909
C 2.0087125 2.8995105 0.4642820
C -3.5983563 1.3792520 -0.0283126
C 2.8457069 0.1469667 1.3068781
C -3.2694990 0.1699792 0.8108240
C 2.3781604 2.4623892 1.6883746
C 1.9928372 1.0624933 2.1638118
C -1.7643378 1.1286849 2.6717065
C -0.2907988 1.0770158 3.1311137
H -0.5495838 -0.5888707 -2.1445562
H -1.0690803 1.7310492 -2.3155287
H 1.5102967 0.3803592 -1.8369024
H 0.9793407 2.6694726 -1.4193889
H -4.0275693 0.0470382 1.6022536
H 2.2701336 0.9429530 3.2252319
H -2.0428557 2.1456771 2.3692994
H -0.1566092 0.3019751 3.9140193
N -1.9033442 0.2694673 1.4641847
N 0.5663720 0.7844397 1.9811418
Ru -0.2727841 0.8291095 0.2305290
H -3.1415638 2.6886383 -1.5994479
H -4.5266329 1.9066681 0.1942758
H -3.9592048 -1.8471906 0.1116646

H	-2.2217465	-2.1689641	-1.5379596
H	3.2194199	-0.7503698	-0.5602329
H	3.6844107	-0.3618299	1.7846472
H	2.9945075	3.0843468	2.3400678
H	2.2670758	3.9221276	0.1762008
H	-0.0373086	2.0464799	3.6041254
H	-0.7543514	2.3087593	0.3388487
H	-1.6668575	-0.6962853	1.7300182
H	-2.4501086	0.7999595	3.4733493

4-A

46

Energy = -1017.6632452460

C	-1.2227828	-0.3318569	-1.2341248
C	-1.5901883	1.0728089	-1.2741417
C	1.6771745	0.7694566	-0.8650763
C	-2.1208798	-1.3764587	-0.7442227
C	-2.8444434	1.5926642	-0.7386763
C	1.2650134	2.1243235	-0.5920397
C	2.7706409	0.1193590	-0.1531423
C	-2.9898183	-1.2730658	0.2906759
C	1.9453082	2.9947520	0.3675556
C	-3.5076374	1.1334180	0.3503750
C	3.1095862	0.3325506	1.1413017
C	-3.0263386	-0.0417488	1.1664861
C	2.4503480	2.6086937	1.5604689
C	2.2668628	1.1843250	2.0657680
C	-1.4565208	1.0552635	2.8582241
C	0.0551003	1.1861581	3.1804634
H	-0.5582901	-0.6588233	-2.0401226
H	-1.1886741	1.6392942	-2.1202357
H	1.5123486	0.4185784	-1.8892047
H	0.8110414	2.6525285	-1.4342527
H	-3.7059328	-0.2081564	2.0186893
H	2.6555640	1.1050986	3.0962383
H	-1.8752914	2.0277371	2.5697527
H	0.3153693	0.5920569	4.0783364
N	-1.6252672	0.1396114	1.6980009
N	0.8600652	0.7362265	2.0307360
Ru	-0.1415965	0.7323621	0.3185488
H	-3.2675790	2.4581664	-1.2572239
H	-4.4550552	1.5907357	0.6385920
H	-3.6720561	-2.0909312	0.5245373
H	-2.0939094	-2.3245589	-1.2909605
H	3.3736351	-0.5859262	-0.7311362
H	4.0020347	-0.1374480	1.5551691
H	3.0376089	3.3042477	2.1629127
H	2.0650689	4.0389277	0.0659513
H	0.2722313	2.2424397	3.4258764
H	-0.6870295	2.1743921	0.5089401
H	-1.3491218	-0.8116007	2.0176865
H	-2.0203375	0.6789925	3.7300276
H	1.1078988	-1.0231544	2.1650631
O	1.2655301	-2.0188509	2.2798093

C 0.3454521 -2.5183977 3.1882759
H 0.6456605 -3.5509032 3.4324388
H 0.2729555 -1.9219812 4.1155456
O -1.0122608 -2.5163529 2.6781898
H -0.9926512 -2.9825063 1.8198691

4-B'
46
Energy = -1017.6704772250
C -1.1509878 -0.6386856 -0.9433902
C -1.4295281 0.7862369 -1.0430226
C 1.8896524 0.3900559 -0.4995981
C -2.1609856 -1.5664138 -0.4352016
C -2.6837932 1.4076484 -0.6078267
C 1.3440405 1.7270543 -0.5665959
C 3.0015213 0.0363614 0.3774512
C -3.0157166 -1.3321082 0.5895674
C 1.8705830 2.8642687 0.1940055
C -3.4008177 1.0736424 0.4873748
C 3.2589656 0.5578350 1.5978551
C -2.9779212 -0.0520549 1.3997372
C 2.3292832 2.8287381 1.4633552
C 2.3348850 1.5505713 2.2658214
C -1.4734552 1.2410191 2.9496012
C 0.0087887 1.5898713 3.2742332
H -0.5062867 -1.0510165 -1.7246257
H -0.9775386 1.2839894 -1.9055395
H 1.8200374 -0.2017401 -1.4159991
H 0.9287948 2.0126013 -1.5363372
H -3.6786533 -0.1296617 2.2481068
H 2.6844546 1.7534935 3.2919246
H -1.9680886 2.1176780 2.5113633
H 0.2601209 1.3135737 4.3114858
N -1.5851139 0.1482628 1.9470686
N 0.9605370 0.9325277 2.3410826
Ru -0.0234158 0.4567184 0.5439835
H -3.0470548 2.2344739 -1.2252804
H -4.3372645 1.5877611 0.7077202
H -3.8104559 -2.0459056 0.8143553
H -2.2514365 -2.5208753 -0.9620707
H 3.6811247 -0.7347525 0.0058073
H 4.1483488 0.2489699 2.1478451
H 2.7465476 3.7260287 1.9223553
H 1.8791802 3.8246059 -0.3295441
H 0.1493573 2.6742908 3.1706093
H -0.5202223 1.9565755 0.6022471
H -1.3077336 -0.7604759 2.3769856
H -2.0157177 0.9677886 3.8718976
H 1.0851840 -0.0796944 2.5747294
O 0.7159033 -1.4240110 1.4407559
C 0.1344808 -2.6387613 1.5420467
H -0.2489831 -3.0307885 0.5753951
H 0.8475790 -3.3701552 1.9701483
O -1.0167870 -2.6499487 2.4872726

H -1.8125759 -2.7331453 1.9267895

4-B

46

Energy = -1017.6710979250

C -1.2710979 -0.6204725 -0.8393198
C -1.5160654 0.8043961 -0.9885429
C 1.7986302 0.1598112 -0.3529387
C -2.2640067 -1.5197036 -0.2535756
C -2.7683386 1.4562947 -0.5903370
C 1.3712125 1.5281793 -0.5658988
C 2.8638809 -0.1994632 0.5815474
C -3.1056245 -1.2319218 0.7677438
C 1.9810434 2.6824050 0.1029257
C -3.4971050 1.1759833 0.5109615
C 3.1440551 0.4094017 1.7556574
C -3.0792391 0.1009153 1.4863608
C 2.4023074 2.7276577 1.3847733
C 2.2913750 1.5332598 2.3017518
C -1.5447700 1.4288210 2.9998831
C -0.0533524 1.8294643 3.2269441
H -0.6433723 -1.0773753 -1.6102726
H -1.0491497 1.2628584 -1.8642007
H 1.7032574 -0.5079250 -1.2138422
H 1.0091517 1.7499594 -1.5735089
H -3.7767375 0.0773077 2.3404334
H 2.6348064 1.8050912 3.3138307
H -2.0923803 2.2898328 2.5944846
H 0.2173641 1.7387667 4.2925772
N -1.6857426 0.3325774 2.0075314
N 0.8750536 1.0221552 2.3960372
Ru -0.1130521 0.4912312 0.6119127
H -3.1259024 2.2494400 -1.2535906
H -4.4393253 1.6944733 0.6928450
H -3.8555638 -1.9592360 1.0811579
H -2.3324665 -2.5157586 -0.7014708
H 3.4855870 -1.0520655 0.2938250
H 3.9966902 0.0843007 2.3525244
H 2.8823178 3.6266794 1.7731173
H 2.0869524 3.5857931 -0.5044201
H 0.0893255 2.8768380 2.9295316
H -0.4971748 2.0303601 0.5457214
H -1.3361054 -0.5724582 2.3863569
H -2.0213485 1.1395785 3.9519900
H 0.9298049 0.0355506 2.7285964
O 0.2407478 -1.3535215 1.7040372
C 0.5090751 -2.6143157 1.2068700
H 0.5496826 -2.5990495 0.0958562
H 1.4720880 -3.0078097 1.5872679
O -0.4635163 -3.5793406 1.6413338
H -1.3241356 -3.1865188 1.4014769

4-TS1

46

Energy = -1017.6640763610
C -1.2638163 -0.5742261 -0.9768336
C -1.5622766 0.8485635 -1.0707561
C 1.7718586 0.2020948 -0.5142382
C -2.2028222 -1.5381064 -0.3979946
C -2.8180538 1.4412868 -0.5976935
C 1.3770657 1.5979920 -0.6269391
C 2.8428647 -0.2502092 0.3789294
C -3.0249633 -1.3296530 0.6580099
C 2.0099858 2.6794553 0.1352167
C -3.5089474 1.0832187 0.5054784
C 3.1371245 0.2461187 1.6018782
C -3.0367836 -0.0220461 1.4197869
C 2.4492467 2.6133720 1.4105860
C 2.3255461 1.3552929 2.2353287
C -1.5105693 1.3466385 2.9091677
C -0.0198762 1.7446348 3.1256844
H -0.6488981 -0.9809427 -1.7845943
H -1.1386420 1.3535014 -1.9430915
H 1.6735962 -0.3891535 -1.4286805
H 1.0257022 1.9037357 -1.6160858
H -3.7128413 -0.1024367 2.2877655
H 2.6889271 1.5443569 3.2595568
H -2.0644787 2.2000360 2.4982272
H 0.2524267 1.6769564 4.1928106
N -1.6355414 0.2347335 1.9248864
N 0.8889740 0.8860775 2.3155690
Ru -0.1146948 0.5160694 0.4772915
H -3.2134711 2.2630562 -1.2023372
H -4.4544896 1.5717794 0.7435775
H -3.7392930 -2.0982708 0.9580343
H -2.2366410 -2.5184932 -0.8819574
H 3.4591376 -1.0741424 0.0054459
H 3.9899354 -0.1414210 2.1603780
H 2.9346796 3.4759990 1.8681330
H 2.1152619 3.6330026 -0.3909804
H 0.1411620 2.7764602 2.7896282
H -0.5394940 2.0905431 0.5877821
H -1.3321519 -0.6355861 2.4059879
H -1.9796191 1.0512411 3.8632952
H 0.9041898 -0.0488601 2.7562482
O -0.0765671 -1.8466641 3.0802184
C 0.3706963 -2.3726041 2.0411249
H 0.2647240 -1.1102123 0.8454069
H 1.4448902 -2.4018059 1.7898021
O -0.3016796 -3.3395434 1.3584347
H -1.2525471 -3.2293922 1.5784572

4-C'

46

Energy = -1017.6642869090
C -1.2424423 -0.5675339 -0.9890094
C -1.5506751 0.8586912 -1.0705877
C 1.7888322 0.2349032 -0.5331994

C	-2.1816675	-1.5370647	-0.4206234
C	-2.8087351	1.4418901	-0.5908873
C	1.3731097	1.6268001	-0.6430042
C	2.8760270	-0.2001770	0.3517156
C	-3.0031681	-1.3429365	0.6404480
C	1.9887371	2.7183703	0.1197433
C	-3.4985294	1.0727161	0.5096758
C	3.1730238	0.3009059	1.5714588
C	-3.0209737	-0.0395194	1.4111255
C	2.4339373	2.6584344	1.3929657
C	2.3454520	1.3941990	2.2108137
C	-1.4918275	1.3262702	2.9017569
C	-0.0013883	1.7220647	3.1261836
H	-0.6361639	-0.9644280	-1.8077192
H	-1.1376135	1.3690021	-1.9451020
H	1.6986387	-0.3547413	-1.4496113
H	1.0177595	1.9280778	-1.6322822
H	-3.6936566	-0.1306940	2.2807521
H	2.7086591	1.5868465	3.2345381
H	-2.0412605	2.1810409	2.4880426
H	0.2711584	1.6341608	4.1919374
N	-1.6175460	0.2174221	1.9146163
N	0.9172355	0.8907929	2.2981534
Ru	-0.0970120	0.5218183	0.4601879
H	-3.2047523	2.2705638	-1.1856162
H	-4.4408961	1.5616542	0.7580687
H	-3.7309457	-2.1078255	0.9191404
H	-2.2220673	-2.5099622	-0.9202912
H	3.5025007	-1.0130604	-0.0286346
H	4.0368044	-0.0712394	2.1236720
H	2.8957611	3.5319393	1.8541136
H	2.0690022	3.6760454	-0.4032404
H	0.1539078	2.7613561	2.8109601
H	-0.5401661	2.1020424	0.6135653
H	-1.3234928	-0.6514755	2.3925262
H	-1.9659141	1.0319912	3.8540702
H	0.9692733	-0.0456175	2.7233687
O	-0.1403749	-1.9133797	3.1746699
C	0.2786350	-2.5755345	2.2252695
H	0.3139274	-1.0702708	0.7914896
H	1.3480427	-2.6983683	1.9837472
O	-0.4750029	-3.3888748	1.4660970
H	-1.4153167	-3.1048484	1.5608809

4-C

46

Energy = -1017.6765450530

C	-1.3479144	-0.4608092	-1.1239671
C	-1.6205566	0.9649757	-1.1161722
C	1.7286451	0.3014423	-0.6648754
C	-2.2812812	-1.4520760	-0.5786329
C	-2.8563391	1.5509126	-0.5843874
C	1.3054444	1.6898588	-0.6797862
C	2.8206232	-0.1884900	0.1829059

C -3.0847288 -1.2974811 0.4967613
C 1.9359807 2.7381256 0.1306177
C -3.5352391 1.1345535 0.5050444
C 3.1333437 0.2426339 1.4247602
C -3.0728530 -0.0380999 1.3339392
C 2.4006270 2.6049356 1.3906244
C 2.3173187 1.2972757 2.1381639
C -1.5052984 1.2213440 2.8789453
C -0.0098601 1.5923183 3.1019447
H -0.7475046 -0.8225523 -1.9629027
H -1.1962791 1.5236395 -1.9548077
H 1.6175750 -0.2364613 -1.6100572
H 0.9223356 2.0483445 -1.6390133
H -3.7377416 -0.1626411 2.2051145
H 2.6965811 1.4313134 3.1652685
H -2.0516502 2.1001277 2.5139390
H 0.2765665 1.4411083 4.1560603
N -1.6589725 0.1644236 1.8391199
N 0.8898291 0.7962882 2.2194396
Ru -0.1476380 0.5134258 0.3854500
H -3.2415829 2.4197725 -1.1262019
H -4.4621713 1.6301009 0.7957035
H -3.7710248 -2.0936962 0.7870726
H -2.3152256 -2.4116583 -1.1030232
H 3.4319364 -0.9887546 -0.2447615
H 3.9971836 -0.1701814 1.9468732
H 2.8777683 3.4482957 1.8908934
H 2.0136366 3.7217177 -0.3419614
H 0.1503231 2.6461713 2.8419885
H -0.5658455 2.0725726 0.6006387
H -1.3674844 -0.7255542 2.2693702
H -1.9682579 0.8840545 3.8212897
H 0.9317826 -0.1553894 2.6128737
O -0.0590324 -1.7374325 3.5405147
C 0.2783528 -2.8161410 3.0487975
H 0.2356926 -1.1497796 0.5739771
H 0.4090819 -3.7245352 3.6725734
O 0.5246961 -3.0590325 1.7841401
H 0.3879939 -2.1565198 1.2116908

4-TS2

46
Energy = -1017.6735939860
C -1.3922475 -0.4427805 -1.1685983
C -1.6350048 0.9787861 -1.1191293
C 1.7345228 0.2903415 -0.7019861
C -2.3342347 -1.4318728 -0.6370843
C -2.8588009 1.5735999 -0.5703800
C 1.3012483 1.6676593 -0.6845877
C 2.8205521 -0.2182807 0.1361840
C -3.1223963 -1.2850805 0.4499152
C 1.9277810 2.7041045 0.1418710
C -3.5361697 1.1406495 0.5127930
C 3.1352721 0.1914782 1.3846136

C	-3.0755309	-0.0492005	1.3196058
C	2.3973598	2.5470643	1.3972007
C	2.3199596	1.2270613	2.1224916
C	-1.5009156	1.1652953	2.8713596
C	-0.0005984	1.4939276	3.1172343
H	-0.7886230	-0.7940226	-2.0098798
H	-1.1909437	1.5570305	-1.9336154
H	1.6071174	-0.2370328	-1.6510702
H	0.8973796	2.0421449	-1.6285592
H	-3.7342706	-0.1845817	2.1933495
H	2.7042026	1.3441906	3.1492184
H	-2.0236108	2.0655263	2.5227850
H	0.2780572	1.2932010	4.1639754
N	-1.6615058	0.1243676	1.8173716
N	0.8924102	0.7175479	2.2063464
Ru	-0.1536320	0.4654526	0.3806124
H	-3.2370649	2.4542515	-1.0970973
H	-4.4624486	1.6330789	0.8116808
H	-3.8158918	-2.0771564	0.7329330
H	-2.3854600	-2.3795308	-1.1802794
H	3.4173013	-1.0260157	-0.2959834
H	3.9874817	-0.2466221	1.9036573
H	2.8808901	3.3802345	1.9084361
H	2.0021145	3.6957823	-0.3136646
H	0.1849707	2.5539909	2.9025098
H	-0.5509670	2.0014323	0.5847679
H	-1.3809215	-0.7740198	2.2769498
H	-1.9764373	0.8159209	3.8021767
H	0.9636739	-0.2409366	2.5748162
O	-0.6276658	-1.8337089	3.4957259
C	0.3203261	-2.5894510	3.1945649
H	0.1630720	-1.3093884	0.4224964
H	0.6043953	-3.4023668	3.9030512
O	1.0554267	-2.5396408	2.1404398
H	0.5286633	-1.7299836	1.1867270

4-D'-H2

46

Energy = -1017.6809482200

C	-1.4320820	-0.4498020	-1.2061117
C	-1.6445937	0.9654794	-1.1142405
C	1.7807692	0.3291475	-0.7582804
C	-2.3601194	-1.4396667	-0.6612711
C	-2.8466369	1.5664208	-0.5280943
C	1.3146482	1.6849380	-0.7039349
C	2.8742119	-0.1799859	0.0661290
C	-3.1101095	-1.3008727	0.4536037
C	1.9310206	2.7205679	0.1304575
C	-3.5043278	1.1162099	0.5602146
C	3.1776364	0.2124729	1.3228613
C	-3.0254612	-0.0826460	1.3432246
C	2.4176999	2.5453866	1.3765971
C	2.3402881	1.2136443	2.0842044
C	-1.4414713	1.0922611	2.8922644

C 0.0550310 1.4515945 3.1049496
H -0.8415753 -0.7872112 -2.0626801
H -1.1961826 1.5586512 -1.9153452
H 1.6417135 -0.1850112 -1.7136986
H 0.8710693 2.0656013 -1.6272566
H -3.6642316 -0.2309418 2.2290029
H 2.7290712 1.3126387 3.1106742
H -1.9976537 1.9907501 2.5913792
H 0.3664716 1.2367382 4.1404118
N -1.6043386 0.0737321 1.8167484
N 0.9292341 0.6928827 2.1682223
Ru -0.1240450 0.4309371 0.3769195
H -3.2260253 2.4592528 -1.0322870
H -4.4245801 1.6052807 0.8826896
H -3.7920386 -2.0964604 0.7523533
H -2.4295579 -2.3822783 -1.2109225
H 3.4870971 -0.9652925 -0.3846150
H 4.0368019 -0.2196178 1.8348112
H 2.9092116 3.3719906 1.8915756
H 1.9903403 3.7177257 -0.3145077
H 0.2092076 2.5219930 2.9125294
H -0.5173369 1.9386284 0.5688298
H -1.3517784 -0.8742689 2.2708718
H -1.8858767 0.7020560 3.8223834
H 1.0112781 -0.3084455 2.5568581
O -1.0891146 -2.2065553 3.1033611
C 0.1062176 -2.4576630 3.4284938
H 0.2174515 -1.4684785 0.1117085
H 0.2688723 -3.3805978 4.0393577
O 1.1454730 -1.7922378 3.1401733
H 0.3771580 -1.4365011 0.8853301

4-D'

44

Energy = -1016.4921840250

C -1.3516986 -0.3683652 -1.1990498
C -1.6875160 1.0277689 -1.1345173
C 1.6727890 0.3514059 -0.7812114
C -2.1814462 -1.4286504 -0.6384648
C -2.9231513 1.5363959 -0.5439690
C 1.3258851 1.7448828 -0.7184001
C 2.7111804 -0.2639445 0.0372790
C -2.9774293 -1.3396741 0.4552361
C 2.0097195 2.7113333 0.1372087
C -3.5521897 1.0370762 0.5448425
C 3.0746046 0.1016115 1.2911676
C -2.9972766 -0.1188740 1.3428434
C 2.4920149 2.4770015 1.3792241
C 2.3228586 1.1485385 2.0773736
C -1.4546943 1.1245326 2.9058523
C 0.0459604 1.4818301 3.1133009
H -0.7335465 -0.6695441 -2.0506953
H -1.2865699 1.6460465 -1.9428212
H 1.4943206 -0.1399679 -1.7430907
H 0.9138611 2.1691117 -1.6380022

H	-3.6344753	-0.3099685	2.2215662
H	2.7291268	1.2061245	3.1000154
H	-2.0230556	2.0214669	2.6239093
H	0.3652078	1.2455732	4.1413776
N	-1.5929233	0.1221337	1.8131125
N	0.8888032	0.7131202	2.1556138
Ru	-0.1639517	0.5661873	0.3662575
H	-3.3716502	2.4001620	-1.0424104
H	-4.5124089	1.4521546	0.8539819
H	-3.5961893	-2.1873458	0.7475251
H	-2.1514103	-2.3867614	-1.1643269
H	3.2448898	-1.1015895	-0.4195298
H	3.9075072	-0.3990767	1.7833569
H	3.0532355	3.2554781	1.8979989
H	2.1390185	3.7135586	-0.2812803
H	0.2097438	2.5528437	2.9335135
H	-0.5400641	2.0595199	0.5153005
H	-1.2909993	-0.8290258	2.2522484
H	-1.8896850	0.7109901	3.8301880
H	0.9404542	-0.2954673	2.5663919
O	-1.0537528	-2.1661760	3.0437915
C	0.1135804	-2.3737655	3.4869105
H	0.2657455	-3.3343153	4.0395669
O	1.1298045	-1.6269095	3.3797217

4-D

44

Energy = -1016.5078739110

C	-1.3904614	-0.6078851	-0.9514098
C	-1.6183430	0.8132303	-1.0058360
C	1.7288348	0.1353443	-0.5421424
C	-2.3287428	-1.5458436	-0.3389806
C	-2.8333311	1.4608515	-0.5045533
C	1.3109569	1.5119602	-0.6169030
C	2.8033810	-0.3275527	0.3328295
C	-3.1241658	-1.3102951	0.7290554
C	1.9525770	2.5970372	0.1302434
C	-3.5193773	1.1157921	0.6057881
C	3.1238671	0.1684383	1.5492922
C	-3.0764985	-0.0134753	1.5039643
C	2.4266765	2.5228620	1.3925112
C	2.3207451	1.2618599	2.2155958
C	-1.4922125	1.3125772	2.9607662
C	0.0107245	1.6685253	3.1577016
H	-0.7745391	-1.0310207	-1.7491921
H	-1.1656087	1.3279903	-1.8574090
H	1.5792764	-0.4662033	-1.4422306
H	0.8989420	1.8259207	-1.5795212
H	-3.7461130	-0.0759036	2.3780856
H	2.7126182	1.4471196	3.2298438
H	-2.0238517	2.1780086	2.5446485
H	0.2960695	1.5855465	4.2193863
N	-1.6661148	0.1914800	1.9953604
N	0.8915434	0.7955654	2.3323227
Ru	-0.1461705	0.3833373	0.5347698

H	-3.2021170	2.3055606	-1.0937694
H	-4.4416491	1.6393117	0.8618519
H	-3.8195492	-2.0774695	1.0706109
H	-2.3749135	-2.5383513	-0.7941139
H	3.3941797	-1.1675025	-0.0416335
H	3.9802563	-0.2316677	2.0926847
H	2.9309283	3.3815661	1.8375994
H	2.0426023	3.5514000	-0.3966522
H	0.1904034	2.7013192	2.8325072
H	-0.5169494	1.9122924	0.5902325
H	-1.3570478	-0.6754545	2.4797843
H	-1.9606449	1.0487208	3.9234919
H	0.8869537	-0.1452376	2.7750363
O	0.3245550	-1.8243852	0.9468397
C	0.2941191	-2.3496947	2.1046289
H	0.5490330	-3.4362780	2.1290545
O	0.0133850	-1.7819707	3.1967670

4-E

44

Energy = -1016.4857813490

C	-1.3617330	-0.4773230	-1.0864485
C	-1.6503318	0.9355731	-1.0779481
C	1.7222850	0.2762290	-0.6895420
C	-2.2556801	-1.4829824	-0.5091617
C	-2.8727756	1.5093919	-0.5095676
C	1.3282204	1.6638224	-0.6920876
C	2.8090377	-0.2479358	0.1396612
C	-3.0389393	-1.3336049	0.5823661
C	1.9829168	2.6933914	0.1189095
C	-3.5159042	1.0832022	0.5989200
C	3.1529943	0.1732406	1.3771706
C	-3.0179116	-0.0790043	1.4243472
C	2.4647931	2.5384810	1.3711487
C	2.3651423	1.2292589	2.1174654
C	-1.4326288	1.1830852	2.9462646
C	0.0686200	1.5476257	3.1392325
H	-0.7533723	-0.8368935	-1.9207773
H	-1.2382762	1.5049315	-1.9152750
H	1.5620600	-0.2687835	-1.6236912
H	0.9153861	2.0345089	-1.6336979
H	-3.6631188	-0.2105695	2.3086452
H	2.7678217	1.3503409	3.1365977
H	-1.9893548	2.0670947	2.6100084
H	0.3826777	1.3820016	4.1829927
N	-1.5989887	0.1398728	1.8953034
N	0.9342119	0.7549541	2.2209656
Ru	-0.1358391	0.5064820	0.4185854
H	-3.2872192	2.3700040	-1.0419660
H	-4.4499267	1.5572517	0.9029811
H	-3.6943101	-2.1451985	0.8981860
H	-2.2662883	-2.4524097	-1.0133175
H	3.3789379	-1.0739199	-0.2928303
H	4.0096373	-0.2693575	1.8851956
H	2.9805084	3.3643969	1.8624144

H 2.0757252 3.6787763 -0.3466740
H 0.2285270 2.6053762 2.8948789
H -0.5288434 2.0367424 0.5690198
H -1.2551017 -0.7616775 2.2882625
H -1.8773026 0.8330198 3.8922803
H 0.9525218 -0.2259118 2.5720572
H 0.2768380 -1.3233509 0.6644738
C 0.4168336 -2.3052487 1.4142931
O 0.1948950 -2.0143864 2.6139025
O 0.7328758 -3.2910493 0.7738609

4-TS3

44

Energy = -1016.4763281430

C -1.3567099 -0.4790672 -1.0713002
C -1.6328467 0.9507495 -1.0758633
C 1.7065673 0.2618575 -0.6756789
C -2.2930082 -1.4640499 -0.5172700
C -2.8519163 1.5474410 -0.5198749
C 1.3035363 1.6613266 -0.6949579
C 2.8213096 -0.2296386 0.1423807
C -3.0879742 -1.3024543 0.5640342
C 1.9525842 2.7075797 0.1020878
C -3.5156575 1.1405174 0.5830395
C 3.1730523 0.2067657 1.3722317
C -3.0568791 -0.0409419 1.3979038
C 2.4431563 2.5773785 1.3531971
C 2.3806907 1.2700795 2.1003203
C -1.4337998 1.1676615 2.9411398
C 0.0699133 1.5332666 3.1335359
H -0.7747237 -0.8471826 -1.9205752
H -1.2276602 1.4983672 -1.9309670
H 1.5745530 -0.2776751 -1.6173624
H 0.9134417 2.0186191 -1.6515450
H -3.7070215 -0.1596374 2.2812525
H 2.7817497 1.4031460 3.1193816
H -1.9837982 2.0563190 2.6065541
H 0.3825010 1.3720847 4.1798876
N -1.6256482 0.1390038 1.8807323
N 0.9515529 0.7617530 2.2132669
Ru -0.1387698 0.5098090 0.3990013
H -3.2351244 2.4240888 -1.0505552
H -4.4260054 1.6528842 0.8960617
H -3.7803199 -2.0909714 0.8606368
H -2.3405478 -2.4222834 -1.0430117
H 3.4178803 -1.0325632 -0.3008235
H 4.0510955 -0.2041638 1.8713904
H 2.9234850 3.4235588 1.8454201
H 2.0164491 3.6923845 -0.3705282
H 0.2217410 2.5912559 2.8860913
H -0.5522756 2.0828622 0.6550136
H -1.3490532 -0.7776528 2.2520400
H -1.8783845 0.8198938 3.8896439
H 1.0347571 -0.1998806 2.5635896
H 0.2234050 -1.1203999 0.6199578

C 0.5609028 -2.7946600 1.6810036
O 0.3092484 -2.3308157 2.7438322
O 0.8541725 -3.4771679 0.7730908

4-TS3"

44

Energy = -1016.4350040380

C -1.2547608 -0.2094539 -1.5281767
C -2.0962737 0.8600261 -0.9994078
C 2.7296005 -1.0285689 -1.2124393
C -1.6335935 -1.6262319 -1.5068777
C -3.4125457 0.6181463 -0.4101859
C 2.9697337 0.3118062 -1.2720788
C 2.7728207 -1.8597748 -0.0397479
C -2.2906699 -2.2503526 -0.5073940
C 3.3280294 1.1728454 -0.1788744
C -3.7597178 -0.4488398 0.3439063
C 2.7025234 -1.5017894 1.2671457
C -2.7475329 -1.5072060 0.7191955
C 3.1815358 0.9725810 1.1520811
C 2.4471508 -0.1395979 1.8417283
C -0.7001428 -1.6444027 2.2386633
C 0.2659647 -0.7096000 2.9570880
H -0.6083409 0.0772054 -2.3610242
H -1.9832015 1.8227511 -1.5030707
H 2.5546340 -1.5492297 -2.1566502
H 2.9600985 0.7813163 -2.2574094
H -3.1926655 -2.2131926 1.4398482
H 2.8125638 -0.1672322 2.8855336
H -1.2618114 -2.2512618 2.9727184
H 1.0005708 -1.2897868 3.5379409
N -1.6053080 -0.7890618 1.4358457
N 0.9554346 0.2115329 1.9915216
O 0.5664660 2.1573897 -0.8046758
Ru -0.4067886 0.4882972 0.2789970
H -4.1767692 1.3756059 -0.6079951
H -4.7842315 -0.5737622 0.6958257
H -2.5378163 -3.3095298 -0.5825716
H -1.3210846 -2.2199259 -2.3709195
H 2.8902621 -2.9304592 -0.2350311
H 2.8433353 -2.2979761 2.0034323
H 3.5795642 1.7421147 1.8178674
H 3.7784217 2.1258077 -0.4685945
C 0.3515986 2.4685825 0.4005534
H -1.0070108 1.9721859 0.9618248
O 0.8099823 3.2331111 1.2328105
H 0.9674355 1.1534706 2.4049974
H -2.0872442 -0.1421555 2.0725842
H 0.3606463 -0.8553152 -0.1772951
H -0.1692049 -2.3096336 1.5456827
H -0.2956536 -0.0879156 3.6720103

4-F

41

Energy = -827.7762390980

C -1.2500469 -0.5922043 -0.9749283

C	-1.5297920	0.8368493	-1.0817815
C	1.7943862	0.2197967	-0.5180874
C	-2.2159532	-1.5404770	-0.4078701
C	-2.7648494	1.4698323	-0.6093575
C	1.3602875	1.6080913	-0.6451815
C	2.9013620	-0.1787882	0.3590735
C	-3.0601540	-1.3130400	0.6229043
C	1.9470012	2.7243617	0.1027378
C	-3.4748834	1.1324396	0.4886735
C	3.2071074	0.3519517	1.5635887
C	-3.0575040	-0.0047231	1.3820920
C	2.3994789	2.6931607	1.3751457
C	2.3600210	1.4328985	2.1972934
C	-1.4831658	1.2683929	2.9296251
C	0.0099646	1.6649569	3.1531323
H	-0.6404069	-1.0083936	-1.7815747
H	-1.0974766	1.3267537	-1.9582932
H	1.7126042	-0.3780368	-1.4294459
H	1.0018513	1.8911350	-1.6384741
H	-3.7399819	-0.0713020	2.2465603
H	2.7208866	1.6407495	3.2190342
H	-2.0301122	2.1355787	2.5385707
H	0.2891278	1.5598442	4.2164691
N	-1.6406297	0.1955796	1.9091668
N	0.9421059	0.8800583	2.2968780
Ru	-0.0929656	0.4987958	0.4678423
H	-3.1170223	2.3202125	-1.2015269
H	-4.3862902	1.6749262	0.7422740
H	-3.7722489	-2.0788889	0.9321084
H	-2.2479169	-2.5271774	-0.8802144
H	3.5385345	-0.9866275	-0.0146173
H	4.0859320	0.0087249	2.1107498
H	2.8219675	3.5889797	1.8311083
H	1.9870496	3.6803488	-0.4282487
H	0.1501898	2.7138798	2.8632152
H	-0.5390854	2.0826537	0.6307414
H	-1.3819282	-0.7113107	2.3121122
H	-1.9544969	0.9620459	3.8803218
H	1.0559347	-0.0649557	2.6782609
H	0.2739709	-1.0936197	0.8452030

4-F’

41

Energy = -827.7740616408

C	-1.2138912	-1.0473575	-1.4986755
C	-1.4242988	0.3423266	-1.1056019
C	1.8730353	-0.6968472	-1.1258511
C	-2.1984576	-2.0952255	-1.2019193
C	-2.5554511	0.8070773	-0.2911340
C	1.6721910	0.7240394	-0.8605024
C	2.9001018	-1.5212931	-0.4737357
C	-2.9455423	-2.2070373	-0.0801484
C	2.5733072	1.4847384	0.0137132
C	-3.1653859	0.1334884	0.7084627
C	3.3491690	-1.4033326	0.7947139
C	-2.7560088	-1.2636352	1.0904130
C	3.1606832	1.0435792	1.1494167
C	2.8477285	-0.3258930	1.7181195
C	-0.8190294	-1.0417530	2.8095524
C	0.7073592	-1.2501410	2.8659977
H	-0.6980108	-1.1850486	-2.4528833
H	-1.0574296	1.0892389	-1.8144043
H	1.6286054	-1.0222163	-2.1403486
H	1.2858949	1.3024016	-1.7044499
H	-3.3456320	-1.6116461	1.9552453
H	3.3051464	-0.4343332	2.7159604
H	-1.0725429	0.0093228	3.0128051
N	-1.2790904	-1.3651204	1.4385716
N	1.3346728	-0.3466297	1.8736845
Ru	0.1384656	-0.4726034	0.0908353
H	-2.8974905	1.8250043	-0.5010550
H	-3.9927510	0.5908023	1.2516053
H	-3.6976499	-2.9915629	0.0102973
H	-2.3449946	-2.8473695	-1.9833146
H	3.3003606	-2.3389040	-1.0808666
H	4.1114963	-2.0862982	1.1713602
H	3.8688868	1.6731535	1.6891527
H	2.7990683	2.5061442	-0.3074222
H	1.0965996	0.6162636	2.1349240
H	0.9663849	-2.2842293	2.5938773
H	-1.3183975	-1.6724923	3.5671549
H	1.0804106	-1.0562625	3.8881285
H	-0.2491038	1.0217880	0.7104777
H	0.4864407	-2.0963650	0.0005983
H	-1.0472664	-2.3477144	1.2574504

4-TS4'

41

Energy = -827.7387419806

C	-1.3053870	-0.4917014	-1.0375624
C	-1.5785709	0.9242707	-1.0579178
C	1.8125362	0.1353428	-0.4569350
C	-2.2555160	-1.4675390	-0.4928713
C	-2.8073170	1.5398830	-0.5460566
C	1.4007946	1.5072282	-0.6818602
C	2.8881400	-0.2255450	0.4685704
C	-3.0553612	-1.2734715	0.5771153
C	1.9810519	2.6578168	0.0137194
C	-3.4930486	1.1462522	0.5490883
C	3.1783273	0.3774174	1.6441567
C	-3.0057355	-0.0040060	1.4067396
C	2.3889046	2.6943303	1.3022096
C	2.3199180	1.4896746	2.2041769
C	-1.4936395	1.1647032	3.0074981
C	-0.0535687	1.7413361	3.1252263
H	-0.6732219	-0.8656154	-1.8486250
H	-1.1197367	1.4770095	-1.8813749
H	1.7151138	-0.5309180	-1.3178070
H	1.0577746	1.7305709	-1.6963637
H	-3.6785891	-0.1197442	2.2755997
H	2.6687035	1.7615845	3.2144425
H	-2.1731352	2.0225436	2.8447948
H	0.2735296	1.7864877	4.1793894
N	-1.6273092	0.2012175	1.9051406
N	0.9094380	0.9407724	2.3175873
Ru	-0.1124019	0.5128586	0.4970541
H	-3.1705909	2.4062134	-1.1067423
H	-4.4110826	1.6676781	0.8295603
H	-3.7591827	-2.0511596	0.8768049
H	-2.3011827	-2.4315331	-1.0080079
H	3.5212830	-1.0631453	0.1598753
H	4.0492408	0.0657279	2.2220691
H	2.8150720	3.6102534	1.7127091
H	2.0524980	3.5814680	-0.5679022
H	-0.0174090	2.7568368	2.7123531
H	-0.4640916	2.0658616	0.4904072
H	-0.6819994	-0.8724838	1.6303991
H	-1.7947824	0.6926317	3.9632563
H	1.0204646	0.0175165	2.7535496
H	0.0629225	-1.2651720	1.1418142

4-D1
44
Energy = -1016.4945279820
C -1.3469419 -0.6931153 -0.8668031
C -1.6029901 0.7074839 -1.0831953
C 1.7211896 0.1699874 -0.5100853
C -2.3088961 -1.5797946 -0.2140116
C -2.8303608 1.3897195 -0.6548969
C 1.3629737 1.5413459 -0.7535930
C 2.7162919 -0.2493948 0.4738193
C -3.1470609 -1.2513749 0.7934739
C 2.1031493 2.6603689 -0.1647280
C -3.5113467 1.1538096 0.4866711
C 3.0023266 0.3603204 1.6461009
C -3.0805251 0.0991562 1.4775633
C 2.6142377 2.7152525 1.0876053
C 2.3237671 1.6271606 2.0984520
C -1.3984825 1.1843488 3.0896662
C 0.1105583 1.1103482 3.3868151
H -0.6956682 -1.1831796 -1.5947826
H -1.1467406 1.1389537 -1.9782126
H 1.5668825 -0.5300944 -1.3343631
H 0.9570814 1.7546559 -1.7456855
H -3.7519300 0.1076008 2.3517074
H 2.6887613 1.9225334 3.0948957
H -1.6962903 2.2085841 2.8140485
H 0.3911494 0.0774069 3.6300008
N -1.6648242 0.2624019 1.9575632
N 0.8116098 1.5237669 2.1430951
Ru -0.1550272 0.5367812 0.5075634
H -3.1996273 2.1724407 -1.3238670
H -4.4348882 1.6981654 0.6904890
H -3.8610796 -1.9810867 1.1748599
H -2.3446864 -2.6052657 -0.5908104
H 3.2353918 -1.1842876 0.2538613
H 3.7757537 -0.0533821 2.2941377
H 3.2336300 3.5575345 1.3976557
H 2.2821239 3.5132649 -0.8256042
H 0.3774247 1.7671768 4.2329882
H -0.6375781 2.0249564 0.3415803
H -1.3882443 -0.6892981 2.3515644
H -1.9726368 0.8839129 3.9830938
O 0.4760087 -1.6062246 1.1677013
C 0.0933158 -2.3210644 2.1486135
H 0.5593407 -3.3367386 2.1860009
O -0.7240436 -2.0184303 3.0562542
H 0.5060217 2.4829773 1.9456725
4-TS3'
44
Energy = -1016.4728929650
C -1.3204333 -0.5389550 -0.9963931
C -1.6539680 0.8696406 -1.1376230
C 1.6903631 0.2206982 -0.5638519
C -2.2527578 -1.5054758 -0.4055096

C	-2.8941059	1.4736560	-0.6302918
C	1.3744478	1.6141948	-0.8341056
C	2.6990925	-0.2229128	0.4078622
C	-3.0908957	-1.2853944	0.6320603
C	2.1317540	2.7148686	-0.2278383
C	-3.5471319	1.1401420	0.5030857
C	3.0071342	0.3715953	1.5803949
C	-3.0755770	0.0232224	1.3972098
C	2.6303086	2.7549586	1.0289149
C	2.3482000	1.6497603	2.0260161
C	-1.3490904	1.0296072	3.0520489
C	0.1723756	0.9929152	3.2862746
H	-0.6952636	-0.9550667	-1.7910162
H	-1.2601293	1.3572884	-2.0327043
H	1.5535643	-0.4679824	-1.4009599
H	1.0227144	1.8243742	-1.8476927
H	-3.7227557	-0.0521136	2.2865385
H	2.7134339	1.9387624	3.0251602
H	-1.6896205	2.0561354	2.8504013
H	0.5107783	-0.0446725	3.4211154
N	-1.6397237	0.1966407	1.8587479
N	0.8327613	1.5434072	2.0773341
Ru	-0.1675988	0.6803767	0.3848214
H	-3.2936062	2.3001980	-1.2253313
H	-4.4670716	1.6583156	0.7767956
H	-3.7850337	-2.0605697	0.9572164
H	-2.2680882	-2.4964865	-0.8688740
H	3.2042652	-1.1608552	0.1623307
H	3.7730150	-0.0593967	2.2260762
H	3.2419453	3.5964107	1.3559907
H	2.3221600	3.5761477	-0.8751835
H	0.4377052	1.5682647	4.1909788
H	-0.6921549	2.2417323	0.3652932
H	-1.3405724	-0.7588324	2.0979378
H	-1.8808354	0.6541274	3.9442785
H	0.3115092	-0.8585702	0.8863138
C	0.3722443	-2.6077276	1.4756747
O	-0.2233459	-2.4636274	2.4970443
O	0.9810584	-3.1004724	0.5930868
H	0.5135616	2.5117965	1.9647289

4-E’

44

Energy = -1016.4803377720

C	-1.3538325	-0.5287853	-1.0415555
C	-1.6628883	0.8759266	-1.1452138
C	1.6949963	0.2319631	-0.6064788
C	-2.2765615	-1.4959964	-0.4468904
C	-2.8951970	1.4786125	-0.6220686
C	1.3855902	1.6196238	-0.8503602
C	2.6797342	-0.2351310	0.3713305
C	-3.0932437	-1.2802803	0.6070520
C	2.1367466	2.7120367	-0.2292037
C	-3.5319325	1.1269606	0.5153138
C	2.9856533	0.3507108	1.5499328

C -3.0393992 0.0077628 1.4018291
C 2.6331017 2.7338913 1.0298067
C 2.3367178 1.6265372 2.0169614
C -1.3416710 0.9906732 3.0615684
C 0.1767959 0.9697549 3.2974132
H -0.7162468 -0.9359039 -1.8306704
H -1.2510353 1.3880009 -2.0183519
H 1.5336124 -0.4519106 -1.4431284
H 1.0070488 1.8490865 -1.8497309
H -3.6780386 -0.0754738 2.2958392
H 2.7095205 1.8999269 3.0170222
H -1.7033080 2.0178380 2.8982377
H 0.5196399 -0.0651369 3.4353035
N -1.6077390 0.1769258 1.8470038
N 0.8229692 1.5244270 2.0776733
Ru -0.1671895 0.6600081 0.3907295
H -3.3108207 2.3002678 -1.2119408
H -4.4685533 1.6156201 0.7880559
H -3.7776924 -2.0589271 0.9421753
H -2.2907834 -2.4865071 -0.9087046
H 3.1510588 -1.1922394 0.1421783
H 3.7400705 -0.0994076 2.1955214
H 3.2548265 3.5651014 1.3639050
H 2.3324188 3.5791390 -0.8665990
H 0.4450255 1.5553635 4.1933750
H -0.6368806 2.1751788 0.2952559
H -1.2835997 -0.8052531 2.1073667
H -1.8663812 0.5733331 3.9381957
H 0.3088835 -1.1197152 0.8654764
C 0.3665922 -2.1966292 1.4836579
O -0.4446693 -2.2136412 2.4330631
O 1.2038013 -2.9401944 0.9995107
H 0.5074918 2.4965882 1.9845000

4-G

41

Energy = -827.7740616408

C -1.2138912 -1.0473575 -1.4986755
C -1.4242988 0.3423266 -1.1056019
C 1.8730353 -0.6968472 -1.1258511
C -2.1984576 -2.0952255 -1.2019193
C -2.5554511 0.8070773 -0.2911340
C 1.6721910 0.7240394 -0.8605024
C 2.9001018 -1.5212931 -0.4737357
C -2.9455423 -2.2070373 -0.0801484
C 2.5733072 1.4847384 0.0137132
C -3.1653859 0.1334884 0.7084627
C 3.3491690 -1.4033326 0.7947139
C -2.7560088 -1.2636352 1.0904130
C 3.1606832 1.0435792 1.1494167
C 2.8477285 -0.3258930 1.7181195
C -0.8190294 -1.0417530 2.8095524
C 0.7073592 -1.2501410 2.8659977
H -0.6980108 -1.1850486 -2.4528833
H -1.0574296 1.0892389 -1.8144043

H	1.6286054	-1.0222163	-2.1403486
H	1.2858949	1.3024016	-1.7044499
H	-3.3456320	-1.6116461	1.9552453
H	3.3051464	-0.4343332	2.7159604
H	-1.0725429	0.0093228	3.0128051
N	-1.2790904	-1.3651204	1.4385716
N	1.3346728	-0.3466297	1.8736845
Ru	0.1384656	-0.4726034	0.0908353
H	-2.8974905	1.8250043	-0.5010550
H	-3.9927510	0.5908023	1.2516053
H	-3.6976499	-2.9915629	0.0102973
H	-2.3449946	-2.8473695	-1.9833146
H	3.3003606	-2.3389040	-1.0808666
H	4.1114963	-2.0862982	1.1713602
H	3.8688868	1.6731535	1.6891527
H	2.7990683	2.5061442	-0.3074222
H	1.0965996	0.6162636	2.1349240
H	0.9663849	-2.2842293	2.5938773
H	-1.3183975	-1.6724923	3.5671549
H	1.0804106	-1.0562625	3.8881285
H	-0.2491038	1.0217880	0.7104777
H	0.4864407	-2.0963650	0.0005983
H	-1.0472664	-2.3477144	1.2574504

4'$_m$-cis
39
Energy = -826.5884995280

C	-1.2001784	-0.7944237	-1.4881275
C	-1.5451478	0.5276421	-1.0169988
C	1.7381041	-0.8249406	-1.0228717
C	-2.0399603	-1.9639828	-1.2414075
C	-2.7638564	0.8020130	-0.2530697
C	1.7575082	0.6326337	-0.9624425
C	2.6734934	-1.6895762	-0.3055095
C	-2.7340309	-2.1978946	-0.1058616
C	2.7177472	1.3741497	-0.1546132
C	-3.2925872	-0.0119181	0.6863698
C	3.1915103	-1.4736939	0.9246090
C	-2.5813790	-1.2928047	1.1069355
C	3.2341904	1.0001787	1.0424847
C	2.8096580	-0.2684036	1.7437347
C	-0.7722566	-1.1575032	2.8211584
C	0.7514784	-1.2607873	2.8580077
H	-0.6417215	-0.8226932	-2.4285751
H	-1.2093180	1.3515522	-1.6509823
H	1.4213028	-1.2377688	-1.9844706
H	1.4494076	1.1401821	-1.8810529
H	-3.1004781	-1.7382528	1.9715103
H	3.2894757	-0.3248956	2.7353685
H	-1.1155870	-0.2714657	3.3996825
N	-1.1626334	-1.0834271	1.4163676
N	1.3067140	-0.2205189	1.9474145
Ru	0.0772837	-0.2442623	0.1948561
H	-3.2918176	1.7276182	-0.4988863
H	-4.2681136	0.2051368	1.1255955
H	-3.4342895	-3.0319598	-0.0401059
H	-2.1297824	-2.6790687	-2.0638046
H	2.9663913	-2.6069427	-0.8255780
H	3.9146102	-2.1723453	1.3468633
H	3.9895893	1.6172699	1.5300278
H	3.0502783	2.3337518	-0.5627468
H	1.1075945	0.7044211	2.3469487
H	1.0669633	-2.2337323	2.4562039
H	-1.2106608	-2.0448672	3.3156652
H	1.1533888	-1.1494809	3.8808448
H	0.0331387	1.3137571	0.3932202

4-TS4

41

Energy = -827.7387982619

C	-1.2422835	-0.6819016	-0.9132709
C	-1.5052826	0.7285891	-1.1225979
C	1.8108559	0.3390494	-0.5707153
C	-2.2146638	-1.5832976	-0.2925831
C	-2.7357347	1.4008902	-0.7013957
C	1.3512239	1.7053534	-0.6179526
C	2.9243415	-0.0862053	0.2839554
C	-3.0728402	-1.2806199	0.7084812
C	1.9263069	2.8030675	0.1662730
C	-3.4461328	1.1460345	0.4199390
C	3.1983516	0.3937251	1.5152347
C	-3.0541432	0.0669848	1.3956353
C	2.3863678	2.7142940	1.4335264
C	2.3094358	1.4119346	2.2046166
C	-1.4571256	1.3730776	2.9103104
C	0.0491731	1.5567128	3.2494992
H	-0.6009172	-1.1558517	-1.6610183
H	-1.0512951	1.1582273	-2.0201904
H	1.6933617	-0.2341978	-1.4949400
H	0.9266276	2.0233535	-1.5732650
H	-3.7544206	0.0649445	2.2479106
H	2.6842860	1.5739555	3.2311451
H	-1.8498816	2.2735900	2.4226755
H	0.2558808	1.1927412	4.2749966
N	-1.6490447	0.2648289	1.9329652
N	0.9305284	0.8796459	2.2869320
Ru	-0.0911009	0.5175218	0.4984247
H	-3.0831778	2.2111472	-1.3493854
H	-4.3563935	1.7096482	0.6273799
H	-3.8037699	-2.0155027	1.0476017
H	-2.2486119	-2.5991200	-0.6979507
H	3.5743127	-0.8646419	-0.1268179
H	4.0789717	0.0387301	2.0526287
H	2.8137448	3.5952676	1.9176438
H	1.9560514	3.7756227	-0.3337796
H	0.2503466	2.6441564	3.2485048
H	-0.5531499	2.0407738	0.4717741
H	-1.4161373	-0.6245823	2.3911409
H	-2.0512057	1.1893626	3.8237074
H	0.7471830	-0.4925290	1.8408063
H	0.4528145	-1.1613274	1.1974354

TS5
37
Energy = -825.3364627212

C	-1.0691086	-0.7808896	-1.6971364
C	-1.6473329	0.4540249	-1.1930589
C	1.7768586	-0.8127481	-0.7745422
C	-1.7867511	-2.0582818	-1.6605771
C	-2.9711357	0.5091393	-0.5671817
C	1.7309036	0.6482177	-0.7718034
C	2.7035255	-1.5865207	0.0617471
C	-2.5726836	-2.4850079	-0.6498759
C	2.5211602	1.4419112	0.1672805
C	-3.5050240	-0.4412258	0.2300303
C	3.0192142	-1.3247704	1.3469204
C	-2.7145099	-1.6797617	0.6308138
C	2.8155641	1.1047475	1.4467126
C	2.3390570	-0.1841368	2.0863018
C	-1.0868976	-1.5250964	2.4713111
C	0.1448945	-1.0665054	2.8954684
H	-0.4061652	-0.6601156	-2.5600795
H	-1.3551565	1.3595152	-1.7290945
H	1.5886329	-1.2721176	-1.7482262
H	1.5492538	1.1201967	-1.7412444
H	-3.2728168	-2.2562707	1.3852053
H	2.6123236	-0.1985451	3.1534357
H	-1.8038743	-2.0098128	3.1390150
H	0.5194935	-1.1717384	3.9151823
N	-1.3895459	-1.3105000	1.1584470
N	0.8705853	-0.3665862	1.9639685
Ru	-0.0846269	-0.2410365	0.1529456
H	-3.5788275	1.3885171	-0.7982106
H	-4.5393598	-0.3706615	0.5687680
H	-3.1541163	-3.4031100	-0.7393467
H	-1.6886690	-2.6933899	-2.5449695
H	3.1899669	-2.4344872	-0.4282234
H	3.7902941	-1.8931493	1.8684037
H	3.4471088	1.7556031	2.0518331
H	2.8903089	2.4044038	-0.1989826
H	0.2554741	0.8536975	1.4879289
H	-0.1061176	1.3560239	0.5975290

G
37
Energy = -825.3586149362

C	-1.0424298	-0.8978938	-1.6037121
C	-1.6604795	0.3686531	-1.2761124
C	1.7946867	-0.6767149	-0.7968439
C	-1.7379600	-2.1827936	-1.4829809
C	-2.9839157	0.4899079	-0.6693894
C	1.7758684	0.7670742	-0.6578182
C	2.7383393	-1.5354328	-0.0715479
C	-2.5635917	-2.5256126	-0.4746586
C	2.5593205	1.4698354	0.3590704
C	-3.5260710	-0.3823512	0.2089173
C	3.0798906	-1.3992632	1.2236220
C	-2.7599606	-1.5923709	0.7036876
C	2.8436648	0.9957321	1.5921600
C	2.3695502	-0.3602101	2.0741461
C	-1.1009704	-1.5134330	2.4949990
C	0.2073114	-1.1681473	2.8527193
H	-0.3498486	-0.8529963	-2.4489912
H	-1.3645949	1.2095246	-1.9077665
H	1.5770619	-1.0356234	-1.8039941
H	1.5941945	1.3270116	-1.5786987
H	-3.3296876	-2.0982519	1.4986036
H	2.6284389	-0.4934958	3.1365295
H	-1.8024954	-2.0215261	3.1599471
H	0.6527505	-1.4048501	3.8223858
N	-1.4306053	-1.1949686	1.2307155
N	0.9122133	-0.5498263	1.8955165
Ru	-0.1011489	-0.1254263	0.2239976
H	-3.5728074	1.3658703	-0.9571408
H	-4.5450614	-0.2448614	0.5724563
H	-3.1121667	-3.4678248	-0.4839321
H	-1.5797472	-2.8955202	-2.2968460
H	3.2149492	-2.3221091	-0.6626787
H	3.8594505	-2.0087491	1.6813946
H	3.4718069	1.5747604	2.2701818
H	2.9197380	2.4676723	0.0930301
H	-0.6169043	1.4569531	0.8156376
H	0.0131111	1.6267905	0.2400887

TS-4$_m$>4'$_m$

39

Energy = -826.5550437798

C	-1.3266359	0.0431860	-1.5700977
C	-1.5800404	1.4644126	-1.3254430
C	1.6421300	0.3364271	-0.6252639
C	-2.2971277	-1.0009671	-1.2668662
C	-2.8324151	1.9836013	-0.7800697
C	1.3837040	1.7540477	-0.8119906
C	2.5970961	-0.1664937	0.3661262
C	-3.2060093	-1.0127889	-0.2565323
C	2.0401241	2.7854653	-0.0154795
C	-3.6164064	1.3973177	0.1543768
C	2.7985430	0.3447801	1.6019663
C	-3.2622480	0.0756230	0.7892347
C	2.3502625	2.6908089	1.3012420
C	1.9392711	1.4865493	2.1327568
C	-1.7897384	0.9291569	2.7001332
C	-0.3212250	0.9270239	3.1454111
H	-0.6896368	-0.1720036	-2.4352470
H	-1.1011348	2.1454774	-2.0355465
H	1.5627226	-0.2766426	-1.5288640
H	1.1352729	2.0580630	-1.8335666
H	-4.0115378	-0.1805684	1.5571025
H	2.1692983	1.6735126	3.1944292
H	-2.1217424	1.9498771	2.4635125
H	-0.0676859	-0.0616540	3.5842489
N	-1.8960427	0.1394941	1.4402595
N	0.5073915	1.2025487	1.9783368
Ru	-0.2794298	0.7517198	0.1821956
H	-3.1547411	2.9556507	-1.1660325
H	-4.5562246	1.8645191	0.4513134
H	-3.9226637	-1.8308010	-0.1747945
H	-2.2909622	-1.8699008	-1.9332314
H	3.2187635	-1.0093831	0.0494004
H	3.6183090	-0.0173450	2.2256991
H	2.9122783	3.4845129	1.7952409
H	2.3046658	3.7107638	-0.5363271
H	-0.1808616	1.6765190	3.9486271
H	-0.1365759	2.1295232	1.0701418
H	-1.6408579	-0.8359281	1.6478009
H	-2.4455220	0.5220490	3.4894317

4$_m$
39
Energy = -826.5808257125

C	-1.3082652	-0.0340241	-1.5685074
C	-1.5698675	1.3976292	-1.4359143
C	1.5542067	0.3881797	-0.6549952
C	-2.2886335	-1.0614702	-1.2422323
C	-2.7901778	1.9548390	-0.8537289
C	1.3765468	1.8238599	-0.8626311
C	2.3520160	-0.1882436	0.4263762
C	-3.2133660	-1.0308980	-0.2474370
C	2.0755626	2.8381960	-0.0848526
C	-3.5634635	1.4079320	0.1178332
C	2.5626939	0.3315808	1.6617580
C	-3.2439687	0.0823948	0.7706476
C	2.4132328	2.7799563	1.2298035
C	1.9606247	1.6430420	2.1133068
C	-1.7546150	0.8028504	2.7163598
C	-0.2721107	0.8854732	3.0943070
H	-0.6501922	-0.3022154	-2.4030689
H	-1.1174130	2.0315821	-2.2075206
H	1.5303027	-0.2243627	-1.5637125
H	1.1942380	2.1149639	-1.9033992
H	-3.9963076	-0.1409996	1.5465846
H	2.2495548	1.8441499	3.1595146
H	-2.1800267	1.8125221	2.6045139
H	0.1611684	-0.1239919	3.1758503
N	-1.8687621	0.1253226	1.4015749
N	0.4520277	1.5924980	2.0099040
Ru	-0.3038801	0.8770029	0.1175972
H	-3.0979591	2.9317837	-1.2399201
H	-4.4762443	1.9167682	0.4319700
H	-3.9395700	-1.8374976	-0.1420653
H	-2.2801393	-1.9531573	-1.8783909
H	2.8148038	-1.1570765	0.2113402
H	3.2188347	-0.1882679	2.3615090
H	2.9959121	3.5799928	1.6872879
H	2.3660527	3.7443681	-0.6277009
H	-0.1457047	1.3848870	4.0724149
H	0.1348632	2.5698900	1.9750679
H	-1.5695448	-0.8530487	1.5009457
H	-2.3300625	0.2817426	3.5032443

4_m-cis

39

Energy = -826.5781667187

C	-1.1787969	-1.3279675	-1.4658998
C	-1.4061955	0.1008442	-1.6602892
C	1.7203057	-1.0217171	-1.3313040
C	-2.0723978	-2.2098827	-0.7228907
C	-2.5592022	0.8054908	-1.1138537
C	1.6810854	0.4286396	-1.4870819
C	2.6841294	-1.7345957	-0.5006938
C	-2.8044756	-1.8946822	0.3726789
C	2.6143372	1.3269507	-0.8175825
C	-3.2170369	0.5327764	0.0434286
C	3.2221055	-1.3136927	0.6698475
C	-2.7463475	-0.5284895	1.0059101
C	3.1897263	1.1561306	0.4009741
C	2.8367854	-0.0011445	1.3018607
C	-0.7456704	-0.9679207	2.5092137
C	0.8092077	-0.8847794	2.5498216
H	-0.6775947	-1.8276339	-2.3023243
H	-1.0464161	0.5056960	-2.6127317
H	1.4151244	-1.5853655	-2.2204132
H	1.3470766	0.7841905	-2.4682322
H	-3.3687580	-0.5126173	1.9172200
H	3.3488049	0.1082565	2.2739220
H	-1.0512044	-2.0099211	2.3503661
H	1.2404855	-1.8654015	2.3126249
N	-1.3123566	-0.1883933	1.3752202
N	1.3344829	0.0524253	1.5223663
Ru	0.1142105	-0.2309840	-0.2059706
H	-2.9370535	1.6431445	-1.7098864
H	-4.1067051	1.1030862	0.3141320
H	-3.4772682	-2.6314080	0.8134303
H	-2.1471519	-3.2373078	-1.0938553
H	2.9927248	-2.7198742	-0.8643225
H	3.9691975	-1.9222421	1.1803948
H	3.9213183	1.8778276	0.7665374
H	2.8830827	2.2355151	-1.3671140
H	-1.1827662	-0.6276293	3.4651804
H	1.1569191	-0.5948253	3.5584775
H	-1.3243770	0.8141369	1.6103874
H	1.1331289	1.0249435	1.7943342

8. Supplementary References

[1] Hallman, P. S., Stephenson, T. A. & Wilkinson. G. Tetrakis(triphenylphosphine)dichlororuthenium(II) and Tris(triphenylphosphine)dichlororuthenium(II). *Inorg. Synth.* 12 (1970).

[2] Rodríguez-Lugo, R. E. *et al.* A homogeneous transition metal complex for clean hydrogen production from methanol-water mixtures *Nature Chem.* **5**, 342 (2013).

[3] Loginovaa, D., Lileeva, A., Lyashchenkoa, A., Aladko, L. The high-frequency dielectric spectroscopy of the aqueous solutions of tetrabutylammonium carboxylates. *J. Non-Crystall. Sol.* 351, 2882 (2005).

[4] Ahlrichs, R. *Turbomole Version 6.5*, (2013), Theoretical Chemistry Group, University of Karlsruhe.

[5] *PQS version 2.4, 2001, Parallel Quantum Solutions, Fayettevile, Arkansas (USA); the Baker optimizer is available separately from PQS upon request*: I. Baker, *J. Comput. Chem.* 7, 385 (1986).

[6] Budzelaar, P. H. M. Geometry optimization using generalized, chemically meaningful constraints. *J. Comput. Chem.* **28**, 2226 (2007).

[7] Becke, A. D. Density-functional exchange-energy approximation with correct asymptotic behavior. *Phys. Rev. A*, **38**, 3098 (1988).

[8] Perdew, J. P. Density-functional approximation for the correlation energy of the inhomogeneous electron gas *Phys. Rev. B* **33**, 8822 (1986).

[9] Sierka, M., Hogekamp, A. & Ahlrichs, R. *J.* Fast Evaluation of the Coulomb Potential for Electron Densities Using Multipole Accelerated Resolution of Identity Approximation *Chem. Phys.* **118**, 9136 (2003).

[10] Schaefer, A., Horn, H. & Ahlrichs, R. Fully optimized contracted Gaussian basis sets for atoms Li to Kr. *J. Chem. Phys.* **97**, 2571 (1992).

[11] Grimme, S., Antony, J., Ehrlich, S. & Krieg, H. A consistent and accurate ab initio parametrization of density functional dispersion correction (DFT-D) for the 94 elements H-Pu. *J. Chem. Phys.* **132**, 154104 (2010).

[12] Dzik, W. I., Xu, X., Zhang, X. P., Reek, J. N. H. & de Bruin, B. Carbene Radicals in CoII(por)-Catalyzed Olefin Cyclopropanation. *J. Am. Chem. Soc.* **132**, 10891 (2010).

[13] Li, H. & Hall, M. B. Role of the chemically non-innocent ligand in the catalytic formation of hydrogen and carbon dioxide from methanol and water with the metal as the spectator. *J. Am. Chem. Soc.* **137**, 12330 (2015).

[14] Y. Jing, X. Chen, X. Yang. Theoretical study of the mechanism of ruthenium catalyzed dehydrogenation of methanol-water mixture to H_2 and CO_2. *J. Organomet. Chem.* **820**, 55 – 61 (2016).

[15] Lam, Y.-H., Grayson, M. N., Holland, M. C., Simon, A. & Houk, K. N. Theory and Modeling of Asymmetric Catalytic Reactions. *Acc. Chem. Res.* **49**, 750 (2016).

[16] Plata, R. E. & Singleton, D. A. A Case Study of the Mechanism of Alcohol-Mediated Morita Baylis–Hillman Reactions. The Importance of Experimental Observations. *J. Am. Chem. Soc.* **137**, 3811 (2015).
